# Spread of Highly Pathogenic Avian Influenza (HPAI) H5N5 Viruses in Europe in 2016–2017 Appears Related to the Timing of Reassortment Events

**DOI:** 10.3390/v11060501

**Published:** 2019-05-31

**Authors:** Saskia A. Bergervoet, Cynthia K. Y. Ho, Rene Heutink, Alex Bossers, Nancy Beerens

**Affiliations:** 1 Department of Virology, Wageningen Bioveterinary Research, 8221 RA Lelystad, The Netherlands; saskia.bergervoet@wur.nl (S.A.B.); rene.heutink@wur.nl (R.H.); 2 Department of Infection Biology, Wageningen Bioveterinary Research, 8221 RA Lelystad, The Netherlands; cynthia.ho@wur.nl (C.K.Y.H.); alex.bossers@wur.nl (A.B.)

**Keywords:** avian influenza, highly pathogenic avian influenza, genetic analysis, reassortment

## Abstract

During the epizootic of highly pathogenic avian influenza (HPAI) H5N8 virus in Europe in 2016–2017, HPAI viruses of subtype H5N5 were also isolated. However, the detection of H5N5 viruses was limited compared to H5N8. In this study, we show that the genetic constellation of a newly isolated H5N5 virus is different from two genotypes previously identified in the Netherlands. The introduction and spread of the three H5N5 genotypes in Europe was studied using spatiotemporal and genetic analysis. This demonstrated that the genotypes were isolated in distinguishable phases of the epizootic, and suggested multiple introductions of H5N5 viruses into Europe followed by local spread. We estimated the timing of the reassortment events, which suggested that the genotypes emerged after the start of autumn migration. This may have prevented large-scale spread of the H5N5 viruses on wild bird breeding sites before introduction into Europe. Experiments in primary chicken and duck cells revealed only minor differences in cytopathogenicity and replication kinetics between H5N5 genotypes and H5N8. These results suggest that the limited spread of HPAI H5N5 viruses is related to the timing of the reassortment events rather than changes in virus pathogenicity or replication kinetics.

## 1. Introduction

Highly pathogenic avian influenza (HPAI) H5N1 viruses belonging to the A/Goose/Guangdong/1/1996 (GsGd) lineage were first detected in domestic geese in China in 1996 [1]. The virus caused outbreaks in poultry and humans in Hong Kong in 1997 [2], and was first detected in wild birds in 2002 [3]. The virus was subsequently disseminated intercontinentally from Asia to Europe, Africa and the Middle East by wild migratory birds in 2005 [4]. As descendants of the H5N1 GsGd lineage continued to circulate among poultry and wild birds, the hemagglutinin (HA) gene evolved into numerous phylogenetic clades. In addition, reassortment of H5N1 GsGd lineage viruses with co-circulating low pathogenic avian influenza (LPAI) viruses led to the emergence of new reassortant viruses of various gene constellations and subtype combinations. In 2014, HPAI H5 clade 2.3.4.4 viruses of subtype H5N8 emerged in Asia [5,6], which were divided into phylogenetic groups A and B. From 2014 onwards, several reassortant variants of HPAI H5 clade 2.3.4.4 viruses came to prominence, causing outbreaks of severe disease and high mortality among wild birds and commercial poultry worldwide.

In late 2014, HPAI H5N8 viruses belonging to clade 2.3.4.4 group A were introduced into Europe [7,8] and North America [9,10] by wild migratory birds. Intersubtype reassortment produced HPAI viruses of subtypes H5N1 and H5N2, the latter of which caused a large outbreak in commercial poultry in the United States in 2014–2015 [11]. In 2016, HPAI H5N8 viruses belonging to clade 2.3.4.4 group B re-emerged in Asia. The virus was first detected at the Qinghai Lake in China and the Ubsu-Nur Lake at the border between Russia and Mongolia, in May 2016 [12,13,14], and subsequently spread to Europe, Africa and the Middle East in the autumn and winter of 2016–2017 [15]. During this epizootic, over 2000 outbreaks in wild birds and poultry were reported in Europe [16]. In 2017, a reassortant virus of subtype H5N6 virus emerged from H5N8 clade 2.3.4.4 group B viruses in Asia [17], infecting wild birds and poultry in several European countries in the autumn and winter of 2017–2018 [18].

During the HPAI H5N8 clade 2.3.4.4 group B epizootic in 2016–2017, multiple reassortant variants of subtype H5N5 were detected in Europe [19,20,21,22,23,24,25,26]. However, the number of birds detected with HPAI H5N5 virus was limited compared to H5N8 [19]. After the first detection of HPAI H5N5 virus in the Kamchatka region of Russia in October 2016 [20], a total of 24 outbreaks were reported in 11 European countries [19,20,21,22,23,24,25,26], mainly affecting wild birds. Infections of poultry and captive birds were reported during eight outbreaks [23,24,25]. Genetic analysis suggested that different H5N5 variants were introduced into Europe [21,23,26]. These H5N5 viruses contained the same HA cleavage site (PLREKRRKR/GLF) as was observed in the majority of the H5N8 isolates [20,21,22,24], and showed intravenous pathogenicity index (IVPI) scores in chickens comparable to H5N8 [22,24]. However, the limited number of infected birds may suggest that the H5N5 viruses exhibit characteristics different from H5N8, such as decreased infectivity, transmissibility or pathogenicity.

Here, we describe the genetic analysis of a newly isolated HPAI H5N5 virus that is genetically distinct from two H5N5 viruses previously isolated in the Netherlands. The Dutch H5N5 viruses were genetically compared with HPAI H5N5 viruses detected in other European countries, and the timing of reassortment was estimated. This study demonstrates that the three H5N5 genotypes were isolated in overlapping, but distinguishable outbreak phases. Results suggest multiple introductions of H5N5 viruses into Europe followed by local spread. We observed variations in the estimated timing of reassortment that led to the emergence of the H5N5 genotypes. Experiments in primary chicken and duck cells showed only minor differences in cytopathogenicity and virus replication between H5N5 genotypes and H5N8. These findings suggests that the spread of H5N5 viruses in Europe is mainly driven by the timing of reassortment rather than changes in virus pathogenicity and replication kinetics.

## 2. Materials and Methods

### 2.1. Virus Detection and Sequencing

Virus detection and sequencing were performed for one newly isolated HPAI H5N5 strain from a goose found dead in Utrecht, the Netherlands, on 22 May 2017 (A/Go/NL-Utrecht/17006881-001/2017; H5N5-19), as described previously [21]. In short, RNA was isolated from a tracheal swab sample using the MagNA Pure 96 system (Roche, Basel, Switzerland) with the MagNA Pure 96 DNA and Viral NA Small Volume Kit (Roche). The sample was tested for the presence influenza A virus by using a real-time reverse transcription polymerase chain reaction targeting the matrix protein (MP) gene (M-PCR) [27], and subsequently tested in a H5 subtype-specific PCR as recommended by the European Union reference laboratory [28,29]. Sanger sequencing was performed to determine the pathogenicity and the neuraminidase (NA) subtype of the virus [30,31]. The whole genome sequence was generated by next-generation sequencing (NGS), as described previously [21]. Briefly, RNA was isolated from the swab sample using the High Pure Viral RNA Kit (Roche). Multi-segment amplification was performed using the SuperScript III One-Step RT-PCR System with the Platinum Taq DNA Polymerase High Fidelity kit (Thermo Fisher Scientific, Waltham, MA, USA) and purified universal primers [32]. Purified amplicons were sequenced by using the Illumina Nextera DNA Sample Preparation kit and the paired-end 200 Illumina MiSeq platform with a minimum sequence coverage of 1000 reads. The whole genome consensus sequence was generated by a reference-based method using the ViralProfiler-Workflow, an extension of the CLC Genomics Workbench version 11.0 (Qiagen, Hilden, Germany) [21], and submitted to the GISAID’s EpiFlu™ Database (https://www.gisaid.org) [33] (EPI_ISL_288411). The most closely related viruses to the newly isolated H5N5 strain were identified by BLAST on 10 May 2019 (Table S1).

### 2.2. Phylogenetic Analysis

Phylogenetic trees were generated for each gene segment separately using the nucleotide sequences of the newly isolated HPAI H5N5 strain and other HPAI H5N5 viruses isolated during the epizootic in 2016–2017. Detailed information on the HPAI H5N5 virus sequences used in this study is provided in Table S2. We included the top 20 non-H5N5 sequence matches from the GISAID’s EpiFlu Database for each H5N5 genotype to assess the origin of the gene segments. As a reference, HPAI H5N8 cluster representatives were included, representing clusters of H5N8 viruses isolated during the epizootic in 2016–2017. To select these cluster representatives, clustering of nucleotide sequences of around 675 HPAI H5N8 2016–2017 viruses available in the GISAID’s EpiFlu Database on 10 May 2019 was performed using CD-HIT version 4.6.8 [34,35]. A nucleotide sequence identity threshold value of 1.5% was used to define clusters. The cluster representatives and the number of H5N8 viruses within each cluster are listed in Table S3. After selecting the best fit model of nucleotide substitution, phylogenetic analysis was performed using the maximum likelihood (ML) method within the MEGA7 software package [36]. Trees were generated using the Tamura-Nei substitution model with a discrete gamma distribution (TN93+G) with five rate categories. Bootstrap support values above 70 (1,000 replicates) are shown at the branches.

### 2.3. Network Analysis

Network analysis was performed for viruses belonging to genotypes H5N5-GT2 and H5N5-GT3. For each virus, the full-length nucleotide sequences of the eight gene segments were concatenated and aligned in the software program DNA Alignment (Fluxus Technology) (http://www.fluxus-engineering.com). Sequence gaps were treated by complete deletion and ambiguous states were replaced by searching for the best replacement within the sequence having minimal distance. Phylogenetic networks were reconstructed using the median-joining method in the software program Network version 5 (Fluxus Technology) [37]. Networks were displayed in the software program Network Publisher version 2.1.1.2 (Fluxus Technology). The number of nucleotide substitutions between strains are shown as values near branches.

### 2.4. Molecular Clock Analysis

The time to the most recent common ancestor (TMRCA) was estimated for each gene segment of viruses belonging to genotypes H5N5-GT2 and H5N5-GT3. The sequences of HPAI H5N5 2016–2017 viruses were supplemented with HPAI H5N8 and H5N5 reference sequences and the top 100 most similar sequences of both genotypes obtained from the GISAID’s EpiFlu Database on 24 January 2018. For gene segments PB1 and NP, we estimated the TMRCA for genotypes H5N5-GT2 and H5N5-GT3 using the top 100 most similar sequences of each genotype separately obtained from the GISAID’s EpiFlu Database on 10 May 2019. Multiple sequence alignments were performed with MUSCLE version 3.8.31 [38] and curated in Aliview version 1.20 [39]. For each segment, ML trees were generated using MEGA7 [36] to select for the lineages of interest. Time-scaled phylogenies were reconstructed using the Bayesian Markov chain Monte Carlo (MCMC) as implemented in BEAST version 1.8.4 (http://beast.community/beast), as described previously [21]. This analysis was conducted using the SRD06 substitution model [40], the Bayesian Skyline coalescent model, and an uncorrelated log-normal relaxed molecular clock. The Bayesian MCMC analysis was run for 100,000,000 states and the effective sample size (ESS) was checked in Tracer version 1.6 (http://beast.bio.ed.ac.uk/Tracer). Maximum clade credibility (MCC) tree files were summarized in Tree Annotator version 1.8 with a burn-in of 10%. The TMRCA values were obtained from the MCC trees that were visualized in FigTree version 1.4.3 (http://tree.bio.ed.ac.uk/software/figtree).

### 2.5. Cell Cultures

Madin-Darby Canine Kidney (MDCK) cells were obtained from Philips-Duphar (Weesp, the Netherlands) and maintained in complete Dulbecco’s Modified Eagle Medium (DMEM) (Thermo Fisher Scientific) supplemented with 5% fetal calf serum (FCS) (Harlan Bioproducts for Science, Indianapolis, IN, USA) and 0.1% penicillin-streptomycin (Thermo Fisher Scientific). Primary chicken embryo fibroblast (CEF) and duck embryo fibroblast (DEF) cells were prepared from 11-day-old specific-pathogen free (SPF) chicken embryos (*Gallus gallus domesticus*) and seronegative 14-day-old commercial Pekin duck embryos (*Anas platyrhynchos domesticus*), respectively, as described previously [41]. Briefly, embryo head, limbs and internal organs were removed and a single-cell suspension was prepared by trypsinization. The primary chicken and duck cells were seeded in growth medium containing 1.0× Medium 199 with Earle’s salts (Thermo Fisher Scientific), 3.6% new born calf serum (NBCS) (Thermo Fisher Scientific), 0.12% sodium bicarbonate (Thermo Fisher Scientific), 2mM l-glutamine (Thermo Fisher Scientific), 0.1× MEM Vitamin solution (Thermo Fisher Scientific) and 0.1% gentamicin (Sigma-Aldrich, St. Louis, MO, USA). After one day, growth medium was replaced by maintenance medium containing 0.4× Medium 199 with Earle’s salts (Thermo Fisher Scientific), 3.0% new born calf serum (NBCS) (Thermo Fisher Scientific), 0.05× Ham’s F-10 Nutrient Mix (Thermo Fisher Scientific), 0.11% sodium bicarbonate (Thermo Fisher Scientific), 1mM l-glutamine (Thermo Fisher Scientific), 0.1× M.E.M. Vitamins solutions (Thermo Fisher Scientific), 0.1% gentamicin (Sigma-Aldrich) and 0.12% tryptose phosphate broth. When confluence was reached after 2–3 days, cells were trypsinized and stored in liquid nitrogen for later use.

### 2.6. Virus Propagation and Titration

The viruses used for the in vitro experiments represent genotypes H5N5-GT1, H5N5-GT2, H5N5-GT3 and European H5N8 NL-Zeewolde-like virus, and are listed in Table S4. The viruses were isolated from swab samples using embryonated chicken eggs (ECEs), as described previously [42]. Virus stocks of second passage allantoic fluids were stored in aliquots at −80 °C. The medium tissue culture infective dose (TCID50) titres of the virus stocks were determined by end-point titration in MDCK cells. In short, a total of 2.5 × 10^4^ MDCK cells/wells were seeded in culture medium into each well of a 96 well tissue culture plate. The following day, the infection medium was prepared by replacing FCS in culture medium by 0.3% bovine serum albumin (BSA). Cells were inoculated with ten-fold serial dilutions of the virus stocks in the infection medium. After two days, an immunoperoxidase monolayer assay (IPMA) was performed using in-house produced mouse anti-nucleoprotein (anti-NP) HB65 monoclonal antibodies and HRP-conjugated rabbit anti-mouse secondary antibodies (Dako, Glostrup, Denmark) on monolayers fixed in 4% paraformaldehyde solution [43]. TCID50 titres were calculated using the Reed and Muench method [44].

### 2.7. Virus Infection of Primary Chicken and Duck Cells

The cytopathogenic effect (CPE) of HPAI H5N5 and H5N8 viruses in primary CEF and DEF cells was measured using the real-time cell analysis (RTCA) system (xCelligence; Roche and ACEA Biosciences, San Diego, CA, USA) [45]. For this real-time monitoring assay, a total of 3.5 × 10^5^ cells was seeded in growth medium into each well of the eight well electronic tissue culture plate (E-plate) (ACEA Biosciences). After one day, cells were inoculated at a multiplicity of infection (MOI) of 0.001 in serum-free growth medium. Mock-infected cells were taken along as negative controls. The electrical impedance of the cell-covered electrodes, displayed as cell index (CI) value, was measured every 30 minutes. An increase in CI value indicates cell proliferation and adhesion, whereas a decrease in CI value indicates cell death. The CI values were normalized at 2 hpi to determine the time point when the CI value reaches half maximal (CI50) value. The experiment was carried out three times in duplicate.

To generate growth curves of viral replication in CEF cells, a total of 3.5 × 10^5^ cells was seeded in growth medium into each well of a 24-well tissue culture plate. After one day, cells were inoculated with virus at a MOI of 0.001 in serum-free growth medium. Supernatants were collected at four hour intervals from 2 to 42 hpi and stored at −80 °C until used for virus titration. TCID50 titres were determined by end-point titration in MDCK cells as described above. The experiment was carried out twice in triplicate. Results were expressed by the mean and its standard deviation (SD).

## 3. Results

### 3.1. Genetic Analysis of HPAI H5N5 Viruses in the Netherlands

We determined the whole genome sequence of a novel HPAI H5N5 virus that was isolated from a goose found dead in Utrecht, the Netherlands, in May 2017. To determine the gene constellation of this virus, the most closely related viruses were identified (Table S1), and phylogenetic trees were reconstructed for each gene segment separately (Figure S1). For phylogenetic analysis, we used the full-length nucleotide sequences of all HPAI H5N5 viruses isolated during the HPAI H5 2016–2017 epizootic (Table S2), and sequences of closely related viruses of other subtypes. In addition, HPAI H5N8 cluster representatives were included that represent the genetic diversity among H5N8 viruses during the epizootic (Table S3). The genetic analysis revealed that the newly isolated H5N5 strain was genetically distinct from two H5N5 viruses previously isolated in the Netherlands. This suggests that at least three different HPAI H5N5 reassortant variants circulated in the Netherlands, referred to as genotypes H5N5-GT1, H5N5-GT2 and H5N5-GT3.

**Figure 1:**
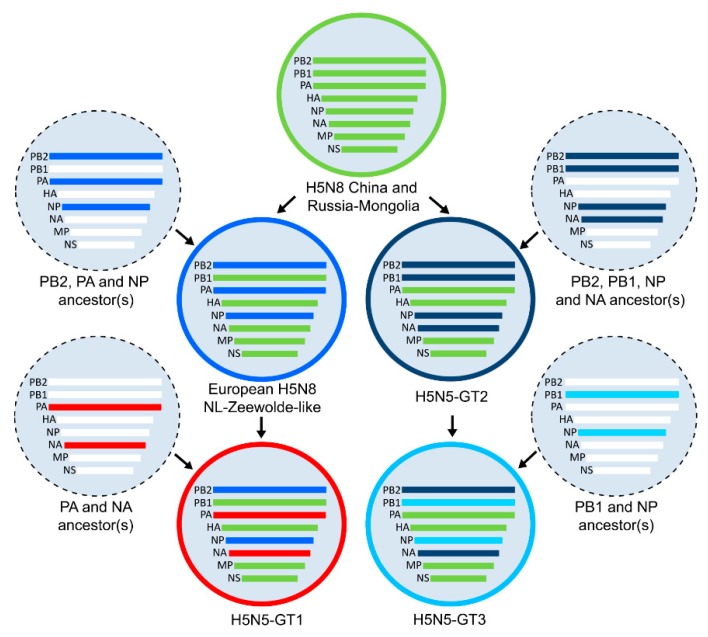
Gene constellations of HPAI H5N5 genotypes. Schematic representation of reassortment events that resulted in the emergence of three highly pathogenic avian influenza (HPAI) H5N5 genotypes detected during the HPAI H5 2016-2017 epidemic (H5N5-GT1, H5N5-GT2 and H5N5-GT3). Novel genes were obtained by reassortment of HPAI viruses with co-circulating low pathogenic avian influenza (LPAI) ancestor viruses. Gene segments are colored according to their phylogenetic cluster, as shown in Figure S1. PB2, polymerase basic protein 2; PB1, polymerase basic protein 1; PA, polymerase acidic protein; HA, hemagglutinin; NP, nucleoprotein; NA, neuraminidase; MP, matrix protein; NS, nonstructural protein.

The gene constellations of the genotypes are shown in Figure 1, where the gene segments are colored according to their phylogenetic cluster. Gene segments HA, MP and nonstructural protein (NS) of the three H5N5 genotypes clustered with the H5N8-China and Russia-Mongolia reference virus, whereas distinct clusters were identified for gene segments polymerase basic protein 1 (PB1), polymerase basic 2 (PB2), polymerase acidic (PA), NP and NA. The PB2 and NP genes of genotype H5N5-GT1 clustered with European H5N8 NL-Zeewolde-like viruses detected in the Netherlands (H5N8-PA I and H5N8-PA II), whereas the PA and NA genes are related to Eurasian LPAI viruses detected in previous years (Table S5). Genotype H5N5-GT2 contains reassorted PB2, PB1, NP and NA genes related to LPAI viruses detected in Eurasia in previous years. Genotype H5N5-GT3 is genetically highly similar to genotype H5N5-GT2, but contains reassorted PB1 and NP genes that were related to two LPAI viruses detected in the Netherlands in 2014.

### 3.2. Incidence and Spatiotemporal Distribution of HPAI H5N5 Genotypes in Europe

The viruses that were detected in the Netherlands were genetically compared to HPAI H5N5 viruses detected in other European countries to reveal the incidence of the genotypes (Table 1; Figure S2). Results show that genotype H5N5-GT1 consisted of a unique gene constellation that was detected only once (H5N5-1). Genotype H5N5-GT2 viruses were most frequently detected, as 13 viruses were identified in Europe during the epizootic in 2016–2017 (H5N5-3 to -15). This genotype was first identified in Kamchatka, Russia, in October 2016 (H5N5-2). Four viruses (H5N5-16 to -19) were identified for genotype H5N5-GT3. For two H5N5 viruses (H5N5-20 and -21), the genotype could not be determined because of insufficient sequence data. Overall, this analysis demonstrates that the H5N5-GT2 was the most frequently isolated genotype of H5N5 viruses in Europe, followed by H5N5-GT3.

**Figure 2:**
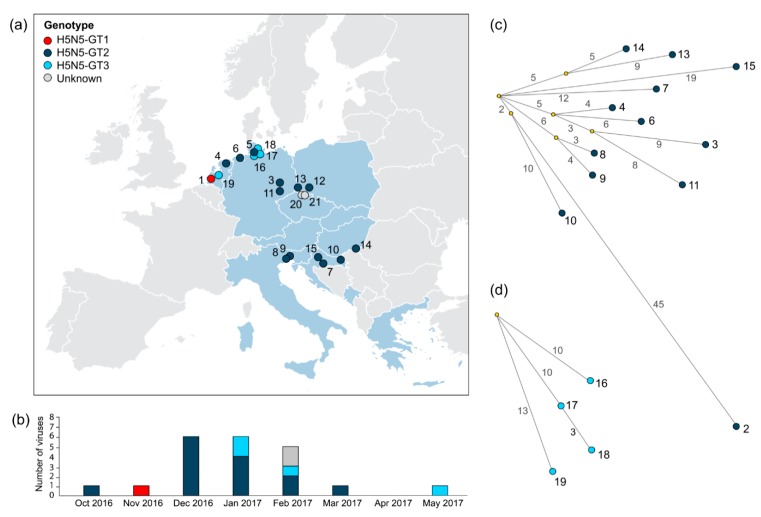
Spatiotemporal distribution and phylogenetic network analysis of HPAI H5N5 genotypes. (**a**) Map of Europe showing the geographical distribution of highly pathogenic avian influenza (HPAI) H5N5 viruses isolated during the HPAI H5 2016–2017 epidemic, with countries reporting HPAI H5N5 virus infection (blue) and the collection locations of HPAI H5N5 viruses, colored by genotype. (**b**) Number of HPAI H5N5 viruses isolated during the HPAI H5 2016–2017 epidemic per month, colored per genotype. (**c**) Median-joining network analysis of viruses belonging to genotypes H5N5-GT2. (**d**) Median-joining network analysis of viruses belonging to genotype H5N5-GT3. The number of nucleotide substitutions between strains are shown as values near branches. Detailed information on the virus sequences is provided in Table S2.

The collection locations of the European HPAI H5N5 viruses were plotted in a geographical map to elucidate the spatial distribution of the genotypes (Figure 2a). Genotype H5N5-GT1 was detected only once in the Netherlands. The collection locations of viruses belonging to genotype H5N5-GT3 were restricted to areas in the northern part of Germany and the Netherlands. In contrast, H5N5-GT2 viruses were isolated from six European countries (The Netherlands, Germany, Poland, Italy, Croatia, and Hungary), demonstrating that H5N5-GT2 was geographically the most widespread genotype.

Analysis of the collection dates shows that HPAI H5N5 viruses were detected between October 2016 and May 2017 (Figure 2b). Genotype H5N5-GT1 was isolated in the early phase of the outbreak (in November 2016), H5N5-GT2 during the outbreak peak (between December 2016 and March 2017), and H5N5-GT3 during the outbreak peak and in the late phase of the outbreak (in January, February and May 2017). This shows that the H5N5 genotypes were isolated in overlapping, but distinguishable outbreak phases.

### 3.3. Genetic Relationships between HPAI H5N5 Viruses

Phylogenetic network analysis was performed to study genetic relationships between the HPAI H5N5 viruses of the same genotype. For viruses belonging to genotypes H5N5-GT2 and H5N5-GT3, the full-length nucleotide consensus sequences of all eight gene segments were concatenated and median-joining networks were reconstructed. This analysis was not performed for genotype H5N5-GT1, as only one virus of this genotype was isolated. In addition, viruses with incomplete genome sequences (H5N5-5, H5N5-12, H5N5-20 and H5N5-21) were excluded.

The median-joining network of genotype H5N5-GT2 viruses shows that the Russian Kamchatka virus (H5N5-2) was genetically relatively more distantly related to the European strains (Figure 2c). The Croatian virus H5N5-10 was genetically most closely related, showing 55 nt differences. The viruses shared two nucleotide variants that were not present in other European strains. The two other viruses isolated in Croatia (H5N5-7 and -15) were more distantly related to the Russian Kamchatka virus (H5N5-2) (59–66 nt differences). Moreover, the three Croatian viruses (H5N5-7, -10 and -15) were genetically relatively distantly related to each other, showing 24–27 nt differences. In contrast, the H5N5 viruses isolated in the Netherlands (H5N5-4) and in Germany (H5N5-3, -6 and -11) were genetically closely related (10–18 nt differences), as they share a predicted common ancestor. The network further revealed clustering of the Italian strains (H5N5-8 and -9), showing 7 nt differences, suggesting local virus circulation.

Phylogenetic network analysis of genotype H5N5-GT3 viruses identified the German isolate H5N5-17 as a direct precursor of virus H5N5-18 (3 nt differences) that was isolated in the same region (Figure 2d). In contrast, the German isolate H5N5-16 and the Dutch isolate H5N5-19 share a predicted common ancestor but are genetically more distantly related (20–23 nt differences). Phylogenetic network analysis shows that genetic relationships between the H5N5 viruses are largely consistent with geographical location, and indicates multiple virus introductions into Europe followed by local spread.

### 3.4. Timing of the HPAI H5N5 Reassortment Events

To estimate the timing of the reassortment events that led to the emergence of the HPAI H5N5 genotypes, we performed molecular clock analysis. This analysis was not performed for genotype H5N5-GT1, which was detected only once. The median TMRCA for the individual gene segments of viruses belonging to genotypes H5N5-GT2 and H5N5-GT3 was estimated in the time-scaled phylogenetic trees (Table 2) (Figure S3). This molecular clock analysis showed that the European H5N5-GT2 viruses share a predicted common ancestor with the Russian Kamchatka virus, with a median TMRCA ranging from November 2015 (NA segment) to August 2016 (PA and MP segments) (nodes 1). These estimations of the TMRCA indicate that genotype H5N5-GT2 emerged in August 2016 (June–September 2016). The European H5N5-GT2 viruses share a predicted common ancestor with a median TMRCA ranging from April 2016 (NA segment) to November 2016 (MP and NS segments) (nodes 2). This analysis indicates that the European H5N5-GT2 viruses share a predicted common ancestor in November 2016 (November–December 2016).

Genotype H5N5-GT3 viruses share a predicted common ancestor with the Russian Kamchatka virus and the European H5N5-GT2 viruses for all gene segments except PB1 and NP, which were genetically most closely related to LPAI viruses detected in previous years. The median TMRCAs for the PB1 and NP gene segments of the H5N5-GT3 viruses were estimated in October 2016 (May 2016–January 2017) and September 2016 (March 2016–December 2016), respectively (nodes 3). These results suggest that the reassortment event that led to the emergence of genotype H5N5-GT3 occurred in the autumn of 2016.

The time-scaled phylogenetic trees further showed that although the HA, MP and NS genes of all three H5N5 genotypes fall in the same cluster of H5N8 China and Russia-Mongolia-like viruses, short phylogenetic distances within the clusters (so-called subclusters) were observed between genotype H5N5-GT1 and European H5N8 NL-Zeewolde-like viruses, and between genotypes H5N5-GT2 and H5N5-GT3. Interestingly, the MP gene of one H5N5-GT2 strain (H5N5-3) forms a phylogenetic subcluster with H5N8 viruses detected in Hungary, Germany and Poland in the same time period. These results suggest that this H5N5-GT2 virus may have obtained a novel MP gene by reassortment with H5N8 virus.

Altogether, molecular clock analysis shows variations in the timing of the reassortment events of the different H5N5 genotypes. The time at which the three genotypes emerged may have caused the differences in incidence and geographical distribution of the H5N5 viruses.

### 3.5. Cytopathogenicity and Replication of HPAI H5N5 Viruses in Primary Chicken and Duck Cells

**Figure 3:**
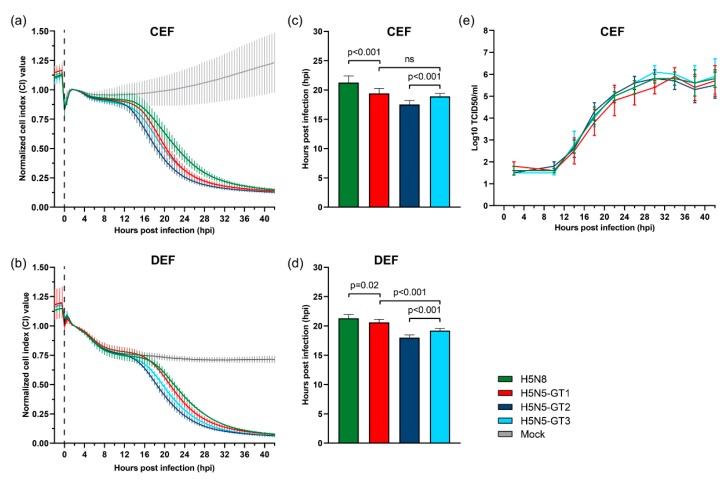
Cytopathogenicity and replication of HPAI H5N5 viruses in primary avian cells. (**a**,**b**) Cytopathogenicity of highly pathogenic avian influenza (HPAI) H5N5 and H5N8 virus in primary chicken embryo fibroblast (CEF) and duck embryo fibroblast (DEF) cells measured by the real-time cell analysis (RTCA) system. The electrical impedance of the cell-covered electrodes was displayed as cell index (CI) value and normalized at two hours post infection (hpi). Virus was inoculated at a multiplicity of infection (MOI) of 0.001. Mock-infected cells were taken along as negative controls (grey). (**c**,**d**) The mean time at which the CI value decreased to 50% of the maximum (CI50) value after infection of primary CEF and DEF cells with HPAI H5N5 and H5N8 virus. The p-value was calculated using a two-tailed unpaired Student’s t-test with *p* < 0.05 considered statistically significant. (**e**) Growth curves of HPAI H5N5 and H5N8 virus in primary CEF cells. Virus was inoculated at a MOI of 0.001. Samples were taken at four hour intervals from 2 to 42 hpi and titrated to determine the medium tissue culture infective dose (TCID50) titres. Error bars indicate standard deviations (SD).

We performed real-time cell analysis to determine the cytopathogenicity of HPAI H5N5 and H5N8 viruses in primary cultures of embryonic fibroblasts of chickens (CEF) and ducks (DEF). The cells were inoculated with viruses representing genotypes H5N5-GT1, H5N5-GT2, H5N5-GT3 and European H5N8 NL-Zeewolde-like virus (Table S4), and CPE was monitored by measuring the electrical impedance of the cell monolayer, which is expressed as the CI value. A decrease in CI value was observed between 10–12 hpi in inoculated CEF cells and between 12–14 hpi in inoculated DEF cells, marking the onset of virus-induced cytopathogenicity (Figure 3a,b). The CPE observed in the inoculated cultures increased over time, and resulted in complete cell death within 42 hours.

The time point at which half of the maximal CI value (CI50) was reached was determined to compare the cytopathogenicity of the different viruses. In CEF cells, the CI50 value was reached at 17.5 ± 0.7 hpi for H5N5-GT2 virus, at 18.9 ± 0.5 hpi for H5N5-GT3, and at 19.4 ± 0.8 hpi for H5N5-GT1 (Figure 3c). In DEF cells, cytopathogenicity for H5N5-GT2 was also highest (18.0 ± 0.5 hpi), followed by H5N5-GT3 (19.2 ± 0.4 hpi) and H5N5-GT1 (20.6 ± 0.5 hpi) (Figure 3d). Although the differences in cytopathogenicity between the viruses are small, these results suggest that genotype H5N5-GT2 is significantly more pathogenic to both primary chicken and duck cells compared to the other H5N5 genotypes (*p* < 0.001). In addition, H5N5-GT3 appeared more pathogenic to DEF cells than H5N5-GT-1 (*p* < 0.001). In contrast, upon inoculation with H5N8 virus, the CI50 value was reached at 21.3 ± 1.1 hpi in CEF cells and 21.3 ± 0.6 hpi in DEF cells, which was significantly slower compared to the H5N5 viruses (*p* < 0.001 and *p* < 0.05, respectively). This suggests that H5N8 is less pathogenic to primary chicken and duck cells compared to H5N5, although the measured differences are small.

In addition, we studied virus replication in CEF cells to investigate the relationship between cytopathogenicity and replication kinetics. Growth curves were generated by the collection of supernatants at four hour intervals (2–42 hpi), which were titrated to determine the infectious titres. Results show that H5N5 genotypes and H5N8 virus replicate to comparable virus titres in CEF cells (Figure 3e). The results suggest that the reassortment events may have resulted in minor changes in cytopathogenicity, whereas no changes in replication kinetics between H5N5 genotypes and H5N8 virus were observed in primary chicken cells.

## 4. Discussion

In 2016–2017, HPAI H5N8 clade 2.3.4.4 group B viruses caused a large-scale epizootic among wild birds and poultry in Europe. Concurrently, related HPAI viruses of subtype H5N5 were detected, although the number of birds detected with H5N5 infection was limited compared to H5N8. Genetic analysis demonstrates that three different genotypes of H5N5 were introduced into Europe. In this study, we analyzed the emergence, spread and in vitro characteristics of these genotypes.

Viruses of genotype H5N5-GT2 were most frequently isolated and geographically the most widespread in Europe. Phylogenetic analysis demonstrated that the European H5N5-GT2 viruses share a predicted common ancestor with the H5N5 virus isolated in the Kamchatka region of Russia at the beginning of October 2016. Most European H5N5-GT2 viruses were isolated in December 2016 and January 2017. Phylogenetic network analysis demonstrated genetic relationships between H5N5-GT2 viruses largely corresponding with collection locations, as the Russian Kamchatka virus was genetically distinct from the viruses isolated in Europe, and the H5N5 viruses detected in the Netherlands and Germany were genetically distinct from viruses isolated in south-eastern European countries (Poland, Czech Republic, Hungary, Croatia and Italy). The phylogenetic network further revealed a close genetic relationship between the Italian strains. The results therefore indicate multiple introductions of H5N5-GT2 viruses into Europe followed by local spread, similar to H5N8 viruses [46].

Most H5N5-GT2 viruses were detected during the peak of the HPAI H5N8 outbreak in Europe, when high mortality rates among wild birds and multiple outbreaks in commercial poultry were reported [46,47]. Molecular clock analysis indicated that the European H5N5-GT2 viruses share a predicted common ancestor in November 2016, which is in accordance with molecular clock analysis that was performed for Italian strains [22]. The European H5N5-GT2 viruses share a common ancestor with the Russian Kamchatka virus in August 2016, after European H5N8 viruses emerged between May–August [21,22,26]. Similar results were obtained in a previous study on the emergence of H5N5-GT2 [26]. These results suggest that genotype H5N5-GT2 presumably emerged in the summer of 2016 on the breeding grounds of migratory wild birds in the northern part of Russia. The emergence of the virus at the end of the breeding season, just before or after the start of autumn migration, may have resulted in limited spread compared to H5N8. The Kamchatka region is located in the Russian Far East, at a large distance from the known breeding sites for migratory wild birds in Russia. Therefore, the virus may have been dispersed from the common breeding areas to both Europe and the Kamchatka region during migration via separate flyways.

Phylogenetic analysis further indicated that, although belonging to genotype H5N5-GT2, one German virus (H5N5-3) obtained a novel MP gene by reassortment with H5N8 virus. This reassortment event resulted in a larger distance to other German H5N5-GT2 viruses (H5N5-6 and -11) and the Dutch H5N5-GT2 virus (H5N5-4). In the phylogenetic network, 6 out of 9 nucleotide differences between H5N5-3 and the predicted common ancestor of H5N5-3 and H5N5-11 were present in the MP gene.

The second most frequently detected HPAI H5N5 genotype is H5N5-GT3. Genetic analysis of the newly isolated H5N5 virus in the Netherlands revealed that this virus also belongs to genotype H5N5-GT3. The virus was isolated in May 2017, after a two-month gap of H5N5 detections in Europe. Phylogenetic analysis with other European H5N5 viruses showed that the virus shares a predicted common ancestor with viruses detected in Germany. Viruses with this gene constellation were solely detected in the northern part of Germany and in the Netherlands, indicating local virus circulation. The H5N5-GT3 viruses show a close genetic relationship with viruses of genotype H5N5-GT2, but contain reassorted PB1 and NP genes. These reassorted genes were genetically most closely related to LPAI viruses detected in the Netherlands in 2014, which may be explained by the intense wild bird surveillance activities in the Netherlands and the lack of recent sequence data. Molecular clock analysis estimated a common ancestor for the reassorted genes in September and October 2016. Possibly, an ancestor virus containing both gene segments has been circulating between 2014–2016 and was involved in the emergence of genotype H5N5-GT3 by reassortment with H5N5-GT2 virus during a single reassortment event. The timing of the reassortment event suggests that this occurred after the start of autumn migration, locally in Europe. No viruses of genotype H5N5-GT3 were detected on the breeding grounds, suggesting that relatively small amounts of wild birds became infected resulting in limited spread in Europe.

Genotype H5N5-GT1 was detected once, and this was the first detection of HPAI H5N5 virus in Europe. This detection was made in the Netherlands in November 2016, concurrently with the first cases of H5N8 in the Netherlands [21], and other European countries [16,48]. As previously reported, this genotype clustered phylogenetically with H5N8 viruses found in the Netherlands [21]. Therefore, H5N5-GT1 virus likely derived from reassortment of HPAI H5N8 and co-circulating LPAI viruses in the PA and NA genes [21,23,24]. As no viruses with the same gene constellation were detected and recent sequence data on genetically related LPAI viruses was missing, molecular clock analysis could not be used to estimate the timing of the reassortment event giving rise to this genotype. However, the single detection of H5N5-GT1 may suggest that the reassortment event occurred after wild birds migrated from their breeding grounds, which may have prevented the virus to spread among large populations of birds.

However, changes in virus characteristics, such as infectivity, transmissibility or pathogenicity may have also contributed to the limited spread of HPAI H5H5 viruses compared to H5N8 viruses. In this study, we infected primary chicken and duck cells to examine the cytopathogenicity and replication kinetics of the three H5N5 genotypes and H5N8 virus. For all tested viruses, infection of primary chicken and duck cells resulted in complete cell death within two days, demonstrating high cytopathogenicity. A comparison of the cytopathogenic effects revealed small differences between the three H5N5 genotypes and H5N8 virus in both primary chicken and duck cells. The H5N5 viruses appeared more cytopathogenic than the H5N8 virus, and cytopathogenicity of H5N5-GT2 was somewhat enhanced compared to H5N5-GT1 and H5N5-GT3. However, no changes in replication kinetics between the viruses were observed. The high in vitro cytopathogenicity and fast kinetics of virus replication are in accordance with the high IVPI scores that were reported for H5N5 viruses of genotypes H5N5-GT1 (3.00) ([21], unpublished results) and H5N5-GT2 (2.87–3.00) [22,24], which were comparable to H5N8 viruses (2.85–3.00) [13,22,23]. The H5N5 and H5N8 viruses carry the same HA, MP and NS gene segments, suggesting that the viral genetic factors associated with the high pathogenicity are likely present in these genes. The viruses contain the same HA cleavage site, which is the major determinant of the highly pathogenic phenotype. However, other genomic features may also contribute to the pathogenicity of the virus. A previous study identified truncations of the C-terminal of NS1 and the PB1-F2 protein, which are virulence factors associated with host adaptation [26]. These results indicate that the differences in the incidence and distribution between the viruses are no direct result from changes in pathogenicity or replication efficiency.

An important limitation of this study is that we used an in vitro system to investigate differences in pathogenicity and replication that will not fully represent the in vivo situation. The primary duck cells were obtained from Pekin ducks, a domestic duck breed derived from the mallard, while the viruses studied were isolated from a variety of wild bird species. In addition, pre-existing immunity in the wild bird population due to previous infections with related LPAI viruses may have protected wild birds against HPAI H5N5 infection, thereby influencing the spread of HPAI H5N5 viruses in Europe. In recent years, H5N2 virus descending from H5N8 group A viruses in 2014–2015 in North America [11], and H5N6 virus descending from H5N8 group B viruses in 2017–2018 in Europe [18], have dominated and even replaced co-circulating HPAI strains. However, the emergence of H5N5 virus from H5N8 group B viruses in 2016–2017 resulted in only limited infections. H5N2 viruses isolated during the outbreaks in North America in 2015 exhibited an unusual long pre-clinical period, long mean death time, and high level of viral shedding in turkeys, which may have contributed to the widespread distribution of H5N2 viruses [49]. Although H5N8 and H5N5 viruses both affected various poultry types, the high number of H5N8 outbreaks in poultry compared to H5N5 may have contributed to the increased dissemination of H5N8 viruses in Europe. Further experimental animal studies comparing H5N5 and H5N8 viruses should be performed to obtain insight in the infection dynamics of these viruses.

In conclusion, this study suggests that the limited spread and the differences in geographical distribution of HPAI H5N5 viruses are related to the timing of the reassortment events and introduction into Europe rather than changes in virus pathogenicity or replication kinetics.

## Figures and Tables

**Table 1:** HPAI H5N5 viruses. Highly pathogenic avian influenza (HPAI) H5N5 viruses isolated during the HPAI H5 2016–2017 epidemic, ordered by genotype. Detailed information on the virus sequences is provided in Table S2.

H5N5 Genotype	H5N5 Isolate Number	Host	Collection Date	Collection Location
**H5N5-GT1**	1	Tufted duck	2016-11-14	Netherlands
**H5N5-GT2**	2	Environment	2016-10-01	Russia
	3	Swan	2016-12-13	Germany
	4	Mute swan	2016-12-13	Netherlands
	5 ^a^	Barnacle goose	2016-12-22	Germany
	6	Greylag goose	2016-12-27	Germany
	7	Mute swan	2016-12-27	Croatia
	8	Eurasian wigeon	2016-12-29	Italy
	9	Gadwall	2017-01-10	Italy
	10	Mute swan	2017-01-20	Croatia
	11	Grey heron	2017-01-22	Germany
	12 ^a^	Mute swan	2017-01-31	Poland
	13	Common buzzard	2017-02-06	Germany
	14	Mute swan	2017-02-14	Hungary
	15	Chicken	2017-03-07	Croatia
**H5N5-GT3**	16	Turkey	2017-01-22	Germany
	17	Cormorant	2017-01-30	Germany
	18	Egret	2017-02-14	Germany
	19	Goose	2017-05-22	Netherlands
**Unknown**	20 ^a^	Mute swan	2017-02-09	Czech Republic
	21 ^a^	Spot-billed pelican	2017-02-14	Czech Republic

^a^ Viruses with incomplete genome sequence were excluded for phylogenetic network analysis.

**Table 2:** Time to the most recent common ancestor (TMRCA) estimates of HPAI H5N5 genotypes. Estimated median TMRCA for each gene segment of highly pathogenic avian influenza (HPAI) H5N5 viruses of genotype H5N5-GT2, the European H5N5-GT2 viruses and H5N5-GT3 viruses with 95% highest posterior density (HPD) intervals. The nodes for which the median TMRCAs estimates were determined are depicted in the time-scaled phylogenetic trees in Figure S3. PB2, polymerase basic protein 2; PB1, polymerase basic protein 1; PA, polymerase acidic protein; HA, hemagglutinin; NP, nucleoprotein; NA, neuraminidase; MP, matrix protein; NS, nonstructural protein.

	H5N5-GT2 Viruses (Node 1)				European H5N5-GT2 Viruses (Node 2)				H5N5-GT3 Viruses (Node 3)			
Gene Segment	Median TMRCA	Lower 95% HPD	Upper 95% HPD	Posterior	Median TMRCA	Lower 95% HPD	Upper 95% HPD	Posterior	Median TMRCA	Lower 95% HPD	Upper 95% HPD	Posterior
**PB2**	Mar-2016	Aug-2015	Aug-2016	0.9997	Sep-2016	May-2016	Nov-2016	0.9997	Oct-2016	Aug-2016	Nov-2016	0.4823
**PB1**	Dec-2015	Apr-2015	Jun-2016	0.9983	Jul-2016	Feb-2016	Oct-2016	0.9982	Oct-2016	May-2016	Jan-2017	0.9971
**PA**	Aug-2016	Jun-2016	Sep-2016	0.9982	Oct-2016	Sep-2016	Nov-2016	0.9995	Nov-2016	Oct-2016	Dec-2016	0.0942
**HA**	Jun-2016	Jan-2016	Aug-2016	0.9995	Oct-2016	Oct-2016	Jun-2016	0.9991	Dec-2016	Oct-2016	Jan-2017	0.9991
**NP**	Mar-2016	Aug-2015	Aug-2016	0.9988	Aug-2016	Apr-2016	Aug-2016	0.9988	Sep-2016	Mar-2016	Dec-2016	0.9991
**NA**	Nov-2015	Apr-2015	May-2016	0.9997	Apr-2016	Nov-2015	Aug-2016	0.9929	Oct-2016	Jun-2016	Jan-2017	0.9713
**MP**	Aug-2016	Jun-2016	Sep-2016	0.9929	Nov-2016	Nov-2016	Dec-2016	0.9928	Jan-2017	Dec-2016	Jan-2017	0.0325
**NS**	Jul-2016	Mar-2016	Sep-2016	0.9992	Nov-2016	Jun-2016	Aug-2016	0.9835	Dec-2016	Nov-2016	Dec-2016	0.0038

## Supplementary Materials

The following are available online at https://www.mdpi.com/1999-4915/11/6/501/s1, Table S1. Viruses most closely related to the newly isolated H5N5 strain. Table S2. Detailed information on H5N5 virus sequences. Table S3. List of H5N8 cluster representatives. Table S4. Viruses used for in vitro experiments. Table S5. Low pathogenic avian influenza (LPAI) ancestor viruses of reassortant genes. Figure S1. Phylogenetic analysis of H5N5 viruses. Figure S2. Gene constellations of H5N5 viruses. Figure S3. Maximum clade credibility trees.
