# Supplementary material for: Spread of Highly Pathogenic Avian Influenza (HPAI) H5N5 Viruses in Europe in 2016–2017 Appears Related to the Timing of Reassortment Events
Source: Viruses. 2019 May 31;11(6):501. doi: 10.3390/v11060501 (PMC6631432; doi:10.3390/v11060501)
Supplement: Supplementary file 1 [file viruses-11-00501-s001.zip › FigS1_revised.pdf]

Figure S1. Phylogenetic analysis of H5N5 viruses. Phylogenetic trees of individual gene segments of highly pathogenic avian influenza (HPAI) H5N5 viruses isolated during the HPAI H5 2016-2017 epizootic, including the top 20 sequence matches of other subtypes identified by BLAST in the GISAID's EpiFlu™ Database (http://www.gisaid.org) on 10 May 2019. The H5N5 virus sequences are coloured according to their genotype (H5N5-GT1, red; H5N5-GT2, dark blue; H5N5-GT3, light blue). As a reference, HPAI H5N8 cluster representatives were included (green). Detailed information on the HPAI H5N5 and H5N8 virus sequences is provided in Table S2 and Table S3, respectively. The most closely related low pathogenic avian influenza (LPAI) ancestor viruses of the reassortant genes, as listed in Table S5, are depicted in bold. Phylogenetic analysis was performed using the maximum likelihood (ML) method within the MEGA7 software package. PB2, polymerase basic 2; PB1, polymerase basic 1; PA, polymerase acidic; HA, hemagglutinin; NP, nucleoprotein; NA, neuraminidase; MP, matrix protein; NS, nonstructural protein.

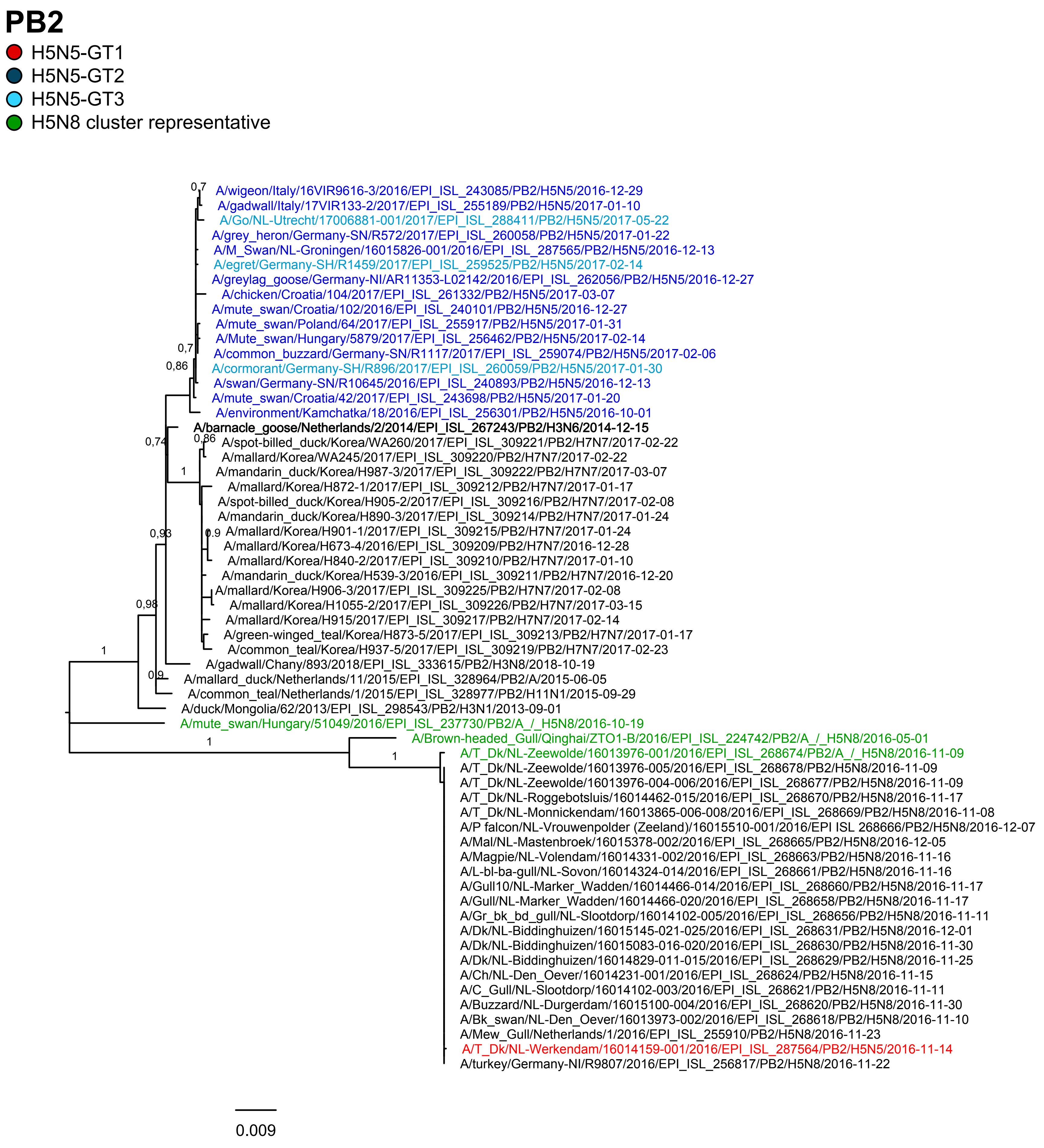
PB2
H5N5-GT1
H5N5-GT2
H5N5-GT3
H5N8 cluster representative
A/wigeon/Italy/16VIR9616-3/2016/EPI_ISL_243085/PB2/H5N5/2016-12-29
A/gadwall/Italy/17VIR133-2/2017/EPI_ISL_255189/PB2/H5N5/2017-01-10
A/Go/NL-Utrecht/17006881-001/2017/EPI_ISL_288411/PB2/H5N5/2017-05-22
A/grey_heron/Germany-SN/R572/2017/EPI_ISL_260058/PB2/H5N5/2017-01-22
A/M_Swan/NL-Groningen/16015826-001/2016/EPI_ISL_287565/PB2/H5N5/2016-12-13
A/egret/Germany-SH/R1459/2017/EPI_ISL_259525/PB2/H5N5/2017-02-14
A/greylag_goose/Germany-NI/AR11353-L02142/2016/EPI_ISL_262056/PB2/H5N5/2016-12-27
A/chicken/Croatia/104/2017/EPI_ISL_261332/PB2/H5N5/2017-03-07
A/mute_swan/Croatia/102/2016/EPI_ISL_240101/PB2/H5N5/2016-12-27
A/mute_swan/Poland/64/2017/EPI_ISL_255917/PB2/H5N5/2017-01-31
A/Mute_swan/Hungary/5879/2017/EPI_ISL_256462/PB2/H5N5/2017-02-14
A/common_buzzard/Germany-SN/R1117/2017/EPI_ISL_259074/PB2/H5N5/2017-02-06
A/cormorant/Germany-SH/R896/2017/EPI_ISL_260059/PB2/H5N5/2017-01-30
A/swan/Germany-SN/R10645/2016/EPI_ISL_240893/PB2/H5N5/2016-12-13
A/mute_swan/Croatia/42/2017/EPI_ISL_243698/PB2/H5N5/2017-01-20
A/environment/Kamchatka/18/2016/EPI_ISL_256301/PB2/H5N5/2016-10-01
A/barnacle_goose/Netherlands/2/2014/EPI_ISL_267243/PB2/H3N6/2014-12-15
A/spot-billed_duck/Korea/WA260/2017/EPI_ISL_309221/PB2/H7N7/2017-02-22
A/mallard/Korea/WA245/2017/EPI_ISL_309220/PB2/H7N7/2017-02-22
A/mandarin_duck/Korea/H987-3/2017/EPI_ISL_309222/PB2/H7N7/2017-03-07
A/mallard/Korea/H872-1/2017/EPI_ISL_309212/PB2/H7N7/2017-01-17
A/spot-billed_duck/Korea/H905-2/2017/EPI_ISL_309216/PB2/H7N7/2017-02-08
A/mandarin_duck/Korea/H890-3/2017/EPI_ISL_309214/PB2/H7N7/2017-01-24
A/mallard/Korea/H901-1/2017/EPI_ISL_309215/PB2/H7N7/2017-01-24
A/mallard/Korea/H673-4/2016/EPI_ISL_309209/PB2/H7N7/2016-12-28
A/mallard/Korea/H840-2/2017/EPI_ISL_309210/PB2/H7N7/2017-01-10
A/mandarin_duck/Korea/H539-3/2016/EPI_ISL_309211/PB2/H7N7/2016-12-20
A/mallard/Korea/H906-3/2017/EPI_ISL_309225/PB2/H7N7/2017-02-08
A/mallard/Korea/H1055-2/2017/EPI_ISL_309226/PB2/H7N7/2017-03-15
A/mallard/Korea/H915/2017/EPI_ISL_309217/PB2/H7N7/2017-02-14
A/green-winged_teal/Korea/H873-5/2017/EPI_ISL_309213/PB2/H7N7/2017-01-17
A/common_teal/Korea/H937-5/2017/EPI_ISL_309219/PB2/H7N7/2017-02-23
A/gadwall/Chany/893/2018/EPI_ISL_333615/PB2/H3N8/2018-10-19
A/mallard_duck/Netherlands/11/2015/EPI_ISL_328964/PB2/A/2015-06-05
A/common_teal/Netherlands/1/2015/EPI_ISL_328977/PB2/H11N1/2015-09-29
A/duck/Mongolia/62/2013/EPI_ISL_298543/PB2/H3N1/2013-09-01
A/mute_swan/Hungary/51049/2016/EPI_ISL_237730/PB2/A_/_H5N8/2016-10-19
A/Brown-headed_Gull/Qinghai/ZTO1-B/2016/EPI_ISL_224742/PB2/A_/_H5N8/2016-05-01
A/T_Dk/NL-Zeewolde/16013976-001/2016/EPI_ISL_268674/PB2/A_/_H5N8/2016-11-09
A/T_Dk/NL-Zeewolde/16013976-005/2016/EPI_ISL_268678/PB2/H5N8/2016-11-09
A/T_Dk/NL-Zeewolde/16013976-004-006/2016/EPI_ISL_268677/PB2/H5N8/2016-11-09
A/T_Dk/NL-Roggebotsluis/16014462-015/2016/EPI_ISL_268670/PB2/H5N8/2016-11-17
A/T_Dk/NL-Monnickendam/16013865-006-008/2016/EPI_ISL_268669/PB2/H5N8/2016-11-08
A/P falcon/NL-Vrouwenpolder (Zeeland)/16015510-001/2016/EPI ISL 268666/PB2/H5N8/2016-12-07
A/Mal/NL-Mastenbroek/16015378-002/2016/EPI_ISL_268665/PB2/H5N8/2016-12-05
A/Magpie/NL-Volendam/16014331-002/2016/EPI_ISL_268663/PB2/H5N8/2016-11-16
A/L-bl-ba-gull/NL-Sovon/16014324-014/2016/EPI_ISL_268661/PB2/H5N8/2016-11-16
A/Gull10/NL-Marker_Wadden/16014466-014/2016/EPI_ISL_268660/PB2/H5N8/2016-11-17
A/Gull/NL-Marker_Wadden/16014466-020/2016/EPI_ISL_268658/PB2/H5N8/2016-11-17
A/Gr_bk_bd_gull/NL-Slootdorp/16014102-005/2016/EPI_ISL_268656/PB2/H5N8/2016-11-11
A/Dk/NL-Biddinghuizen/16015145-021-025/2016/EPI_ISL_268631/PB2/H5N8/2016-12-01
A/Dk/NL-Biddinghuizen/16015083-016-020/2016/EPI_ISL_268630/PB2/H5N8/2016-11-30
A/Dk/NL-Biddinghuizen/16014829-011-015/2016/EPI_ISL_268629/PB2/H5N8/2016-11-25
A/Ch/NL-Den_Oever/16014231-001/2016/EPI_ISL_268624/PB2/H5N8/2016-11-15
A/C_Gull/NL-Slootdorp/16014102-003/2016/EPI_ISL_268621/PB2/H5N8/2016-11-11
A/Buzzard/NL-Durgerdam/16015100-004/2016/EPI_ISL_268620/PB2/H5N8/2016-11-30
A/Bk_swan/NL-Den_Oever/16013973-002/2016/EPI_ISL_268618/PB2/H5N8/2016-11-10
A/Mew_Gull/Netherlands/1/2016/EPI_ISL_255910/PB2/H5N8/2016-11-23
A/T_Dk/NL-Werkendam/16014159-001/2016/EPI_ISL_287564/PB2/H5N5/2016-11-14
A/turkey/Germany-NI/R9807/2016/EPI_ISL_256817/PB2/H5N8/2016-11-22
0,7
0,7
0,86
0,74
0,86
1
0,93
0.9
0,98
1
0,9
1
1
0.009

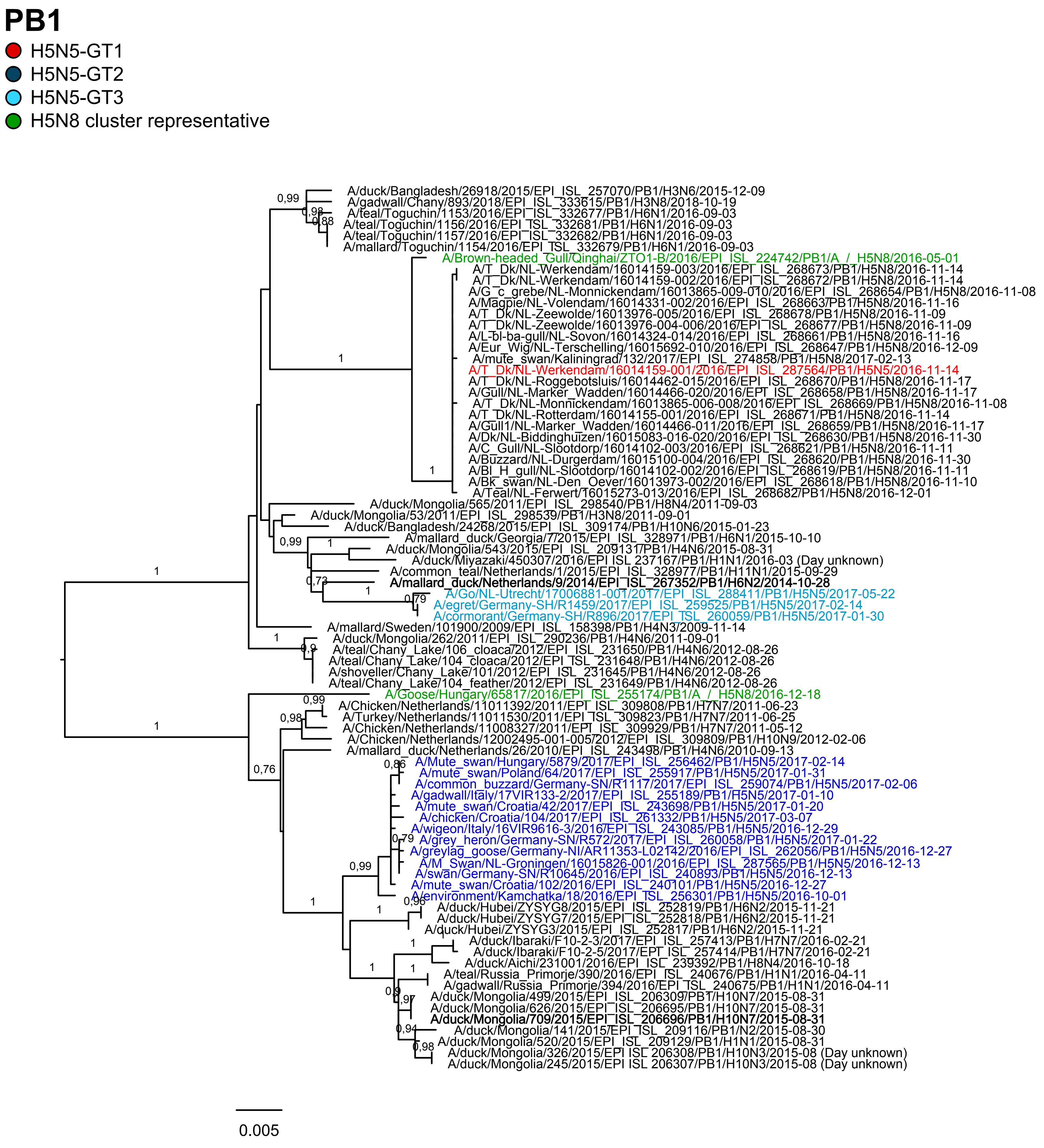
PB1
H5N5-GT1
H5N5-GT2
H5N5-GT3
H5N8 cluster representative
A/duck/Bangladesh/26918/2015/EPI_ISL_257070/PB1/H3N6/2015-12-09
A/gadwall/Chany/893/2018/EPI_ISL_333615/PB1/H3N8/2018-10-19
A/teal/Toguchin/1153/2016/EPI_ISL_332677/PB1/H6N1/2016-09-03
A/teal/Toguchin/1156/2016/EPI_ISL_332681/PB1/H6N1/2016-09-03
A/teal/Toguchin/1157/2016/EPI_ISL_332682/PB1/H6N1/2016-09-03
A/mallard/Toguchin/1154/2016/EPI_ISL_332679/PB1/H6N1/2016-09-03
A/Brown-headed_Gull/Qinghai/ZTO1-B/2016/EPI_ISL_224742/PB1/A_/_H5N8/2016-05-01
A/T_Dk/NL-Werkendam/16014159-003/2016/EPI_ISL_268673/PB1/H5N8/2016-11-14
A/T_Dk/NL-Werkendam/16014159-002/2016/EPI_ISL_268672/PB1/H5N8/2016-11-14
A/G_c_grebe/NL-Monnickendam/16013865-009-010/2016/EPI_ISL_268654/PB1/H5N8/2016-11-08
A/Magpie/NL-Volendam/16014331-002/2016/EPI_ISL_268663/PB1/H5N8/2016-11-16
A/T_Dk/NL-Zeewolde/16013976-005/2016/EPI_ISL_268678/PB1/H5N8/2016-11-09
A/T_Dk/NL-Zeewolde/16013976-004-006/2016/EPI_ISL_268677/PB1/H5N8/2016-11-09
A/L-bl-ba-gull/NL-Sovon/16014324-014/2016/EPI_ISL_268661/PB1/H5N8/2016-11-16
A/Eur_Wig/NL-Terschelling/16015692-010/2016/EPI_ISL_268647/PB1/H5N8/2016-12-09
A/mute_swan/Kaliningrad/132/2017/EPI_ISL_274858/PB1/H5N8/2017-02-13
A/T_Dk/NL-Werkendam/16014159-001/2016/EPI_ISL_287564/PB1/H5N5/2016-11-14
A/T_Dk/NL-Roggebotsluis/16014462-015/2016/EPI_ISL_268670/PB1/H5N8/2016-11-17
A/Gull/NL-Marker_Wadden/16014466-020/2016/EPI_ISL_268658/PB1/H5N8/2016-11-17
A/T_Dk/NL-Monnickendam/16013865-006-008/2016/EPI_ISL_268669/PB1/H5N8/2016-11-08
A/T_Dk/NL-Rotterdam/16014155-001/2016/EPI_ISL_268671/PB1/H5N8/2016-11-14
A/Gull1/NL-Marker_Wadden/16014466-011/2016/EPI_ISL_268659/PB1/H5N8/2016-11-17
A/Dk/NL-Biddinghuizen/16015083-016-020/2016/EPI_ISL_268630/PB1/H5N8/2016-11-30
A/C_Gull/NL-Slootdorp/16014102-003/2016/EPI_ISL_268621/PB1/H5N8/2016-11-11
A/Buzzard/NL-Durgerdam/16015100-004/2016/EPI_ISL_268620/PB1/H5N8/2016-11-30
A/Bl_H_gull/NL-Slootdorp/16014102-002/2016/EPI_ISL_268619/PB1/H5N8/2016-11-11
A/Bk_swan/NL-Den_Oever/16013973-002/2016/EPI_ISL_268618/PB1/H5N8/2016-11-10
A/Teal/NL-Ferwert/16015273-013/2016/EPI_ISL_268682/PB1/H5N8/2016-12-01
A/duck/Mongolia/565/2011/EPI_ISL_298540/PB1/H8N4/2011-09-03
A/duck/Mongolia/53/2011/EPI_ISL_298539/PB1/H3N8/2011-09-01
A/duck/Bangladesh/24268/2015/EPI_ISL_309174/PB1/H10N6/2015-01-23
A/mallard_duck/Georgia/7/2015/EPI_ISL_328971/PB1/H6N1/2015-10-10
A/duck/Mongolia/543/2015/EPI_ISL_209131/PB1/H4N6/2015-08-31
A/duck/Miyazaki/450307/2016/EPI ISL 237167/PB1/H1N1/2016-03 (Day unknown)
A/common_teal/Netherlands/1/2015/EPI_ISL_328977/PB1/H11N1/2015-09-29
A/mallard_duck/Netherlands/9/2014/EPI_ISL_267352/PB1/H6N2/2014-10-28
A/Go/NL-Utrecht/17006881-001/2017/EPI_ISL_288411/PB1/H5N5/2017-05-22
A/egret/Germany-SH/R1459/2017/EPI_ISL_259525/PB1/H5N5/2017-02-14
A/cormorant/Germany-SH/R896/2017/EPI_ISL_260059/PB1/H5N5/2017-01-30
A/mallard/Sweden/101900/2009/EPI_ISL_158398/PB1/H4N3/2009-11-14
A/duck/Mongolia/262/2011/EPI_ISL_290236/PB1/H4N6/2011-09-01
A/teal/Chany_Lake/106_cloaca/2012/EPI_ISL_231650/PB1/H4N6/2012-08-26
A/teal/Chany_Lake/104_cloaca/2012/EPI_ISL_231648/PB1/H4N6/2012-08-26
A/shoveller/Chany_Lake/101/2012/EPI_ISL_231645/PB1/H4N6/2012-08-26
A/teal/Chany_Lake/104_feather/2012/EPI_ISL_231649/PB1/H4N6/2012-08-26
A/Goose/Hungary/65817/2016/EPI_ISL_255174/PB1/A_/_H5N8/2016-12-18
A/Chicken/Netherlands/11011392/2011/EPI_ISL_309808/PB1/H7N7/2011-06-23
A/Turkey/Netherlands/11011530/2011/EPI_ISL_309823/PB1/H7N7/2011-06-25
A/Chicken/Netherlands/11008327/2011/EPI_ISL_309929/PB1/H7N7/2011-05-12
A/Chicken/Netherlands/12002495-001-005/2012/EPI_ISL_309809/PB1/H10N9/2012-02-06
A/mallard_duck/Netherlands/26/2010/EPI_ISL_243498/PB1/H4N6/2010-09-13
A/Mute_swan/Hungary/5879/2017/EPI_ISL_256462/PB1/H5N5/2017-02-14
A/mute_swan/Poland/64/2017/EPI_ISL_255917/PB1/H5N5/2017-01-31
A/common_buzzard/Germany-SN/R1117/2017/EPI_ISL_259074/PB1/H5N5/2017-02-06
A/gadwall/Italy/17VIR133-2/2017/EPI_ISL_255189/PB1/H5N5/2017-01-10
A/mute_swan/Croatia/42/2017/EPI_ISL_243698/PB1/H5N5/2017-01-20
A/chicken/Croatia/104/2017/EPI_ISL_261332/PB1/H5N5/2017-03-07
A/wigeon/Italy/16VIR9616-3/2016/EPI_ISL_243085/PB1/H5N5/2016-12-29
A/grey_heron/Germany-SN/R572/2017/EPI_ISL_260058/PB1/H5N5/2017-01-22
A/greylag_goose/Germany-NI/AR11353-L02142/2016/EPI_ISL_262056/PB1/H5N5/2016-12-27
A/M_Swan/NL-Groningen/16015826-001/2016/EPI_ISL_287565/PB1/H5N5/2016-12-13
A/swan/Germany-SN/R10645/2016/EPI_ISL_240893/PB1/H5N5/2016-12-13
A/mute_swan/Croatia/102/2016/EPI_ISL_240101/PB1/H5N5/2016-12-27
A/environment/Kamchatka/18/2016/EPI_ISL_256301/PB1/H5N5/2016-10-01
A/duck/Hubei/ZYSYG8/2015/EPI_ISL_252819/PB1/H6N2/2015-11-21
A/duck/Hubei/ZYSYG7/2015/EPI_ISL_252818/PB1/H6N2/2015-11-21
A/duck/Hubei/ZYSYG3/2015/EPI_ISL_252817/PB1/H6N2/2015-11-21
A/duck/Ibaraki/F10-2-3/2017/EPI_ISL_257413/PB1/H7N7/2016-02-21
A/duck/Ibaraki/F10-2-5/2017/EPI_ISL_257414/PB1/H7N7/2016-02-21
A/duck/Aichi/231001/2016/EPI_ISL_239392/PB1/H8N4/2016-10-18
A/teal/Russia_Primorje/390/2016/EPI_ISL_240676/PB1/H1N1/2016-04-11
A/gadwall/Russia_Primorje/394/2016/EPI_ISL_240675/PB1/H1N1/2016-04-11
A/duck/Mongolia/499/2015/EPI_ISL_206309/PB1/H10N7/2015-08-31
A/duck/Mongolia/626/2015/EPI_ISL_206695/PB1/H10N7/2015-08-31
A/duck/Mongolia/709/2015/EPI_ISL_206696/PB1/H10N7/2015-08-31
A/duck/Mongolia/141/2015/EPI_ISL_209116/PB1/N2/2015-08-30
A/duck/Mongolia/520/2015/EPI_ISL_209129/PB1/H1N1/2015-08-31
A/duck/Mongolia/326/2015/EPI ISL 206308/PB1/H10N3/2015-08 (Day unknown)
A/duck/Mongolia/245/2015/EPI ISL 206307/PB1/H10N3/2015-08 (Day unknown)
0.005

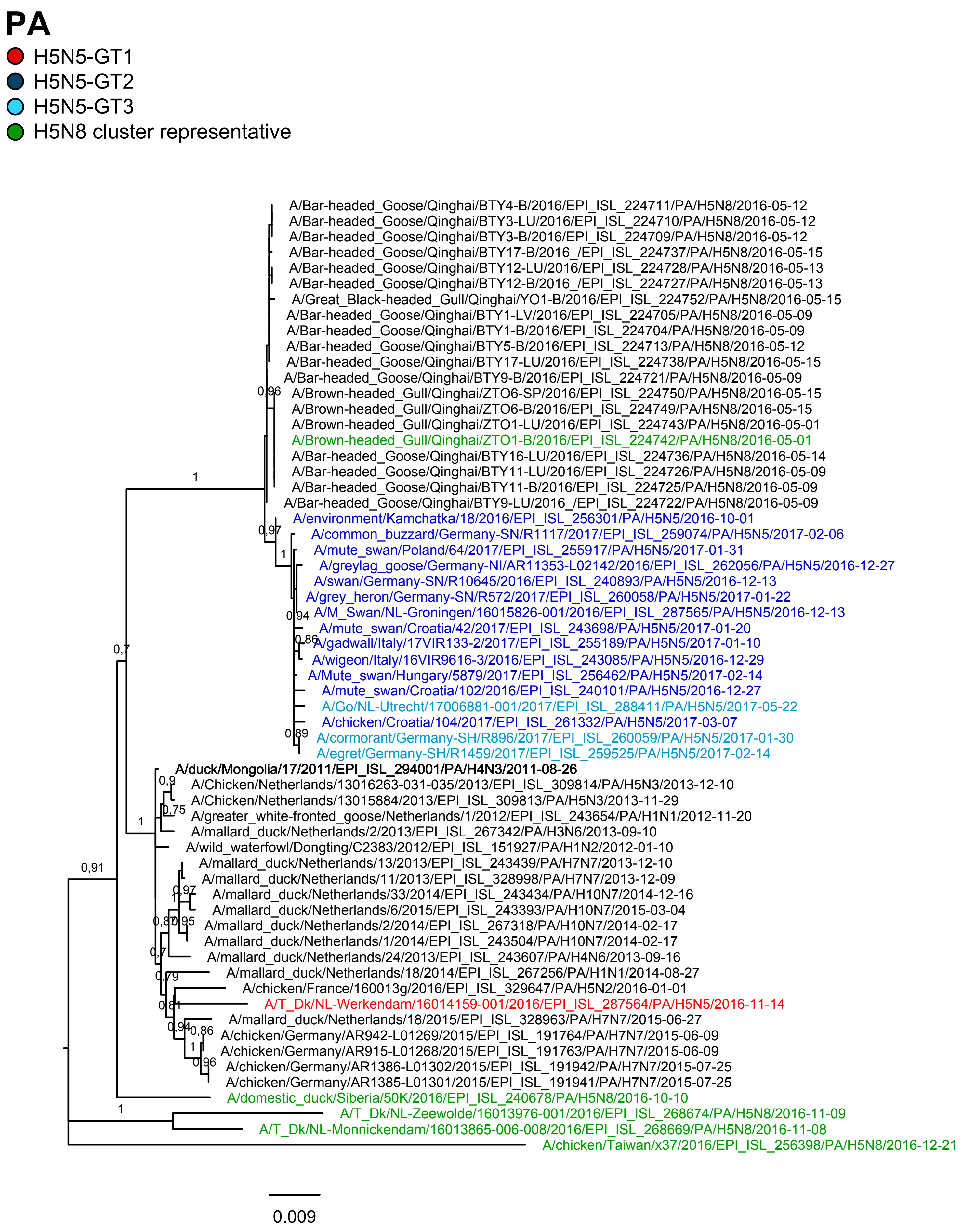
PA
H5N5-GT1
H5N5-GT2
H5N5-GT3
H5N8 cluster representative
A/Bar-headed_Goose/Qinghai/BTY4-B/2016/EPI_ISL_224711/PA/H5N8/2016-05-12
A/Bar-headed_Goose/Qinghai/BTY3-LU/2016/EPI_ISL_224710/PA/H5N8/2016-05-12
A/Bar-headed_Goose/Qinghai/BTY3-B/2016/EPI_ISL_224709/PA/H5N8/2016-05-12
A/Bar-headed_Goose/Qinghai/BTY17-B/2016_/EPI_ISL_224737/PA/H5N8/2016-05-15
A/Bar-headed_Goose/Qinghai/BTY12-LU/2016/EPI_ISL_224728/PA/H5N8/2016-05-13
A/Bar-headed_Goose/Qinghai/BTY12-B/2016_/EPI_ISL_224727/PA/H5N8/2016-05-13
A/Great_Black-headed_Gull/Qinghai/YO1-B/2016/EPI_ISL_224752/PA/H5N8/2016-05-15
A/Bar-headed_Goose/Qinghai/BTY1-LV/2016/EPI_ISL_224705/PA/H5N8/2016-05-09
A/Bar-headed_Goose/Qinghai/BTY1-B/2016/EPI_ISL_224704/PA/H5N8/2016-05-09
A/Bar-headed_Goose/Qinghai/BTY5-B/2016/EPI_ISL_224713/PA/H5N8/2016-05-12
A/Bar-headed_Goose/Qinghai/BTY17-LU/2016/EPI_ISL_224738/PA/H5N8/2016-05-15
A/Bar-headed_Goose/Qinghai/BTY9-B/2016/EPI_ISL_224721/PA/H5N8/2016-05-09
0,96
A/Brown-headed_Gull/Qinghai/ZTO6-SP/2016/EPI_ISL_224750/PA/H5N8/2016-05-15
A/Brown-headed_Gull/Qinghai/ZTO6-B/2016/EPI_ISL_224749/PA/H5N8/2016-05-15
A/Brown-headed_Gull/Qinghai/ZTO1-LU/2016/EPI_ISL_224743/PA/H5N8/2016-05-01
A/Brown-headed_Gull/Qinghai/ZTO1-B/2016/EPI_ISL_224742/PA/H5N8/2016-05-01
A/Bar-headed_Goose/Qinghai/BTY16-LU/2016/EPI_ISL_224736/PA/H5N8/2016-05-14
A/Bar-headed_Goose/Qinghai/BTY11-LU/2016/EPI_ISL_224726/PA/H5N8/2016-05-09
1
A/Bar-headed_Goose/Qinghai/BTY11-B/2016/EPI_ISL_224725/PA/H5N8/2016-05-09
A/Bar-headed_Goose/Qinghai/BTY9-LU/2016_/EPI_ISL_224722/PA/H5N8/2016-05-09
A/environment/Kamchatka/18/2016/EPI_ISL_256301/PA/H5N5/2016-10-01
0,97
A/common_buzzard/Germany-SN/R1117/2017/EPI_ISL_259074/PA/H5N5/2017-02-06
1
A/mute_swan/Poland/64/2017/EPI_ISL_255917/PA/H5N5/2017-01-31
A/greylag_goose/Germany-NI/AR11353-L02142/2016/EPI_ISL_262056/PA/H5N5/2016-12-27
A/swan/Germany-SN/R10645/2016/EPI_ISL_240893/PA/H5N5/2016-12-13
A/grey_heron/Germany-SN/R572/2017/EPI_ISL_260058/PA/H5N5/2017-01-22
0,94
A/M_Swan/NL-Groningen/16015826-001/2016/EPI_ISL_287565/PA/H5N5/2016-12-13
A/mute_swan/Croatia/42/2017/EPI_ISL_243698/PA/H5N5/2017-01-20
0,86
A/gadwall/Italy/17VIR133-2/2017/EPI_ISL_255189/PA/H5N5/2017-01-10
0,7
A/wigeon/Italy/16VIR9616-3/2016/EPI_ISL_243085/PA/H5N5/2016-12-29
A/Mute_swan/Hungary/5879/2017/EPI_ISL_256462/PA/H5N5/2017-02-14
A/mute_swan/Croatia/102/2016/EPI_ISL_240101/PA/H5N5/2016-12-27
A/Go/NL-Utrecht/17006881-001/2017/EPI_ISL_288411/PA/H5N5/2017-05-22
A/chicken/Croatia/104/2017/EPI_ISL_261332/PA/H5N5/2017-03-07
0,89
A/cormorant/Germany-SH/R896/2017/EPI_ISL_260059/PA/H5N5/2017-01-30
A/egret/Germany-SH/R1459/2017/EPI_ISL_259525/PA/H5N5/2017-02-14
A/duck/Mongolia/17/2011/EPI_ISL_294001/PA/H4N3/2011-08-26
0,9
A/Chicken/Netherlands/13016263-031-035/2013/EPI_ISL_309814/PA/H5N3/2013-12-10
A/Chicken/Netherlands/13015884/2013/EPI_ISL_309813/PA/H5N3/2013-11-29
0,75
A/greater_white-fronted_goose/Netherlands/1/2012/EPI_ISL_243654/PA/H1N1/2012-11-20
1
A/mallard_duck/Netherlands/2/2013/EPI_ISL_267342/PA/H3N6/2013-09-10
A/wild_waterfowl/Dongting/C2383/2012/EPI_ISL_151927/PA/H1N2/2012-01-10
0,91
A/mallard_duck/Netherlands/13/2013/EPI_ISL_243439/PA/H7N7/2013-12-10
A/mallard_duck/Netherlands/11/2013/EPI_ISL_328998/PA/H7N7/2013-12-09
0,97
1
A/mallard_duck/Netherlands/33/2014/EPI_ISL_243434/PA/H10N7/2014-12-16
A/mallard_duck/Netherlands/6/2015/EPI_ISL_243393/PA/H10N7/2015-03-04
0,87
0,95
A/mallard_duck/Netherlands/2/2014/EPI_ISL_267318/PA/H10N7/2014-02-17
A/mallard_duck/Netherlands/1/2014/EPI_ISL_243504/PA/H10N7/2014-02-17
0,7
A/mallard_duck/Netherlands/24/2013/EPI_ISL_243607/PA/H4N6/2013-09-16
0,79
A/mallard_duck/Netherlands/18/2014/EPI_ISL_267256/PA/H1N1/2014-08-27
A/chicken/France/160013g/2016/EPI_ISL_329647/PA/H5N2/2016-01-01
0,81
A/T_Dk/NL-Werkendam/16014159-001/2016/EPI_ISL_287564/PA/H5N5/2016-11-14
A/mallard_duck/Netherlands/18/2015/EPI_ISL_328963/PA/H7N7/2015-06-27
0,94
0,86
A/chicken/Germany/AR942-L01269/2015/EPI_ISL_191764/PA/H7N7/2015-06-09
1
A/chicken/Germany/AR915-L01268/2015/EPI_ISL_191763/PA/H7N7/2015-06-09
0,96
A/chicken/Germany/AR1386-L01302/2015/EPI_ISL_191942/PA/H7N7/2015-07-25
A/chicken/Germany/AR1385-L01301/2015/EPI_ISL_191941/PA/H7N7/2015-07-25
A/domestic_duck/Siberia/50K/2016/EPI_ISL_240678/PA/H5N8/2016-10-10
1
A/T_Dk/NL-Zeewolde/16013976-001/2016/EPI_ISL_268674/PA/H5N8/2016-11-09
A/T_Dk/NL-Monnickendam/16013865-006-008/2016/EPI_ISL_268669/PA/H5N8/2016-11-08
A/chicken/Taiwan/x37/2016/EPI_ISL_256398/PA/H5N8/2016-12-21
0.009

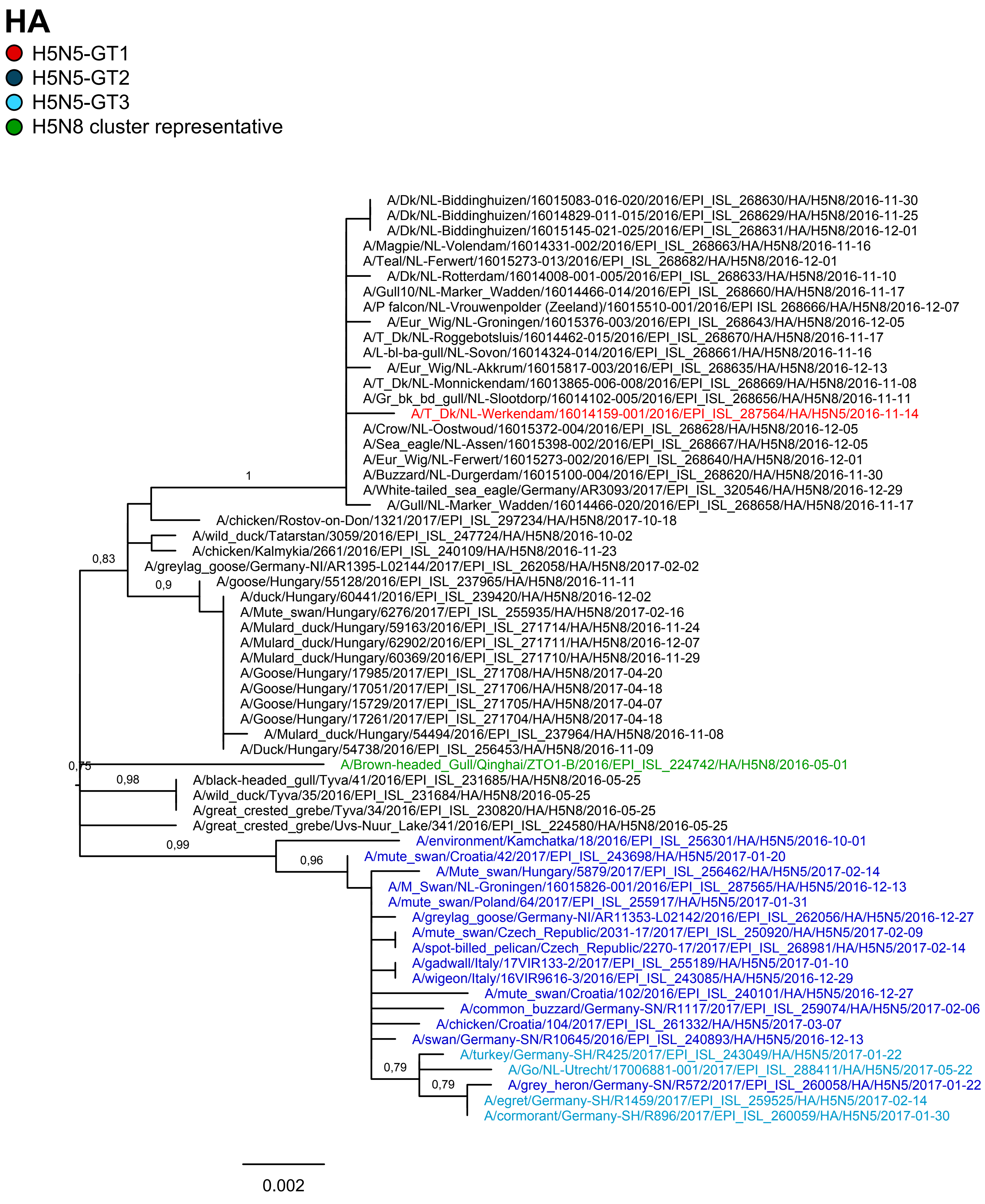
HA
H5N5-GT1
H5N5-GT2
H5N5-GT3
H5N8 cluster representative
A/Dk/NL-Biddinghuizen/16015083-016-020/2016/EPI_ISL_268630/HA/H5N8/2016-11-30
A/Dk/NL-Biddinghuizen/16014829-011-015/2016/EPI_ISL_268629/HA/H5N8/2016-11-25
A/Dk/NL-Biddinghuizen/16015145-021-025/2016/EPI_ISL_268631/HA/H5N8/2016-12-01
A/Magpie/NL-Volendam/16014331-002/2016/EPI_ISL_268663/HA/H5N8/2016-11-16
A/Teal/NL-Ferwert/16015273-013/2016/EPI_ISL_268682/HA/H5N8/2016-12-01
A/Dk/NL-Rotterdam/16014008-001-005/2016/EPI_ISL_268633/HA/H5N8/2016-11-10
A/Gull10/NL-Marker_Wadden/16014466-014/2016/EPI_ISL_268660/HA/H5N8/2016-11-17
A/P falcon/NL-Vrouwenpolder (Zeeland)/16015510-001/2016/EPI ISL 268666/HA/H5N8/2016-12-07
A/Eur_Wig/NL-Groningen/16015376-003/2016/EPI_ISL_268643/HA/H5N8/2016-12-05
A/T_Dk/NL-Roggebotsluis/16014462-015/2016/EPI_ISL_268670/HA/H5N8/2016-11-17
A/L-bl-ba-gull/NL-Sovon/16014324-014/2016/EPI_ISL_268661/HA/H5N8/2016-11-16
A/Eur_Wig/NL-Akkrum/16015817-003/2016/EPI_ISL_268635/HA/H5N8/2016-12-13
A/T_Dk/NL-Monnickendam/16013865-006-008/2016/EPI_ISL_268669/HA/H5N8/2016-11-08
A/Gr_bk_bd_gull/NL-Slootdorp/16014102-005/2016/EPI_ISL_268656/HA/H5N8/2016-11-11
A/T_Dk/NL-Werkendam/16014159-001/2016/EPI_ISL_287564/HA/H5N5/2016-11-14
A/Crow/NL-Oostwoud/16015372-004/2016/EPI_ISL_268628/HA/H5N8/2016-12-05
A/Sea_eagle/NL-Assen/16015398-002/2016/EPI_ISL_268667/HA/H5N8/2016-12-05
A/Eur_Wig/NL-Ferwert/16015273-002/2016/EPI_ISL_268640/HA/H5N8/2016-12-01
1
A/Buzzard/NL-Durgerdam/16015100-004/2016/EPI_ISL_268620/HA/H5N8/2016-11-30
A/White-tailed_sea_eagle/Germany/AR3093/2017/EPI_ISL_320546/HA/H5N8/2016-12-29
A/Gull/NL-Marker_Wadden/16014466-020/2016/EPI_ISL_268658/HA/H5N8/2016-11-17
A/chicken/Rostov-on-Don/1321/2017/EPI_ISL_297234/HA/H5N8/2017-10-18
A/wild_duck/Tatarstan/3059/2016/EPI_ISL_247724/HA/H5N8/2016-10-02
A/chicken/Kalmykia/2661/2016/EPI_ISL_240109/HA/H5N8/2016-11-23
0,83
A/greylag_goose/Germany-NI/AR1395-L02144/2017/EPI_ISL_262058/HA/H5N8/2017-02-02
0,9
A/goose/Hungary/55128/2016/EPI_ISL_237965/HA/H5N8/2016-11-11
A/duck/Hungary/60441/2016/EPI_ISL_239420/HA/H5N8/2016-12-02
A/Mute_swan/Hungary/6276/2017/EPI_ISL_255935/HA/H5N8/2017-02-16
A/Mulard_duck/Hungary/59163/2016/EPI_ISL_271714/HA/H5N8/2016-11-24
A/Mulard_duck/Hungary/62902/2016/EPI_ISL_271711/HA/H5N8/2016-12-07
A/Mulard_duck/Hungary/60369/2016/EPI_ISL_271710/HA/H5N8/2016-11-29
A/Goose/Hungary/17985/2017/EPI_ISL_271708/HA/H5N8/2017-04-20
A/Goose/Hungary/17051/2017/EPI_ISL_271706/HA/H5N8/2017-04-18
A/Goose/Hungary/15729/2017/EPI_ISL_271705/HA/H5N8/2017-04-07
A/Goose/Hungary/17261/2017/EPI_ISL_271704/HA/H5N8/2017-04-18
A/Mulard_duck/Hungary/54494/2016/EPI_ISL_237964/HA/H5N8/2016-11-08
A/Duck/Hungary/54738/2016/EPI_ISL_256453/HA/H5N8/2016-11-09
0,75
A/Brown-headed_Gull/Qinghai/ZTO1-B/2016/EPI_ISL_224742/HA/H5N8/2016-05-01
0,98
A/black-headed_gull/Tyva/41/2016/EPI_ISL_231685/HA/H5N8/2016-05-25
A/wild_duck/Tyva/35/2016/EPI_ISL_231684/HA/H5N8/2016-05-25
A/great_crested_grebe/Tyva/34/2016/EPI_ISL_230820/HA/H5N8/2016-05-25
A/great_crested_grebe/Uvs-Nuur_Lake/341/2016/EPI_ISL_224580/HA/H5N8/2016-05-25
0,99
A/environment/Kamchatka/18/2016/EPI_ISL_256301/HA/H5N5/2016-10-01
0,96
A/mute_swan/Croatia/42/2017/EPI_ISL_243698/HA/H5N5/2017-01-20
A/Mute_swan/Hungary/5879/2017/EPI_ISL_256462/HA/H5N5/2017-02-14
A/M_Swan/NL-Groningen/16015826-001/2016/EPI_ISL_287565/HA/H5N5/2016-12-13
A/mute_swan/Poland/64/2017/EPI_ISL_255917/HA/H5N5/2017-01-31
A/greylag_goose/Germany-NI/AR11353-L02142/2016/EPI_ISL_262056/HA/H5N5/2016-12-27
A/mute_swan/Czech_Republic/2031-17/2017/EPI_ISL_250920/HA/H5N5/2017-02-09
A/spot-billed_pelican/Czech_Republic/2270-17/2017/EPI_ISL_268981/HA/H5N5/2017-02-14
A/gadwall/Italy/17VIR133-2/2017/EPI_ISL_255189/HA/H5N5/2017-01-10
A/wigeon/Italy/16VIR9616-3/2016/EPI_ISL_243085/HA/H5N5/2016-12-29
A/mute_swan/Croatia/102/2016/EPI_ISL_240101/HA/H5N5/2016-12-27
A/common_buzzard/Germany-SN/R1117/2017/EPI_ISL_259074/HA/H5N5/2017-02-06
A/chicken/Croatia/104/2017/EPI_ISL_261332/HA/H5N5/2017-03-07
A/swan/Germany-SN/R10645/2016/EPI_ISL_240893/HA/H5N5/2016-12-13
A/turkey/Germany-SH/R425/2017/EPI_ISL_243049/HA/H5N5/2017-01-22
0,79
A/Go/NL-Utrecht/17006881-001/2017/EPI_ISL_288411/HA/H5N5/2017-05-22
0,79
A/grey_heron/Germany-SN/R572/2017/EPI_ISL_260058/HA/H5N5/2017-01-22
A/egret/Germany-SH/R1459/2017/EPI_ISL_259525/HA/H5N5/2017-02-14
A/cormorant/Germany-SH/R896/2017/EPI_ISL_260059/HA/H5N5/2017-01-30
0.002

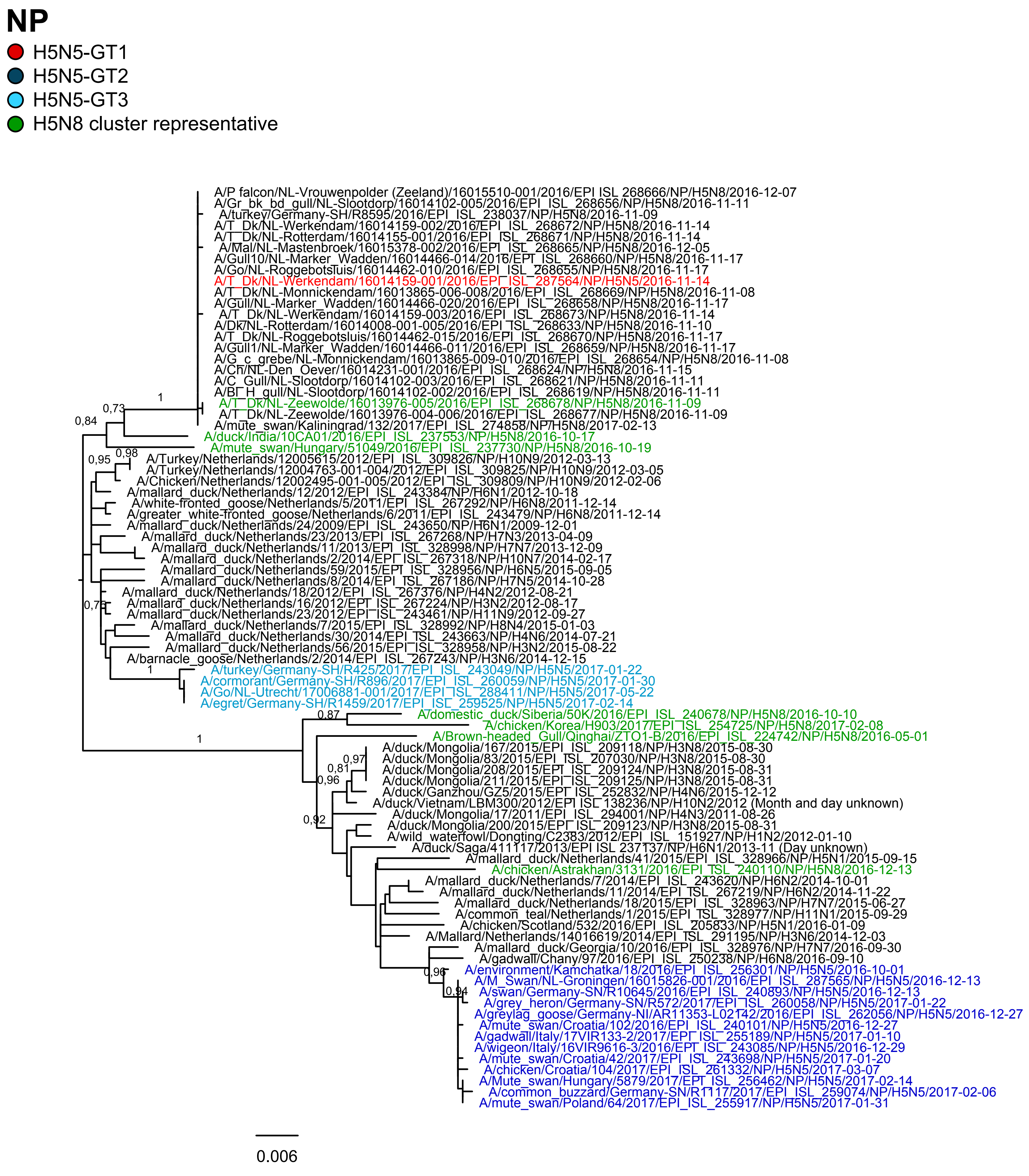
NP
H5N5-GT1
H5N5-GT2
H5N5-GT3
H5N8 cluster representative
A/P falcon/NL-Vrouwenpolder (Zeeland)/16015510-001/2016/EPI ISL 268666/NP/H5N8/2016-12-07
A/Gr_bk_bd_gull/NL-Slootdorp/16014102-005/2016/EPI_ISL_268656/NP/H5N8/2016-11-11
A/turkey/Germany-SH/R8595/2016/EPI_ISL_238037/NP/H5N8/2016-11-09
A/T_Dk/NL-Werkendam/16014159-002/2016/EPI_ISL_268672/NP/H5N8/2016-11-14
A/T_Dk/NL-Rotterdam/16014155-001/2016/EPI_ISL_268671/NP/H5N8/2016-11-14
A/Mal/NL-Mastenbroek/16015378-002/2016/EPI_ISL_268665/NP/H5N8/2016-12-05
A/Gull10/NL-Marker_Wadden/16014466-014/2016/EPI_ISL_268660/NP/H5N8/2016-11-17
A/Go/NL-Roggebotsluis/16014462-010/2016/EPI_ISL_268655/NP/H5N8/2016-11-17
A/T_Dk/NL-Werkendam/16014159-001/2016/EPI_ISL_287564/NP/H5N5/2016-11-14
A/T_Dk/NL-Monnickendam/16013865-006-008/2016/EPI_ISL_268669/NP/H5N8/2016-11-08
A/Gull/NL-Marker_Wadden/16014466-020/2016/EPI_ISL_268658/NP/H5N8/2016-11-17
A/T_Dk/NL-Werkendam/16014159-003/2016/EPI_ISL_268673/NP/H5N8/2016-11-14
A/Dk/NL-Rotterdam/16014008-001-005/2016/EPI_ISL_268633/NP/H5N8/2016-11-10
A/T_Dk/NL-Roggebotsluis/16014462-015/2016/EPI_ISL_268670/NP/H5N8/2016-11-17
A/Gull1/NL-Marker_Wadden/16014466-011/2016/EPI_ISL_268659/NP/H5N8/2016-11-17
A/G_c_grebe/NL-Monnickendam/16013865-009-010/2016/EPI_ISL_268654/NP/H5N8/2016-11-08
A/Ch/NL-Den_Oever/16014231-001/2016/EPI_ISL_268624/NP/H5N8/2016-11-15
A/C_Gull/NL-Slootdorp/16014102-003/2016/EPI_ISL_268621/NP/H5N8/2016-11-11
A/Bl_H_gull/NL-Slootdorp/16014102-002/2016/EPI_ISL_268619/NP/H5N8/2016-11-11
A/T_Dk/NL-Zeewolde/16013976-005/2016/EPI_ISL_268678/NP/H5N8/2016-11-09
A/T_Dk/NL-Zeewolde/16013976-004-006/2016/EPI_ISL_268677/NP/H5N8/2016-11-09
A/mute_swan/Kaliningrad/132/2017/EPI_ISL_274858/NP/H5N8/2017-02-13
A/duck/India/10CA01/2016/EPI_ISL_237553/NP/H5N8/2016-10-17
A/mute_swan/Hungary/51049/2016/EPI_ISL_237730/NP/H5N8/2016-10-19
A/Turkey/Netherlands/12005615/2012/EPI_ISL_309826/NP/H10N9/2012-03-13
A/Turkey/Netherlands/12004763-001-004/2012/EPI_ISL_309825/NP/H10N9/2012-03-05
A/Chicken/Netherlands/12002495-001-005/2012/EPI_ISL_309809/NP/H10N9/2012-02-06
A/mallard_duck/Netherlands/12/2012/EPI_ISL_243384/NP/H6N1/2012-10-18
A/white-fronted_goose/Netherlands/5/2011/EPI_ISL_267292/NP/H6N8/2011-12-14
A/greater_white-fronted_goose/Netherlands/6/2011/EPI_ISL_243479/NP/H6N8/2011-12-14
A/mallard_duck/Netherlands/24/2009/EPI_ISL_243650/NP/H6N1/2009-12-01
A/mallard_duck/Netherlands/23/2013/EPI_ISL_267268/NP/H7N3/2013-04-09
A/mallard_duck/Netherlands/11/2013/EPI_ISL_328998/NP/H7N7/2013-12-09
A/mallard_duck/Netherlands/2/2014/EPI_ISL_267318/NP/H10N7/2014-02-17
A/mallard_duck/Netherlands/59/2015/EPI_ISL_328956/NP/H6N5/2015-09-05
A/mallard_duck/Netherlands/8/2014/EPI_ISL_267186/NP/H7N5/2014-10-28
A/mallard_duck/Netherlands/18/2012/EPI_ISL_267376/NP/H4N2/2012-08-21
A/mallard_duck/Netherlands/16/2012/EPI_ISL_267224/NP/H3N2/2012-08-17
A/mallard_duck/Netherlands/23/2012/EPI_ISL_243461/NP/H11N9/2012-09-27
A/mallard_duck/Netherlands/7/2015/EPI_ISL_328992/NP/H8N4/2015-01-03
A/mallard_duck/Netherlands/30/2014/EPI_ISL_243663/NP/H4N6/2014-07-21
A/mallard_duck/Netherlands/56/2015/EPI_ISL_328958/NP/H3N2/2015-08-22
A/barnacle_goose/Netherlands/2/2014/EPI_ISL_267243/NP/H3N6/2014-12-15
A/turkey/Germany-SH/R425/2017/EPI_ISL_243049/NP/H5N5/2017-01-22
A/cormorant/Germany-SH/R896/2017/EPI_ISL_260059/NP/H5N5/2017-01-30
A/Go/NL-Utrecht/17006881-001/2017/EPI_ISL_288411/NP/H5N5/2017-05-22
A/egret/Germany-SH/R1459/2017/EPI_ISL_259525/NP/H5N5/2017-02-14
A/domestic_duck/Siberia/50K/2016/EPI_ISL_240678/NP/H5N8/2016-10-10
A/chicken/Korea/H903/2017/EPI_ISL_254725/NP/H5N8/2017-02-08
A/Brown-headed_Gull/Qinghai/ZTO1-B/2016/EPI_ISL_224742/NP/H5N8/2016-05-01
A/duck/Mongolia/167/2015/EPI_ISL_209118/NP/H3N8/2015-08-30
A/duck/Mongolia/83/2015/EPI_ISL_207030/NP/H3N8/2015-08-30
A/duck/Mongolia/208/2015/EPI_ISL_209124/NP/H3N8/2015-08-31
A/duck/Mongolia/211/2015/EPI_ISL_209125/NP/H3N8/2015-08-31
A/duck/Ganzhou/GZ5/2015/EPI_ISL_252832/NP/H4N6/2015-12-12
A/duck/Vietnam/LBM300/2012/EPI ISL 138236/NP/H10N2/2012 (Month and day unknown)
A/duck/Mongolia/17/2011/EPI_ISL_294001/NP/H4N3/2011-08-26
A/duck/Mongolia/200/2015/EPI_ISL_209123/NP/H3N8/2015-08-31
A/wild_waterfowl/Dongting/C2383/2012/EPI_ISL_151927/NP/H1N2/2012-01-10
A/duck/Saga/411117/2013/EPI ISL 237137/NP/H6N1/2013-11 (Day unknown)
A/mallard_duck/Netherlands/41/2015/EPI_ISL_328966/NP/H5N1/2015-09-15
A/chicken/Astrakhan/3131/2016/EPI_ISL_240110/NP/H5N8/2016-12-13
A/mallard_duck/Netherlands/7/2014/EPI_ISL_243620/NP/H6N2/2014-10-01
A/mallard_duck/Netherlands/11/2014/EPI_ISL_267219/NP/H6N2/2014-11-22
A/mallard_duck/Netherlands/18/2015/EPI_ISL_328963/NP/H7N7/2015-06-27
A/common_teal/Netherlands/1/2015/EPI_ISL_328977/NP/H11N1/2015-09-29
A/chicken/Scotland/532/2016/EPI_ISL_205833/NP/H5N1/2016-01-09
A/Mallard/Netherlands/14016619/2014/EPI_ISL_291195/NP/H3N6/2014-12-03
A/mallard_duck/Georgia/10/2016/EPI_ISL_328976/NP/H7N7/2016-09-30
A/gadwall/Chany/97/2016/EPI_ISL_250238/NP/H6N8/2016-09-10
A/environment/Kamchatka/18/2016/EPI_ISL_256301/NP/H5N5/2016-10-01
A/M_Swan/NL-Groningen/16015826-001/2016/EPI_ISL_287565/NP/H5N5/2016-12-13
A/swan/Germany-SN/R10645/2016/EPI_ISL_240893/NP/H5N5/2016-12-13
A/grey_heron/Germany-SN/R572/2017/EPI_ISL_260058/NP/H5N5/2017-01-22
A/greylag_goose/Germany-NI/AR11353-L02142/2016/EPI_ISL_262056/NP/H5N5/2016-12-27
A/mute_swan/Croatia/102/2016/EPI_ISL_240101/NP/H5N5/2016-12-27
A/gadwall/Italy/17VIR133-2/2017/EPI_ISL_255189/NP/H5N5/2017-01-10
A/wigeon/Italy/16VIR9616-3/2016/EPI_ISL_243085/NP/H5N5/2016-12-29
A/mute_swan/Croatia/42/2017/EPI_ISL_243698/NP/H5N5/2017-01-20
A/chicken/Croatia/104/2017/EPI_ISL_261332/NP/H5N5/2017-03-07
A/Mute_swan/Hungary/5879/2017/EPI_ISL_256462/NP/H5N5/2017-02-14
A/common_buzzard/Germany-SN/R1117/2017/EPI_ISL_259074/NP/H5N5/2017-02-06
A/mute_swan/Poland/64/2017/EPI_ISL_255917/NP/H5N5/2017-01-31
1
0,73
0,84
0,95
0,98
0,76
1
0,87
1
0,97
0,81
0,96
0,92
0,96
0,94
0.006

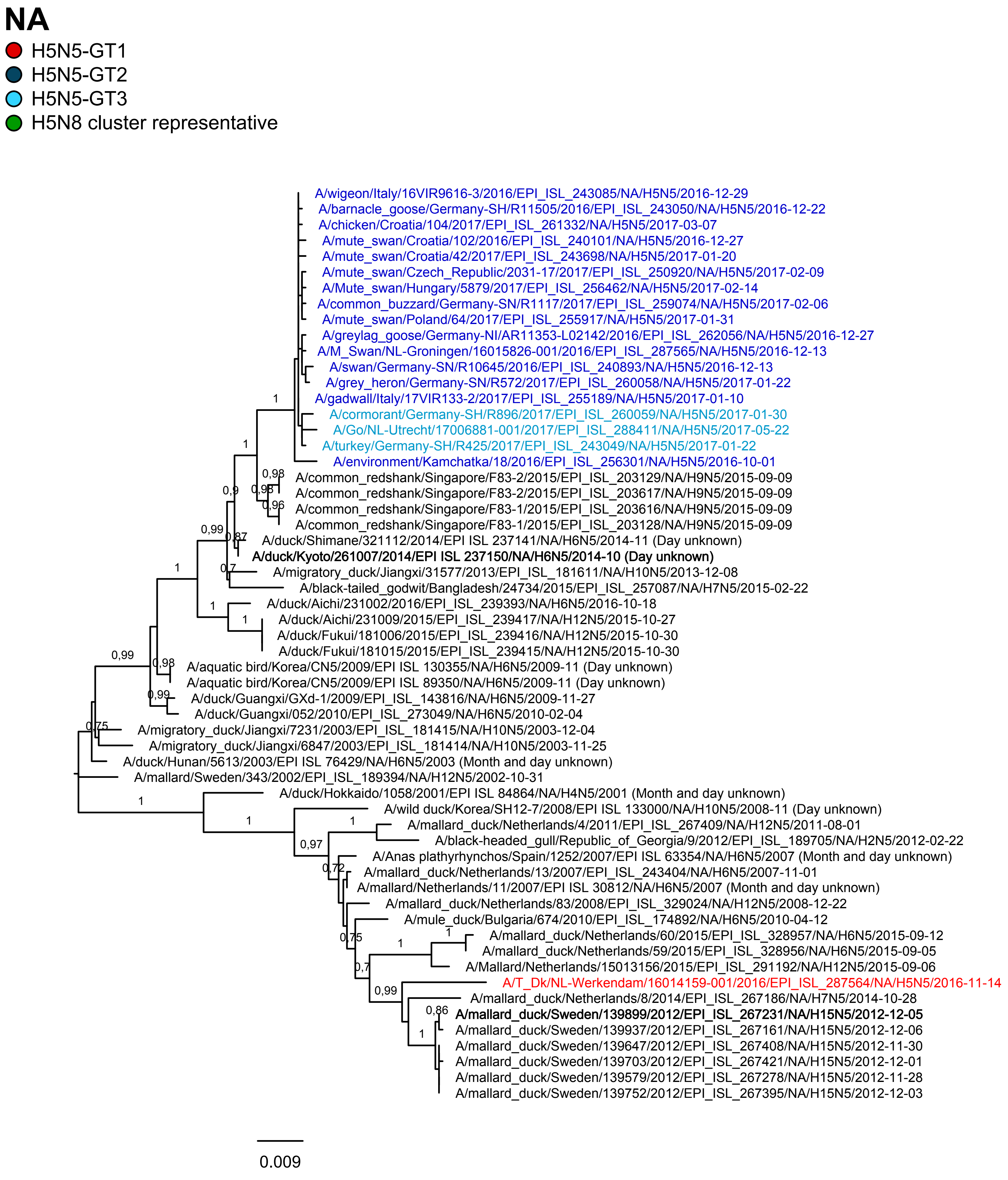
NA
H5N5-GT1
H5N5-GT2
H5N5-GT3
H5N8 cluster representative
A/wigeon/Italy/16VIR9616-3/2016/EPI_ISL_243085/NA/H5N5/2016-12-29
A/barnacle_goose/Germany-SH/R11505/2016/EPI_ISL_243050/NA/H5N5/2016-12-22
A/chicken/Croatia/104/2017/EPI_ISL_261332/NA/H5N5/2017-03-07
A/mute_swan/Croatia/102/2016/EPI_ISL_240101/NA/H5N5/2016-12-27
A/mute_swan/Croatia/42/2017/EPI_ISL_243698/NA/H5N5/2017-01-20
A/mute_swan/Czech_Republic/2031-17/2017/EPI_ISL_250920/NA/H5N5/2017-02-09
A/Mute_swan/Hungary/5879/2017/EPI_ISL_256462/NA/H5N5/2017-02-14
A/common_buzzard/Germany-SN/R1117/2017/EPI_ISL_259074/NA/H5N5/2017-02-06
A/mute_swan/Poland/64/2017/EPI_ISL_255917/NA/H5N5/2017-01-31
A/greylag_goose/Germany-NI/AR11353-L02142/2016/EPI_ISL_262056/NA/H5N5/2016-12-27
A/M_Swan/NL-Groningen/16015826-001/2016/EPI_ISL_287565/NA/H5N5/2016-12-13
A/swan/Germany-SN/R10645/2016/EPI_ISL_240893/NA/H5N5/2016-12-13
A/grey_heron/Germany-SN/R572/2017/EPI_ISL_260058/NA/H5N5/2017-01-22
A/gadwall/Italy/17VIR133-2/2017/EPI_ISL_255189/NA/H5N5/2017-01-10
A/cormorant/Germany-SH/R896/2017/EPI_ISL_260059/NA/H5N5/2017-01-30
A/Go/NL-Utrecht/17006881-001/2017/EPI_ISL_288411/NA/H5N5/2017-05-22
A/turkey/Germany-SH/R425/2017/EPI_ISL_243049/NA/H5N5/2017-01-22
A/environment/Kamchatka/18/2016/EPI_ISL_256301/NA/H5N5/2016-10-01
A/common_redshank/Singapore/F83-2/2015/EPI_ISL_203129/NA/H9N5/2015-09-09
A/common_redshank/Singapore/F83-2/2015/EPI_ISL_203617/NA/H9N5/2015-09-09
A/common_redshank/Singapore/F83-1/2015/EPI_ISL_203616/NA/H9N5/2015-09-09
A/common_redshank/Singapore/F83-1/2015/EPI_ISL_203128/NA/H9N5/2015-09-09
A/duck/Shimane/321112/2014/EPI ISL 237141/NA/H6N5/2014-11 (Day unknown)
A/duck/Kyoto/261007/2014/EPI ISL 237150/NA/H6N5/2014-10 (Day unknown)
A/migratory_duck/Jiangxi/31577/2013/EPI_ISL_181611/NA/H10N5/2013-12-08
A/black-tailed_godwit/Bangladesh/24734/2015/EPI_ISL_257087/NA/H7N5/2015-02-22
A/duck/Aichi/231002/2016/EPI_ISL_239393/NA/H6N5/2016-10-18
A/duck/Aichi/231009/2015/EPI_ISL_239417/NA/H12N5/2015-10-27
A/duck/Fukui/181006/2015/EPI_ISL_239416/NA/H12N5/2015-10-30
A/duck/Fukui/181015/2015/EPI_ISL_239415/NA/H12N5/2015-10-30
A/aquatic bird/Korea/CN5/2009/EPI ISL 130355/NA/H6N5/2009-11 (Day unknown)
A/aquatic bird/Korea/CN5/2009/EPI ISL 89350/NA/H6N5/2009-11 (Day unknown)
A/duck/Guangxi/GXd-1/2009/EPI_ISL_143816/NA/H6N5/2009-11-27
A/duck/Guangxi/052/2010/EPI_ISL_273049/NA/H6N5/2010-02-04
A/migratory_duck/Jiangxi/7231/2003/EPI_ISL_181415/NA/H10N5/2003-12-04
A/migratory_duck/Jiangxi/6847/2003/EPI_ISL_181414/NA/H10N5/2003-11-25
A/duck/Hunan/5613/2003/EPI ISL 76429/NA/H6N5/2003 (Month and day unknown)
A/mallard/Sweden/343/2002/EPI_ISL_189394/NA/H12N5/2002-10-31
A/duck/Hokkaido/1058/2001/EPI ISL 84864/NA/H4N5/2001 (Month and day unknown)
A/wild duck/Korea/SH12-7/2008/EPI ISL 133000/NA/H10N5/2008-11 (Day unknown)
A/mallard_duck/Netherlands/4/2011/EPI_ISL_267409/NA/H12N5/2011-08-01
A/black-headed_gull/Republic_of_Georgia/9/2012/EPI_ISL_189705/NA/H2N5/2012-02-22
A/Anas plathyrhynchos/Spain/1252/2007/EPI ISL 63354/NA/H6N5/2007 (Month and day unknown)
A/mallard_duck/Netherlands/13/2007/EPI_ISL_243404/NA/H6N5/2007-11-01
A/mallard/Netherlands/11/2007/EPI ISL 30812/NA/H6N5/2007 (Month and day unknown)
A/mallard_duck/Netherlands/83/2008/EPI_ISL_329024/NA/H12N5/2008-12-22
A/mule_duck/Bulgaria/674/2010/EPI_ISL_174892/NA/H6N5/2010-04-12
A/mallard_duck/Netherlands/60/2015/EPI_ISL_328957/NA/H6N5/2015-09-12
A/mallard_duck/Netherlands/59/2015/EPI_ISL_328956/NA/H6N5/2015-09-05
A/Mallard/Netherlands/15013156/2015/EPI_ISL_291192/NA/H12N5/2015-09-06
A/T_Dk/NL-Werkendam/16014159-001/2016/EPI_ISL_287564/NA/H5N5/2016-11-14
A/mallard_duck/Netherlands/8/2014/EPI_ISL_267186/NA/H7N5/2014-10-28
A/mallard_duck/Sweden/139899/2012/EPI_ISL_267231/NA/H15N5/2012-12-05
A/mallard_duck/Sweden/139937/2012/EPI_ISL_267161/NA/H15N5/2012-12-06
A/mallard_duck/Sweden/139647/2012/EPI_ISL_267408/NA/H15N5/2012-11-30
A/mallard_duck/Sweden/139703/2012/EPI_ISL_267421/NA/H15N5/2012-12-01
A/mallard_duck/Sweden/139579/2012/EPI_ISL_267278/NA/H15N5/2012-11-28
A/mallard_duck/Sweden/139752/2012/EPI_ISL_267395/NA/H15N5/2012-12-03
0.009

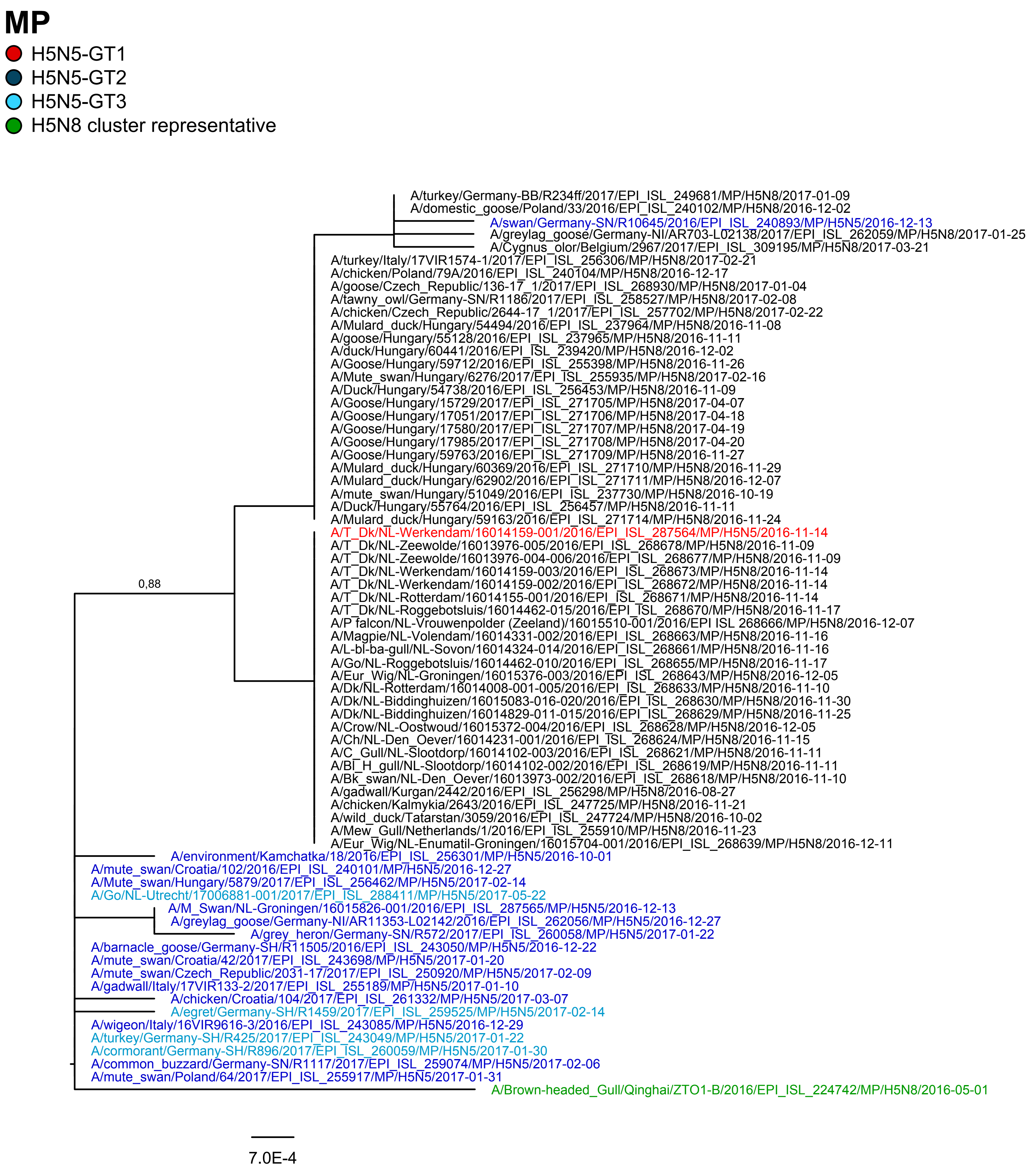
MP
H5N5-GT1
H5N5-GT2
H5N5-GT3
H5N8 cluster representative
A/turkey/Germany-BB/R234ff/2017/EPI_ISL_249681/MP/H5N8/2017-01-09
A/domestic_goose/Poland/33/2016/EPI_ISL_240102/MP/H5N8/2016-12-02
A/swan/Germany-SN/R10645/2016/EPI_ISL_240893/MP/H5N5/2016-12-13
A/greylag_goose/Germany-NI/AR703-L02138/2017/EPI_ISL_262059/MP/H5N8/2017-01-25
A/Cygnus_olor/Belgium/2967/2017/EPI_ISL_309195/MP/H5N8/2017-03-21
A/turkey/Italy/17VIR1574-1/2017/EPI_ISL_256306/MP/H5N8/2017-02-21
A/chicken/Poland/79A/2016/EPI_ISL_240104/MP/H5N8/2016-12-17
A/goose/Czech_Republic/136-17_1/2017/EPI_ISL_268930/MP/H5N8/2017-01-04
A/tawny_owl/Germany-SN/R1186/2017/EPI_ISL_258527/MP/H5N8/2017-02-08
A/chicken/Czech_Republic/2644-17_1/2017/EPI_ISL_257702/MP/H5N8/2017-02-22
A/Mulard_duck/Hungary/54494/2016/EPI_ISL_237964/MP/H5N8/2016-11-08
A/goose/Hungary/55128/2016/EPI_ISL_237965/MP/H5N8/2016-11-11
A/duck/Hungary/60441/2016/EPI_ISL_239420/MP/H5N8/2016-12-02
A/Goose/Hungary/59712/2016/EPI_ISL_255398/MP/H5N8/2016-11-26
A/Mute_swan/Hungary/6276/2017/EPI_ISL_255935/MP/H5N8/2017-02-16
A/Duck/Hungary/54738/2016/EPI_ISL_256453/MP/H5N8/2016-11-09
A/Goose/Hungary/15729/2017/EPI_ISL_271705/MP/H5N8/2017-04-07
A/Goose/Hungary/17051/2017/EPI_ISL_271706/MP/H5N8/2017-04-18
A/Goose/Hungary/17580/2017/EPI_ISL_271707/MP/H5N8/2017-04-19
A/Goose/Hungary/17985/2017/EPI_ISL_271708/MP/H5N8/2017-04-20
A/Goose/Hungary/59763/2016/EPI_ISL_271709/MP/H5N8/2016-11-27
A/Mulard_duck/Hungary/60369/2016/EPI_ISL_271710/MP/H5N8/2016-11-29
A/Mulard_duck/Hungary/62902/2016/EPI_ISL_271711/MP/H5N8/2016-12-07
A/mute_swan/Hungary/51049/2016/EPI_ISL_237730/MP/H5N8/2016-10-19
A/Duck/Hungary/55764/2016/EPI_ISL_256457/MP/H5N8/2016-11-11
A/Mulard_duck/Hungary/59163/2016/EPI_ISL_271714/MP/H5N8/2016-11-24
A/T_Dk/NL-Werkendam/16014159-001/2016/EPI_ISL_287564/MP/H5N5/2016-11-14
A/T_Dk/NL-Zeewolde/16013976-005/2016/EPI_ISL_268678/MP/H5N8/2016-11-09
A/T_Dk/NL-Zeewolde/16013976-004-006/2016/EPI_ISL_268677/MP/H5N8/2016-11-09
A/T_Dk/NL-Werkendam/16014159-003/2016/EPI_ISL_268673/MP/H5N8/2016-11-14
A/T_Dk/NL-Werkendam/16014159-002/2016/EPI_ISL_268672/MP/H5N8/2016-11-14
A/T_Dk/NL-Rotterdam/16014155-001/2016/EPI_ISL_268671/MP/H5N8/2016-11-14
A/T_Dk/NL-Roggebotsluis/16014462-015/2016/EPI_ISL_268670/MP/H5N8/2016-11-17
A/P falcon/NL-Vrouwenpolder (Zeeland)/16015510-001/2016/EPI ISL 268666/MP/H5N8/2016-12-07
A/Magpie/NL-Volendam/16014331-002/2016/EPI_ISL_268663/MP/H5N8/2016-11-16
A/L-bl-ba-gull/NL-Sovon/16014324-014/2016/EPI_ISL_268661/MP/H5N8/2016-11-16
A/Go/NL-Roggebotsluis/16014462-010/2016/EPI_ISL_268655/MP/H5N8/2016-11-17
A/Eur_Wig/NL-Groningen/16015376-003/2016/EPI_ISL_268643/MP/H5N8/2016-12-05
A/Dk/NL-Rotterdam/16014008-001-005/2016/EPI_ISL_268633/MP/H5N8/2016-11-10
A/Dk/NL-Biddinghuizen/16015083-016-020/2016/EPI_ISL_268630/MP/H5N8/2016-11-30
A/Dk/NL-Biddinghuizen/16014829-011-015/2016/EPI_ISL_268629/MP/H5N8/2016-11-25
A/Crow/NL-Oostwoud/16015372-004/2016/EPI_ISL_268628/MP/H5N8/2016-12-05
A/Ch/NL-Den_Oever/16014231-001/2016/EPI_ISL_268624/MP/H5N8/2016-11-15
A/C_Gull/NL-Slootdorp/16014102-003/2016/EPI_ISL_268621/MP/H5N8/2016-11-11
A/Bl_H_gull/NL-Slootdorp/16014102-002/2016/EPI_ISL_268619/MP/H5N8/2016-11-11
A/Bk_swan/NL-Den_Oever/16013973-002/2016/EPI_ISL_268618/MP/H5N8/2016-11-10
A/gadwall/Kurgan/2442/2016/EPI_ISL_256298/MP/H5N8/2016-08-27
A/chicken/Kalmykia/2643/2016/EPI_ISL_247725/MP/H5N8/2016-11-21
A/wild_duck/Tatarstan/3059/2016/EPI_ISL_247724/MP/H5N8/2016-10-02
A/Mew_Gull/Netherlands/1/2016/EPI_ISL_255910/MP/H5N8/2016-11-23
A/Eur_Wig/NL-Enumatil-Groningen/16015704-001/2016/EPI_ISL_268639/MP/H5N8/2016-12-11
0,88
A/environment/Kamchatka/18/2016/EPI_ISL_256301/MP/H5N5/2016-10-01
A/mute_swan/Croatia/102/2016/EPI_ISL_240101/MP/H5N5/2016-12-27
A/Mute_swan/Hungary/5879/2017/EPI_ISL_256462/MP/H5N5/2017-02-14
A/Go/NL-Utrecht/17006881-001/2017/EPI_ISL_288411/MP/H5N5/2017-05-22
A/M_Swan/NL-Groningen/16015826-001/2016/EPI_ISL_287565/MP/H5N5/2016-12-13
A/greylag_goose/Germany-NI/AR11353-L02142/2016/EPI_ISL_262056/MP/H5N5/2016-12-27
A/grey_heron/Germany-SN/R572/2017/EPI_ISL_260058/MP/H5N5/2017-01-22
A/barnacle_goose/Germany-SH/R11505/2016/EPI_ISL_243050/MP/H5N5/2016-12-22
A/mute_swan/Croatia/42/2017/EPI_ISL_243698/MP/H5N5/2017-01-20
A/mute_swan/Czech_Republic/2031-17/2017/EPI_ISL_250920/MP/H5N5/2017-02-09
A/gadwall/Italy/17VIR133-2/2017/EPI_ISL_255189/MP/H5N5/2017-01-10
A/chicken/Croatia/104/2017/EPI_ISL_261332/MP/H5N5/2017-03-07
A/egret/Germany-SH/R1459/2017/EPI_ISL_259525/MP/H5N5/2017-02-14
A/wigeon/Italy/16VIR9616-3/2016/EPI_ISL_243085/MP/H5N5/2016-12-29
A/turkey/Germany-SH/R425/2017/EPI_ISL_243049/MP/H5N5/2017-01-22
A/cormorant/Germany-SH/R896/2017/EPI_ISL_260059/MP/H5N5/2017-01-30
A/common_buzzard/Germany-SN/R1117/2017/EPI_ISL_259074/MP/H5N5/2017-02-06
A/mute_swan/Poland/64/2017/EPI_ISL_255917/MP/H5N5/2017-01-31
A/Brown-headed_Gull/Qinghai/ZTO1-B/2016/EPI_ISL_224742/MP/H5N8/2016-05-01
7.0E-4

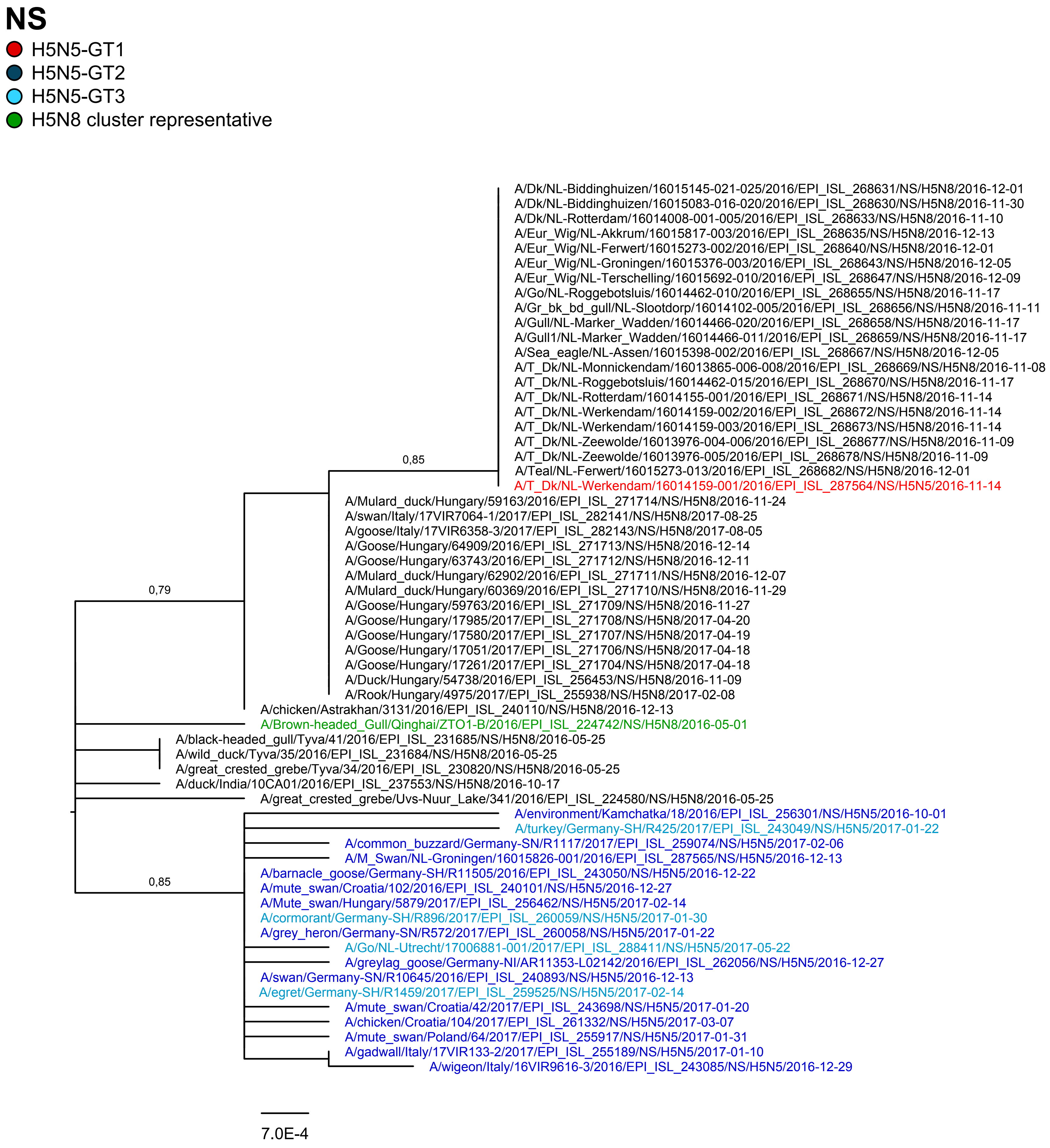
NS
H5N5-GT1
H5N5-GT2
H5N5-GT3
H5N8 cluster representative
A/Dk/NL-Biddinghuizen/16015145-021-025/2016/EPI_ISL_268631/NS/H5N8/2016-12-01
A/Dk/NL-Biddinghuizen/16015083-016-020/2016/EPI_ISL_268630/NS/H5N8/2016-11-30
A/Dk/NL-Rotterdam/16014008-001-005/2016/EPI_ISL_268633/NS/H5N8/2016-11-10
A/Eur_Wig/NL-Akkrum/16015817-003/2016/EPI_ISL_268635/NS/H5N8/2016-12-13
A/Eur_Wig/NL-Ferwert/16015273-002/2016/EPI_ISL_268640/NS/H5N8/2016-12-01
A/Eur_Wig/NL-Groningen/16015376-003/2016/EPI_ISL_268643/NS/H5N8/2016-12-05
A/Eur_Wig/NL-Terschelling/16015692-010/2016/EPI_ISL_268647/NS/H5N8/2016-12-09
A/Go/NL-Roggebotsluis/16014462-010/2016/EPI_ISL_268655/NS/H5N8/2016-11-17
A/Gr_bk_bd_gull/NL-Slootdorp/16014102-005/2016/EPI_ISL_268656/NS/H5N8/2016-11-11
A/Gull/NL-Marker_Wadden/16014466-020/2016/EPI_ISL_268658/NS/H5N8/2016-11-17
A/Gull1/NL-Marker_Wadden/16014466-011/2016/EPI_ISL_268659/NS/H5N8/2016-11-17
A/Sea_eagle/NL-Assen/16015398-002/2016/EPI_ISL_268667/NS/H5N8/2016-12-05
A/T_Dk/NL-Monnickendam/16013865-006-008/2016/EPI_ISL_268669/NS/H5N8/2016-11-08
A/T_Dk/NL-Roggebotsluis/16014462-015/2016/EPI_ISL_268670/NS/H5N8/2016-11-17
A/T_Dk/NL-Rotterdam/16014155-001/2016/EPI_ISL_268671/NS/H5N8/2016-11-14
A/T_Dk/NL-Werkendam/16014159-002/2016/EPI_ISL_268672/NS/H5N8/2016-11-14
A/T_Dk/NL-Werkendam/16014159-003/2016/EPI_ISL_268673/NS/H5N8/2016-11-14
A/T_Dk/NL-Zeewolde/16013976-004-006/2016/EPI_ISL_268677/NS/H5N8/2016-11-09
A/T_Dk/NL-Zeewolde/16013976-005/2016/EPI_ISL_268678/NS/H5N8/2016-11-09
0,85
A/Teal/NL-Ferwert/16015273-013/2016/EPI_ISL_268682/NS/H5N8/2016-12-01
A/T_Dk/NL-Werkendam/16014159-001/2016/EPI_ISL_287564/NS/H5N5/2016-11-14
A/Mulard_duck/Hungary/59163/2016/EPI_ISL_271714/NS/H5N8/2016-11-24
A/swan/Italy/17VIR7064-1/2017/EPI_ISL_282141/NS/H5N8/2017-08-25
A/goose/Italy/17VIR6358-3/2017/EPI_ISL_282143/NS/H5N8/2017-08-05
A/Goose/Hungary/64909/2016/EPI_ISL_271713/NS/H5N8/2016-12-14
A/Goose/Hungary/63743/2016/EPI_ISL_271712/NS/H5N8/2016-12-11
A/Mulard_duck/Hungary/62902/2016/EPI_ISL_271711/NS/H5N8/2016-12-07
0,79
A/Mulard_duck/Hungary/60369/2016/EPI_ISL_271710/NS/H5N8/2016-11-29
A/Goose/Hungary/59763/2016/EPI_ISL_271709/NS/H5N8/2016-11-27
A/Goose/Hungary/17985/2017/EPI_ISL_271708/NS/H5N8/2017-04-20
A/Goose/Hungary/17580/2017/EPI_ISL_271707/NS/H5N8/2017-04-19
A/Goose/Hungary/17051/2017/EPI_ISL_271706/NS/H5N8/2017-04-18
A/Goose/Hungary/17261/2017/EPI_ISL_271704/NS/H5N8/2017-04-18
A/Duck/Hungary/54738/2016/EPI_ISL_256453/NS/H5N8/2016-11-09
A/Rook/Hungary/4975/2017/EPI_ISL_255938/NS/H5N8/2017-02-08
A/chicken/Astrakhan/3131/2016/EPI_ISL_240110/NS/H5N8/2016-12-13
A/Brown-headed_Gull/Qinghai/ZTO1-B/2016/EPI_ISL_224742/NS/H5N8/2016-05-01
A/black-headed_gull/Tyva/41/2016/EPI_ISL_231685/NS/H5N8/2016-05-25
A/wild_duck/Tyva/35/2016/EPI_ISL_231684/NS/H5N8/2016-05-25
A/great_crested_grebe/Tyva/34/2016/EPI_ISL_230820/NS/H5N8/2016-05-25
A/duck/India/10CA01/2016/EPI_ISL_237553/NS/H5N8/2016-10-17
A/great_crested_grebe/Uvs-Nuur_Lake/341/2016/EPI_ISL_224580/NS/H5N8/2016-05-25
A/environment/Kamchatka/18/2016/EPI_ISL_256301/NS/H5N5/2016-10-01
A/turkey/Germany-SH/R425/2017/EPI_ISL_243049/NS/H5N5/2017-01-22
A/common_buzzard/Germany-SN/R1117/2017/EPI_ISL_259074/NS/H5N5/2017-02-06
A/M_Swan/NL-Groningen/16015826-001/2016/EPI_ISL_287565/NS/H5N5/2016-12-13
0,85
A/barnacle_goose/Germany-SH/R11505/2016/EPI_ISL_243050/NS/H5N5/2016-12-22
A/mute_swan/Croatia/102/2016/EPI_ISL_240101/NS/H5N5/2016-12-27
A/Mute_swan/Hungary/5879/2017/EPI_ISL_256462/NS/H5N5/2017-02-14
A/cormorant/Germany-SH/R896/2017/EPI_ISL_260059/NS/H5N5/2017-01-30
A/grey_heron/Germany-SN/R572/2017/EPI_ISL_260058/NS/H5N5/2017-01-22
A/Go/NL-Utrecht/17006881-001/2017/EPI_ISL_288411/NS/H5N5/2017-05-22
A/greylag_goose/Germany-NI/AR11353-L02142/2016/EPI_ISL_262056/NS/H5N5/2016-12-27
A/swan/Germany-SN/R10645/2016/EPI_ISL_240893/NS/H5N5/2016-12-13
A/egret/Germany-SH/R1459/2017/EPI_ISL_259525/NS/H5N5/2017-02-14
A/mute_swan/Croatia/42/2017/EPI_ISL_243698/NS/H5N5/2017-01-20
A/chicken/Croatia/104/2017/EPI_ISL_261332/NS/H5N5/2017-03-07
A/mute_swan/Poland/64/2017/EPI_ISL_255917/NS/H5N5/2017-01-31
A/gadwall/Italy/17VIR133-2/2017/EPI_ISL_255189/NS/H5N5/2017-01-10
A/wigeon/Italy/16VIR9616-3/2016/EPI_ISL_243085/NS/H5N5/2016-12-29
7.0E-4
