# Supplementary material for: Spread of Highly Pathogenic Avian Influenza (HPAI) H5N5 Viruses in Europe in 2016–2017 Appears Related to the Timing of Reassortment Events
Source: Viruses. 2019 May 31;11(6):501. doi: 10.3390/v11060501 (PMC6631432; doi:10.3390/v11060501)
Supplement: Supplementary file 1 [file viruses-11-00501-s001.zip › FigS2_revised.pdf]

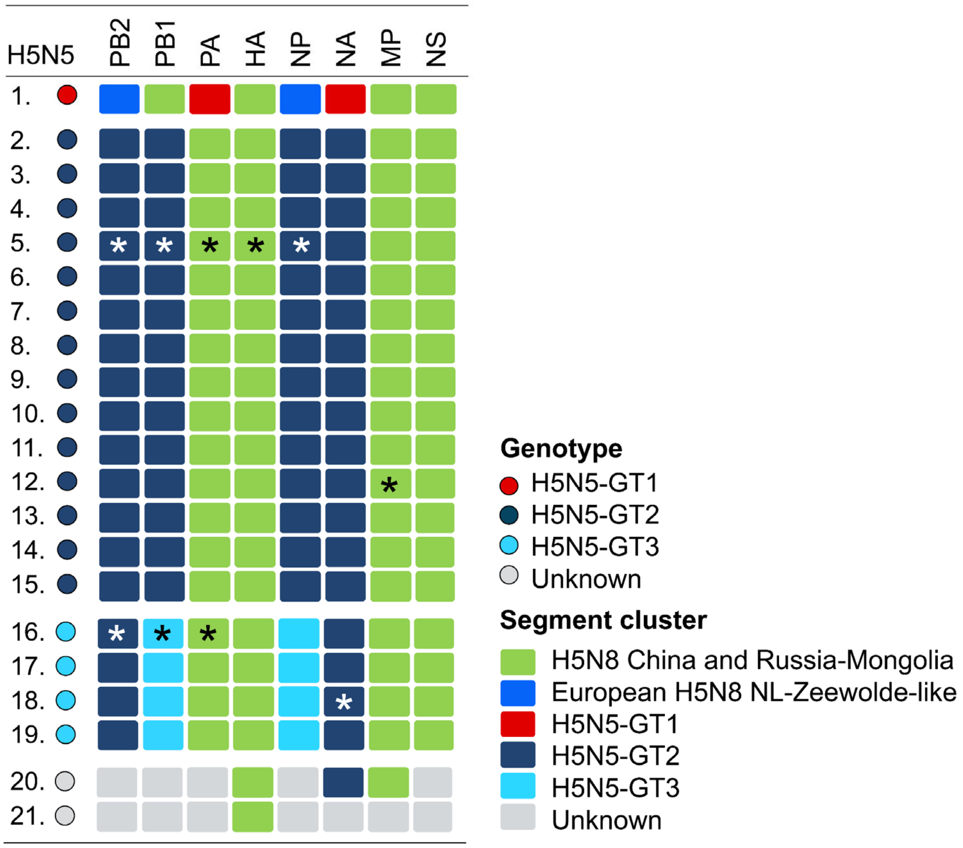

Figure S2. Gene constellations of H5N5 viruses. Highly pathogenic avian influenza (HPAI) H5N5 viruses isolated during the HPAI H5 2016-2017 epizootic, ordered by genotype. Asterisks indicate incomplete sequences. Gene segments are coloured according to their phylogenetic cluster. Detailed information on the virus sequences is provided in Table S2. PB2, polymerase basic protein 2; PB1, polymerase basic protein 1; PA, polymerase acidic protein; HA, hemagglutinin; NP, nucleoprotein; NA, neuraminidase; MP, matrix protein; NS, nonstructural protein.
