# Supplementary material for: Spread of Highly Pathogenic Avian Influenza (HPAI) H5N5 Viruses in Europe in 2016–2017 Appears Related to the Timing of Reassortment Events
Source: Viruses. 2019 May 31;11(6):501. doi: 10.3390/v11060501 (PMC6631432; doi:10.3390/v11060501)
Supplement: Supplementary file 1 [file viruses-11-00501-s001.zip › FigS3_revised.pdf]

Figure S3. Maximum clade credibility trees. Maximum clade credibility (MCC) trees were generated to estimate the median time to the most recent common ancestor (TMRCA) for each gene segment of highly pathogenic avian influenza (HPAI) H5N5 viruses isolated during the HPAI H5 2016-2017 epizootic. The H5N5 virus sequences are coloured according to their genotype (H5N5-GT1, red; H5N5-GT2, dark blue; H5N5-GT3, light blue). The median TMRCAs estimates were determined for H5N5-GT2 viruses (node 1), European H5N5-GT2 viruses (node 2), and H5N5-GT3 viruses (node 3). The estimated TMRCAs, the 95% highest posterior density (HPD) intervals and posterior values for each of the numbered nodes are listed in Table 2. PB2, polymerase basic 2; PB1, polymerase basic 1; PA, polymerase acidic; HA, hemagglutinin; NP, nucleoprotein; NA, neuraminidase; MP, matrix protein; NS, nonstructural protein.

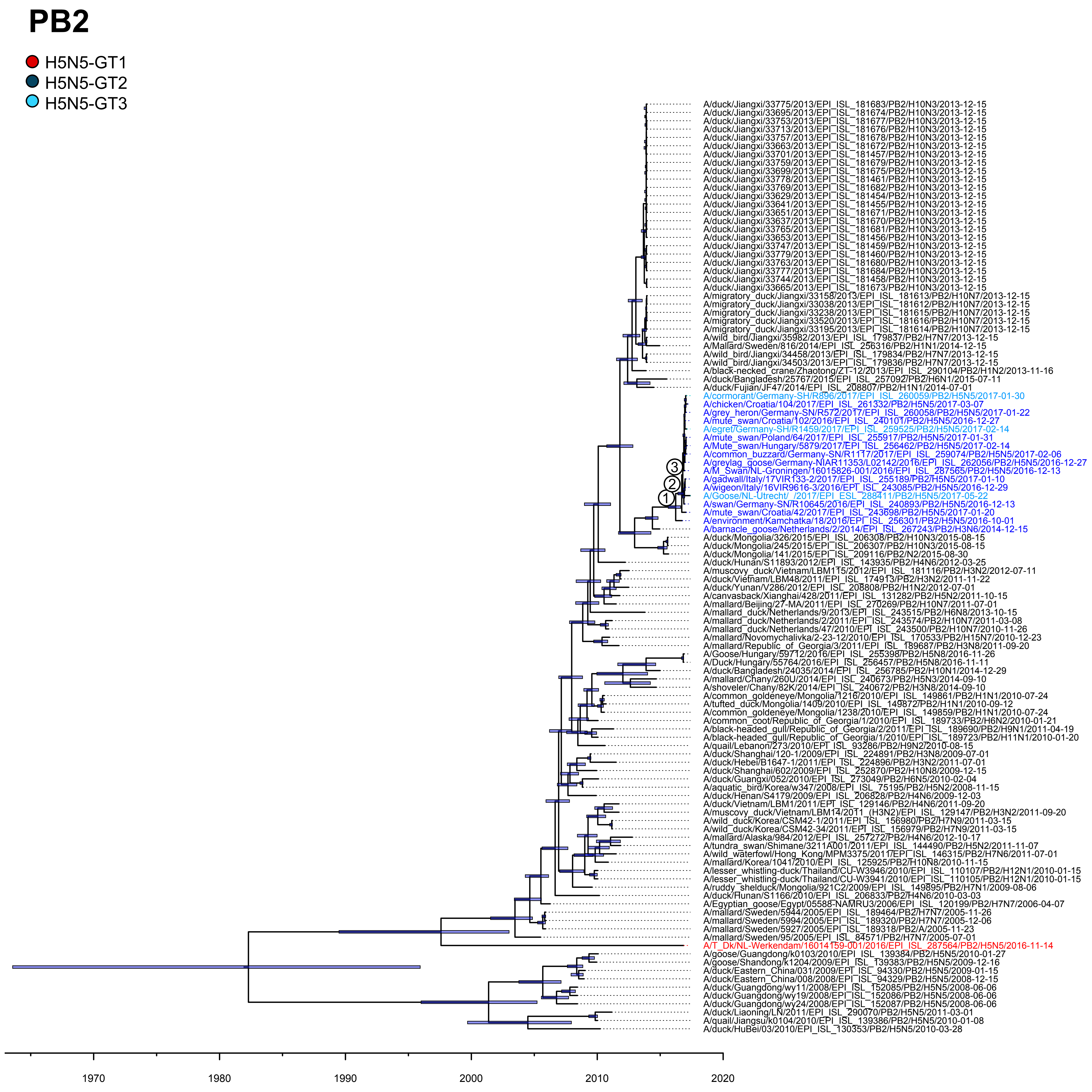
PB2
H5N5-GT1
H5N5-GT2
H5N5-GT3
A/duck/Jiangxi/33775/2013/EPI_ISL_181683/PB2/H10N3/2013-12-15
A/duck/Jiangxi/33695/2013/EPI_ISL_181674/PB2/H10N3/2013-12-15
A/duck/Jiangxi/33753/2013/EPI_ISL_181677/PB2/H10N3/2013-12-15
A/duck/Jiangxi/33713/2013/EPI_ISL_181676/PB2/H10N3/2013-12-15
A/duck/Jiangxi/33757/2013/EPI_ISL_181678/PB2/H10N3/2013-12-15
A/duck/Jiangxi/33663/2013/EPI_ISL_181672/PB2/H10N3/2013-12-15
A/duck/Jiangxi/33701/2013/EPI_ISL_181457/PB2/H10N3/2013-12-15
A/duck/Jiangxi/33759/2013/EPI_ISL_181679/PB2/H10N3/2013-12-15
A/duck/Jiangxi/33699/2013/EPI_ISL_181675/PB2/H10N3/2013-12-15
A/duck/Jiangxi/33778/2013/EPI_ISL_181461/PB2/H10N3/2013-12-15
A/duck/Jiangxi/33769/2013/EPI_ISL_181682/PB2/H10N3/2013-12-15
A/duck/Jiangxi/33629/2013/EPI_ISL_181454/PB2/H10N3/2013-12-15
A/duck/Jiangxi/33641/2013/EPI_ISL_181455/PB2/H10N3/2013-12-15
A/duck/Jiangxi/33651/2013/EPI_ISL_181671/PB2/H10N3/2013-12-15
A/duck/Jiangxi/33637/2013/EPI_ISL_181670/PB2/H10N3/2013-12-15
A/duck/Jiangxi/33765/2013/EPI_ISL_181681/PB2/H10N3/2013-12-15
A/duck/Jiangxi/33653/2013/EPI_ISL_181456/PB2/H10N3/2013-12-15
A/duck/Jiangxi/33747/2013/EPI_ISL_181459/PB2/H10N3/2013-12-15
A/duck/Jiangxi/33779/2013/EPI_ISL_181460/PB2/H10N3/2013-12-15
A/duck/Jiangxi/33763/2013/EPI_ISL_181680/PB2/H10N3/2013-12-15
A/duck/Jiangxi/33777/2013/EPI_ISL_181684/PB2/H10N3/2013-12-15
A/duck/Jiangxi/33744/2013/EPI_ISL_181458/PB2/H10N3/2013-12-15
A/duck/Jiangxi/33665/2013/EPI_ISL_181673/PB2/H10N3/2013-12-15
A/migratory_duck/Jiangxi/33158/2013/EPI_ISL_181613/PB2/H10N7/2013-12-15
A/migratory_duck/Jiangxi/33038/2013/EPI_ISL_181612/PB2/H10N7/2013-12-15
A/migratory_duck/Jiangxi/33238/2013/EPI_ISL_181615/PB2/H10N7/2013-12-15
A/migratory_duck/Jiangxi/33520/2013/EPI_ISL_181616/PB2/H10N7/2013-12-15
A/migratory_duck/Jiangxi/33195/2013/EPI_ISL_181614/PB2/H10N7/2013-12-15
A/wild_bird/Jiangxi/35982/2013/EPI_ISL_179837/PB2/H7N7/2013-12-15
A/Mallard/Sweden/816/2014/EPI_ISL_256316/PB2/H1N1/2014-12-15
A/wild_bird/Jiangxi/34458/2013/EPI_ISL_179834/PB2/H7N7/2013-12-15
A/wild_bird/Jiangxi/34503/2013/EPI_ISL_179836/PB2/H7N7/2013-12-15
A/black-necked_crane/Zhaotong/ZT-12/2013/EPI_ISL_290104/PB2/H1N2/2013-11-16
A/duck/Bangladesh/25767/2015/EPI_ISL_257092/PB2/H6N1/2015-07-11
A/duck/Fujian/JF47/2014/EPI_ISL_208807/PB2/H1N1/2014-07-01
A/cormorant/Germany-SH/R896/2017/EPI_ISL_260059/PB2/H5N5/2017-01-30
A/chicken/Croatia/104/2017/EPI_ISL_261332/PB2/H5N5/2017-03-07
A/grey_heron/Germany-SN/R572/2017/EPI_ISL_260058/PB2/H5N5/2017-01-22
A/mute_swan/Croatia/102/2016/EPI_ISL_240101/PB2/H5N5/2016-12-27
A/egret/Germany-SH/R1459/2017/EPI_ISL_259525/PB2/H5N5/2017-02-14
A/mute_swan/Poland/64/2017/EPI_ISL_255917/PB2/H5N5/2017-01-31
A/Mute_swan/Hungary/5879/2017/EPI_ISL_256462/PB2/H5N5/2017-02-14
A/common_buzzard/Germany-SN/R1117/2017/EPI_ISL_259074/PB2/H5N5/2017-02-06
A/greylag_goose/Germany-NIAR11353/L02142/2016/EPI_ISL_262056/PB2/H5N5/2016-12-27
A/M_Swan/NL-Groningen/16015826-001/2016/EPI_ISL_287565/PB2/H5N5/2016-12-13
A/gadwall/Italy/17VIR133-2/2017/EPI_ISL_255189/PB2/H5N5/2017-01-10
A/wigeon/Italy/16VIR9616-3/2016/EPI_ISL_243085/PB2/H5N5/2016-12-29
A/Goose/NL-Utrecht/_/2017/EPI_ESL_288411/PB2/H5N5/2017-05-22
A/swan/Germany-SN/R10645/2016/EPI_ISL_240893/PB2/H5N5/2016-12-13
A/mute_swan/Croatia/42/2017/EPI_ISL_243698/PB2/H5N5/2017-01-20
A/environment/Kamchatka/18/2016/EPI_ISL_256301/PB2/H5N5/2016-10-01
A/barnacle_goose/Netherlands/2/2014/EPI_ISL_267243/PB2/H3N6/2014-12-15
3
2
1
A/duck/Mongolia/326/2015/EPI_ISL_206308/PB2/H10N3/2015-08-15
A/duck/Mongolia/245/2015/EPI_ISL_206307/PB2/H10N3/2015-08-15
A/duck/Mongolia/141/2015/EPI_ISL_209116/PB2/N2/2015-08-30
A/duck/Hunan/S11893/2012/EPI_ISL_143935/PB2/H4N6/2012-03-25
A/muscovy_duck/Vietnam/LBM115/2012/EPI_ISL_181116/PB2/H3N2/2012-07-11
A/duck/Vietnam/LBM48/2011/EPI_ISL_174913/PB2/H3N2/2011-11-22
A/duck/Yunan/V286/2012/EPI_ISL_208808/PB2/H1N2/2012-07-01
A/canvasback/Xianghai/428/2011/EPI_ISL_131282/PB2/H5N2/2011-10-15
A/mallard/Beijing/27-MA/2011/EPI_ISL_270269/PB2/H10N7/2011-07-01
A/mallard_duck/Netherlands/9/2013/EPI_ISL_243515/PB2/H6N8/2013-10-15
A/mallard_duck/Netherlands/2/2011/EPI_ISL_243574/PB2/H10N7/2011-03-08
A/mallard_duck/Netherlands/47/2010/EPI_ISL_243500/PB2/H10N7/2010-11-26
A/mallard/Novomychalivka/2-23-12/2010/EPI_ISL_170533/PB2/H15N7/2010-12-23
A/mallard/Republic_of_Georgia/3/2011/EPI_ISL_189687/PB2/H3N8/2011-09-20
A/Goose/Hungary/59712/2016/EPI_ISL_255398/PB2/H5N8/2016-11-26
A/Duck/Hungary/55764/2016/EPI_ISL_256457/PB2/H5N8/2016-11-11
A/duck/Bangladesh/24035/2014/EPI_ISL_256785/PB2/H10N1/2014-12-29
A/mallard/Chany/260U/2014/EPI_ISL_240673/PB2/H5N3/2014-09-10
A/shoveler/Chany/82K/2014/EPI_ISL_240672/PB2/H3N8/2014-09-10
A/common_goldeneye/Mongolia/1216/2010/EPI_ISL_149861/PB2/H1N1/2010-07-24
A/tufted_duck/Mongolia/1409/2010/EPI_ISL_149872/PB2/H1N1/2010-09-12
A/common_goldeneye/Mongolia/1238/2010/EPI_ISL_149859/PB2/H1N1/2010-07-24
A/common_coot/Republic_of_Georgia/1/2010/EPI_ISL_189733/PB2/H6N2/2010-01-21
A/black-headed_gull/Republic_of_Georgia/2/2011/EPI_ISL_189690/PB2/H9N1/2011-04-19
A/black-headed_gull/Republic_of_Georgia/1/2010/EPI_ISL_189723/PB2/H11N1/2010-01-20
A/quail/Lebanon/273/2010/EPI_ISL_93286/PB2/H9N2/2010-08-15
A/duck/Shanghai/120-1/2009/EPI_ISL_224891/PB2/H3N8/2009-07-01
A/duck/Hebei/B1647-1/2011/EPI_ISL_224896/PB2/H3N2/2011-07-01
A/duck/Shanghai/602/2009/EPI_ISL_252870/PB2/H10N8/2009-12-15
A/duck/Guangxi/052/2010/EPI_ISL_273049/PB2/H6N5/2010-02-04
A/aquatic_bird/Korea/w347/2008/EPI_ISL_75195/PB2/H5N2/2008-11-15
A/duck/Henan/S4179/2009/EPI_ISL_206828/PB2/H4N6/2009-12-03
A/duck/Vietnam/LBM1/2011/EPI_ISL_129146/PB2/H4N6/2011-09-20
A/muscovy_duck/Vietnam/LBM14/2011_(H3N2)/EPI_ISL_129147/PB2/H3N2/2011-09-20
A/wild_duck/Korea/CSM42-1/2011/EPI_ISL_156980/PB2/H7N9/2011-03-15
A/wild_duck/Korea/CSM42-34/2011/EPI_ISL_156979/PB2/H7N9/2011-03-15
A/mallard/Alaska/984/2012/EPI_ISL_257272/PB2/H4N6/2012-10-17
A/tundra_swan/Shimane/3211A001/2011/EPI_ISL_144490/PB2/H5N2/2011-11-07
A/wild_waterfowl/Hong_Kong/MPM3375/2011/EPI_ISL_146315/PB2/H7N6/2011-07-01
A/mallard/Korea/1041/2010/EPI_ISL_125925/PB2/H10N8/2010-11-15
A/lesser_whistling-duck/Thailand/CU-W3946/2010/EPI_ISL_110107/PB2/H12N1/2010-01-15
A/lesser_whistling-duck/Thailand/CU-W3941/2010/EPI_ISL_110105/PB2/H12N1/2010-01-15
A/ruddy_shelduck/Mongolia/921C2/2009/EPI_ISL_149895/PB2/H7N1/2009-08-06
A/duck/Hunan/S1166/2010/EPI_ISL_206833/PB2/H4N6/2010-03-03
A/Egyptian_goose/Egypt/05588-NAMRU3/2006/EPI_ISL_120199/PB2/H7N7/2006-04-07
A/mallard/Sweden/5944/2005/EPI_ISL_189464/PB2/H7N7/2005-11-26
A/mallard/Sweden/5994/2005/EPI_ISL_189320/PB2/H7N7/2005-12-06
A/mallard/Sweden/5927/2005/EPI_ISL_189318/PB2/A/2005-11-23
A/mallard/Sweden/95/2005/EPI_ISL_84571/PB2/H7N7/2005-07-01
A/T_Dk/NL-Werkendam/16014159-001/2016/EPI_ISL_287564/PB2/H5N5/2016-11-14
A/goose/Guangdong/k0103/2010/EPI_ISL_139384/PB2/H5N5/2010-01-27
A/goose/Shandong/k1204/2009/EPI_ISL_139383/PB2/H5N5/2009-12-16
A/duck/Eastern_China/031/2009/EPI_ISL_94330/PB2/H5N5/2009-01-15
A/duck/Eastern_China/008/2008/EPI_ISL_94329/PB2/H5N5/2008-12-15
A/duck/Guangdong/wy11/2008/EPI_ISL_152085/PB2/H5N5/2008-06-06
A/duck/Guangdong/wy19/2008/EPI_ISL_152086/PB2/H5N5/2008-06-06
A/duck/Guangdong/wy24/2008/EPI_ISL_152087/PB2/H5N5/2008-06-06
A/duck/Liaoning/LN/2011/EPI_ISL_290070/PB2/H5N5/2011-03-01
A/quail/Jiangsu/k0104/2010/EPI_ISL_139386/PB2/H5N5/2010-01-08
A/duck/HuBei/03/2010/EPI_ISL_130353/PB2/H5N5/2010-03-28
1970
1980
1990
2000
2010
2020

PB1 H5N5-GT2

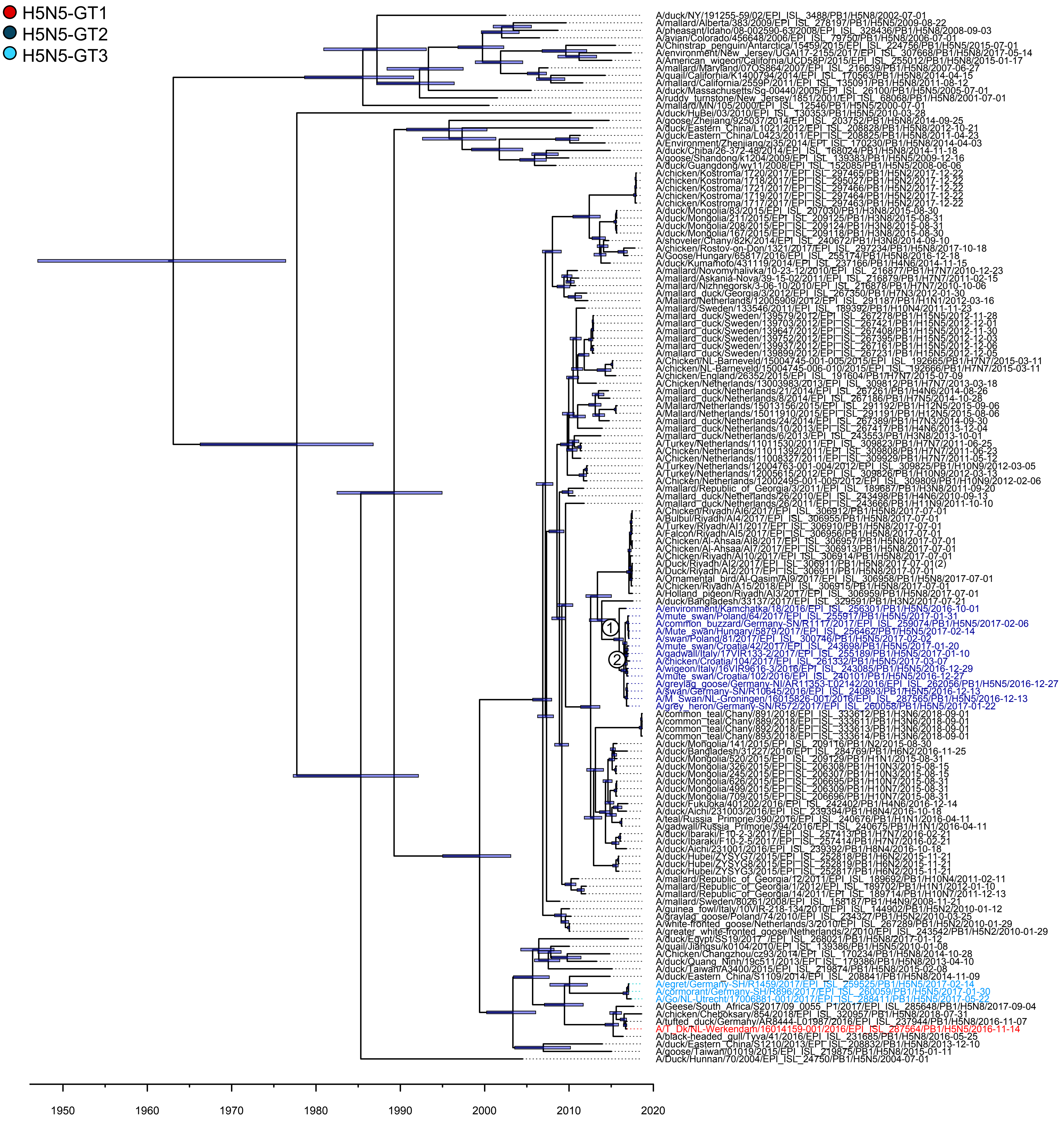

H5N5-GT1
H5N5-GT2
H5N5-GT3
1
2
1950
1960
1970
1980
1990
2000
2010
2020

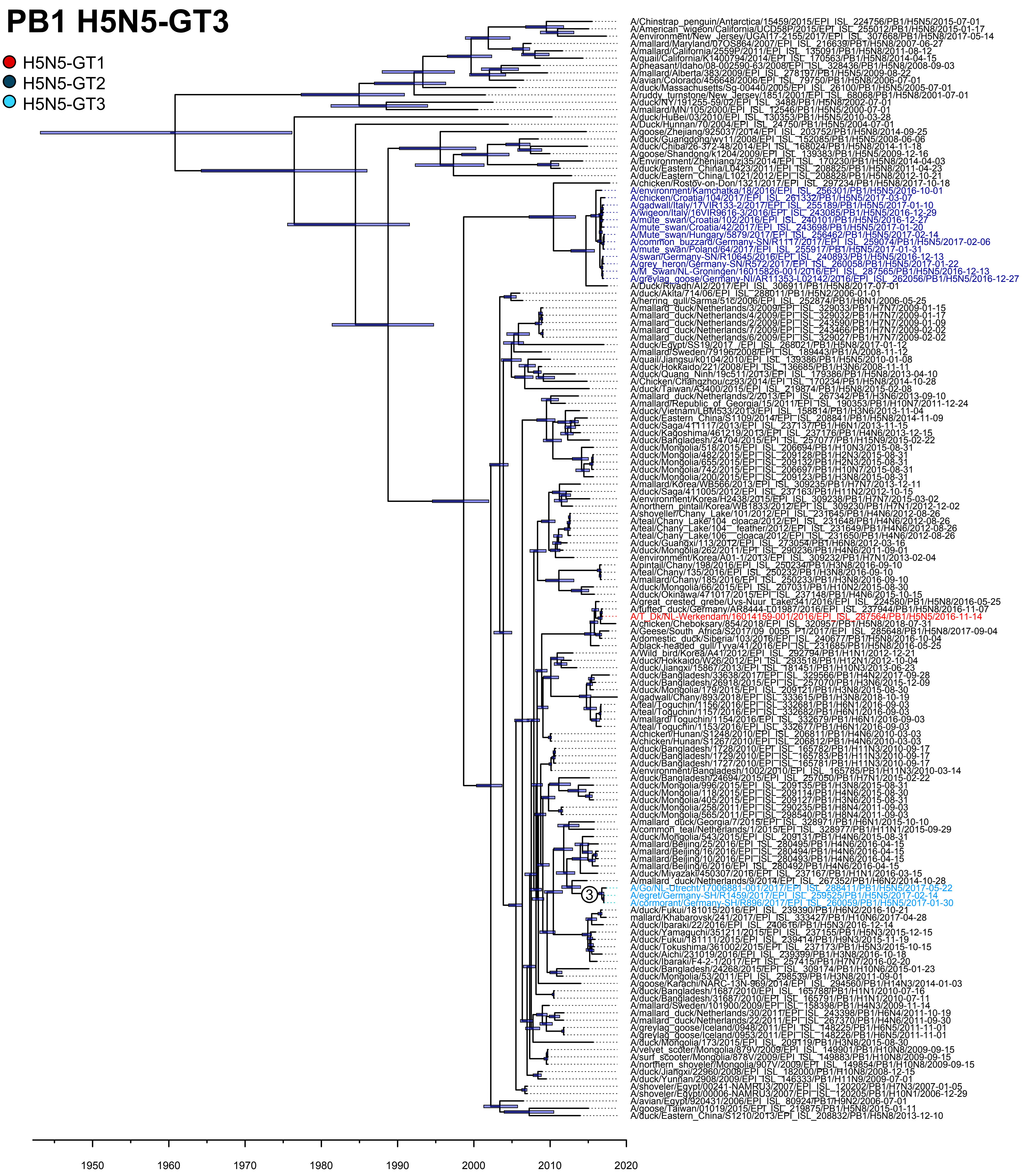
PB1 H5N5-GT3
H5N5-GT1
H5N5-GT2
H5N5-GT3
1950
1960
1970
1980
1990
2000
2010
2020

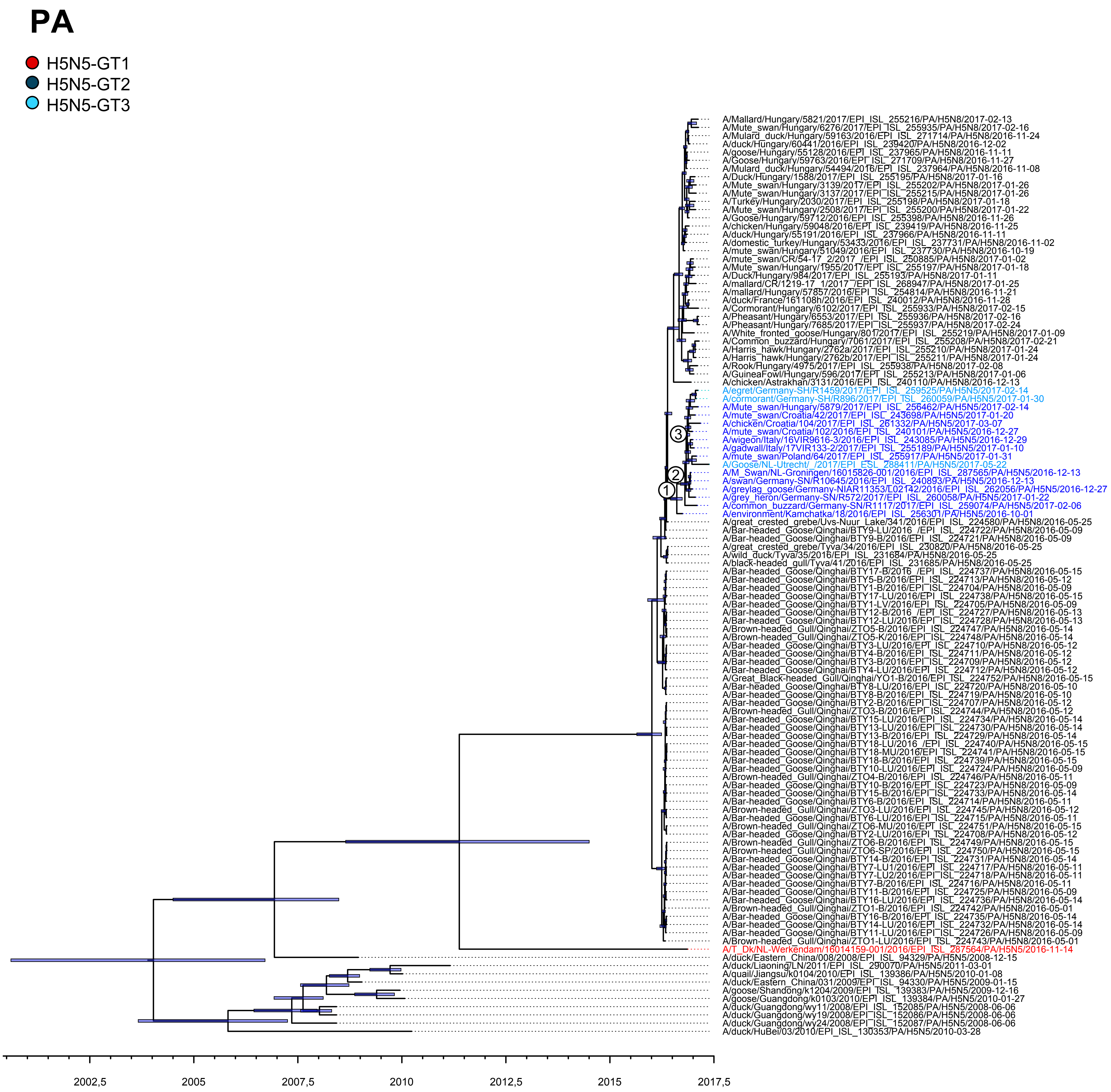

PA
H5N5-GT1
H5N5-GT2
H5N5-GT3
A/Mallard/Hungary/5821/2017/EPI_ISL_255216/PA/H5N8/2017-02-13
A/Mute_swan/Hungary/6276/2017/EPI_ISL_255935/PA/H5N8/2017-02-16
A/Mulard_duck/Hungary/59163/2016/EPI_ISL_271714/PA/H5N8/2016-11-24
A/duck/Hungary/60441/2016/EPI_ISL_239420/PA/H5N8/2016-12-02
A/goose/Hungary/55128/2016/EPI_ISL_237965/PA/H5N8/2016-11-11
A/Goose/Hungary/59763/2016/EPI_ISL_271709/PA/H5N8/2016-11-27
A/Mulard_duck/Hungary/54494/2016/EPI_ISL_237964/PA/H5N8/2016-11-08
A/Duck/Hungary/1588/2017/EPI_ISL_255195/PA/H5N8/2017-01-16
A/Mute_swan/Hungary/3139/2017/EPI_ISL_255202/PA/H5N8/2017-01-26
A/Mute_swan/Hungary/3137/2017/EPI_ISL_255215/PA/H5N8/2017-01-26
A/Turkey/Hungary/2030/2017/EPI_ISL_255198/PA/H5N8/2017-01-18
A/Mute_swan/Hungary/2508/2017/EPI_ISL_255200/PA/H5N8/2017-01-22
A/Goose/Hungary/59712/2016/EPI_ISL_255398/PA/H5N8/2016-11-26
A/chicken/Hungary/59048/2016/EPI_ISL_239419/PA/H5N8/2016-11-25
A/duck/Hungary/55191/2016/EPI_ISL_237966/PA/H5N8/2016-11-11
A/domestic_turkey/Hungary/53433/2016/EPI_ISL_237731/PA/H5N8/2016-11-02
A/mute_swan/Hungary/51049/2016/EPI_ISL_237730/PA/H5N8/2016-10-19
A/mute_swan/CR/54-17_2/2017_/EPI_ISL_250885/PA/H5N8/2017-01-02
A/Mute_swan/Hungary/1955/2017/EPI_ISL_255197/PA/H5N8/2017-01-18
A/Duck/Hungary/984/2017/EPI_ISL_255193/PA/H5N8/2017-01-11
A/mallard/CR/1219-17_1/2017_/EPI_ISL_268947/PA/H5N8/2017-01-25
A/mallard/Hungary/57857/2016/EPI_ISL_254814/PA/H5N8/2016-11-21
A/duck/France/161108h/2016/EPI_ISL_240012/PA/H5N8/2016-11-28
A/Cormorant/Hungary/6102/2017/EPI_ISL_255933/PA/H5N8/2017-02-15
A/Pheasant/Hungary/6553/2017/EPI_ISL_255936/PA/H5N8/2017-02-16
A/Pheasant/Hungary/7685/2017/EPI_ISL_255937/PA/H5N8/2017-02-24
A/White_fronted_goose/Hungary/801/2017/EPI_ISL_255219/PA/H5N8/2017-01-09
A/Common_buzzard/Hungary/7061/2017/EPI_ISL_255208/PA/H5N8/2017-02-21
A/Harris_hawk/Hungary/2762a/2017/EPI_ISL_255210/PA/H5N8/2017-01-24
A/Harris_hawk/Hungary/2762b/2017/EPI_ISL_255211/PA/H5N8/2017-01-24
A/Rook/Hungary/4975/2017/EPI_ISL_255938/PA/H5N8/2017-02-08
A/GuineaFowl/Hungary/596/2017/EPI_ISL_255213/PA/H5N8/2017-01-06
A/chicken/Astrakhan/3131/2016/EPI_ISL_240110/PA/H5N8/2016-12-13
A/egret/Germany-SH/R1459/2017/EPI_ISL_259525/PA/H5N5/2017-02-14
A/cormorant/Germany-SH/R896/2017/EPI_ISL_260059/PA/H5N5/2017-01-30
A/Mute_swan/Hungary/5879/2017/EPI_ISL_256462/PA/H5N5/2017-02-14
A/mute_swan/Croatia/42/2017/EPI_ISL_243698/PA/H5N5/2017-01-20
A/chicken/Croatia/104/2017/EPI_ISL_261332/PA/H5N5/2017-03-07
A/mute_swan/Croatia/102/2016/EPI_ISL_240101/PA/H5N5/2016-12-27
A/wigeon/Italy/16VIR9616-3/2016/EPI_ISL_243085/PA/H5N5/2016-12-29
A/gadwall/Italy/17VIR133-2/2017/EPI_ISL_255189/PA/H5N5/2017-01-10
A/mute_swan/Poland/64/2017/EPI_ISL_255917/PA/H5N5/2017-01-31
A/Goose/NL-Utrecht/_/2017/EPI_ESL_288411/PA/H5N5/2017-05-22
A/M_Swan/NL-Groningen/16015826-001/2016/EPI_ISL_287565/PA/H5N5/2016-12-13
A/swan/Germany-SN/R10645/2016/EPI_ISL_240893/PA/H5N5/2016-12-13
A/greylag_goose/Germany-NIAR11353/L02142/2016/EPI_ISL_262056/PA/H5N5/2016-12-27
A/grey_heron/Germany-SN/R572/2017/EPI_ISL_260058/PA/H5N5/2017-01-22
A/common_buzzard/Germany-SN/R1117/2017/EPI_ISL_259074/PA/H5N5/2017-02-06
A/environment/Kamchatka/18/2016/EPI_ISL_256301/PA/H5N5/2016-10-01
A/great_crested_grebe/Uvs-Nuur_Lake/341/2016/EPI_ISL_224580/PA/H5N8/2016-05-25
A/Bar-headed_Goose/Qinghai/BTY9-LU/2016_/EPI_ISL_224722/PA/H5N8/2016-05-09
A/Bar-headed_Goose/Qinghai/BTY9-B/2016/EPI_ISL_224721/PA/H5N8/2016-05-09
A/great_crested_grebe/Tyva/34/2016/EPI_ISL_230820/PA/H5N8/2016-05-25
A/wild_duck/Tyva/35/2016/EPI_ISL_231684/PA/H5N8/2016-05-25
A/black-headed_gull/Tyva/41/2016/EPI_ISL_231685/PA/H5N8/2016-05-25
A/Bar-headed_Goose/Qinghai/BTY17-B/2016_/EPI_ISL_224737/PA/H5N8/2016-05-15
A/Bar-headed_Goose/Qinghai/BTY5-B/2016/EPI_ISL_224713/PA/H5N8/2016-05-12
A/Bar-headed_Goose/Qinghai/BTY1-B/2016/EPI_ISL_224704/PA/H5N8/2016-05-09
A/Bar-headed_Goose/Qinghai/BTY17-LU/2016/EPI_ISL_224738/PA/H5N8/2016-05-15
A/Bar-headed_Goose/Qinghai/BTY1-LV/2016/EPI_ISL_224705/PA/H5N8/2016-05-09
A/Bar-headed_Goose/Qinghai/BTY12-B/2016_/EPI_ISL_224727/PA/H5N8/2016-05-13
A/Bar-headed_Goose/Qinghai/BTY12-LU/2016/EPI_ISL_224728/PA/H5N8/2016-05-13
A/Brown-headed_Gull/Qinghai/ZTO5-B/2016/EPI_ISL_224747/PA/H5N8/2016-05-14
A/Brown-headed_Gull/Qinghai/ZTO5-K/2016/EPI_ISL_224748/PA/H5N8/2016-05-14
A/Bar-headed_Goose/Qinghai/BTY3-LU/2016/EPI_ISL_224710/PA/H5N8/2016-05-12
A/Bar-headed_Goose/Qinghai/BTY4-B/2016/EPI_ISL_224711/PA/H5N8/2016-05-12
A/Bar-headed_Goose/Qinghai/BTY3-B/2016/EPI_ISL_224709/PA/H5N8/2016-05-12
A/Bar-headed_Goose/Qinghai/BTY4-LU/2016/EPI_ISL_224712/PA/H5N8/2016-05-12
A/Great_Black-headed_Gull/Qinghai/YO1-B/2016/EPI_ISL_224752/PA/H5N8/2016-05-15
A/Bar-headed_Goose/Qinghai/BTY8-LU/2016/EPI_ISL_224720/PA/H5N8/2016-05-10
A/Bar-headed_Goose/Qinghai/BTY8-B/2016/EPI_ISL_224719/PA/H5N8/2016-05-10
A/Bar-headed_Goose/Qinghai/BTY2-B/2016/EPI_ISL_224707/PA/H5N8/2016-05-12
A/Brown-headed_Gull/Qinghai/ZTO3-B/2016/EPI_ISL_224744/PA/H5N8/2016-05-12
A/Bar-headed_Goose/Qinghai/BTY15-LU/2016/EPI_ISL_224734/PA/H5N8/2016-05-14
A/Bar-headed_Goose/Qinghai/BTY13-LU/2016/EPI_ISL_224730/PA/H5N8/2016-05-14
A/Bar-headed_Goose/Qinghai/BTY13-B/2016/EPI_ISL_224729/PA/H5N8/2016-05-14
A/Bar-headed_Goose/Qinghai/BTY18-LU/2016_/EPI_ISL_224740/PA/H5N8/2016-05-15
A/Bar-headed_Goose/Qinghai/BTY18-MU/2016/EPI_ISL_224741/PA/H5N8/2016-05-15
A/Bar-headed_Goose/Qinghai/BTY18-B/2016/EPI_ISL_224739/PA/H5N8/2016-05-15
A/Bar-headed_Goose/Qinghai/BTY10-LU/2016/EPI_ISL_224724/PA/H5N8/2016-05-09
A/Brown-headed_Gull/Qinghai/ZTO4-B/2016/EPI_ISL_224746/PA/H5N8/2016-05-11
A/Bar-headed_Goose/Qinghai/BTY10-B/2016/EPI_ISL_224723/PA/H5N8/2016-05-09
A/Bar-headed_Goose/Qinghai/BTY15-B/2016/EPI_ISL_224733/PA/H5N8/2016-05-14
A/Bar-headed_Goose/Qinghai/BTY6-B/2016/EPI_ISL_224714/PA/H5N8/2016-05-11
A/Brown-headed_Gull/Qinghai/ZTO3-LU/2016/EPI_ISL_224745/PA/H5N8/2016-05-12
A/Bar-headed_Goose/Qinghai/BTY6-LU/2016/EPI_ISL_224715/PA/H5N8/2016-05-11
A/Brown-headed_Gull/Qinghai/ZTO6-MU/2016/EPI_ISL_224751/PA/H5N8/2016-05-15
A/Bar-headed_Goose/Qinghai/BTY2-LU/2016/EPI_ISL_224708/PA/H5N8/2016-05-12
A/Brown-headed_Gull/Qinghai/ZTO6-B/2016/EPI_ISL_224749/PA/H5N8/2016-05-15
A/Brown-headed_Gull/Qinghai/ZTO6-SP/2016/EPI_ISL_224750/PA/H5N8/2016-05-15
A/Bar-headed_Goose/Qinghai/BTY14-B/2016/EPI_ISL_224731/PA/H5N8/2016-05-14
A/Bar-headed_Goose/Qinghai/BTY7-LU1/2016/EPI_ISL_224717/PA/H5N8/2016-05-11
A/Bar-headed_Goose/Qinghai/BTY7-LU2/2016/EPI_ISL_224718/PA/H5N8/2016-05-11
A/Bar-headed_Goose/Qinghai/BTY7-B/2016/EPI_ISL_224716/PA/H5N8/2016-05-11
A/Bar-headed_Goose/Qinghai/BTY11-B/2016/EPI_ISL_224725/PA/H5N8/2016-05-09
A/Bar-headed_Goose/Qinghai/BTY16-LU/2016/EPI_ISL_224736/PA/H5N8/2016-05-14
A/Brown-headed_Gull/Qinghai/ZTO1-B/2016/EPI_ISL_224742/PA/H5N8/2016-05-01
A/Bar-headed_Goose/Qinghai/BTY16-B/2016/EPI_ISL_224735/PA/H5N8/2016-05-14
A/Bar-headed_Goose/Qinghai/BTY14-LU/2016/EPI_ISL_224732/PA/H5N8/2016-05-14
A/Bar-headed_Goose/Qinghai/BTY11-LU/2016/EPI_ISL_224726/PA/H5N8/2016-05-09
A/Brown-headed_Gull/Qinghai/ZTO1-LU/2016/EPI_ISL_224743/PA/H5N8/2016-05-01
A/T_Dk/NL-Werkendam/16014159-001/2016/EPI_ISL_287564/PA/H5N5/2016-11-14
A/duck/Eastern_China/008/2008/EPI_ISL_94329/PA/H5N5/2008-12-15
A/duck/Liaoning/LN/2011/EPI_ISL_290070/PA/H5N5/2011-03-01
A/quail/Jiangsu/k0104/2010/EPI_ISL_139386/PA/H5N5/2010-01-08
A/duck/Eastern_China/031/2009/EPI_ISL_94330/PA/H5N5/2009-01-15
A/goose/Shandong/k1204/2009/EPI_ISL_139383/PA/H5N5/2009-12-16
A/goose/Guangdong/k0103/2010/EPI_ISL_139384/PA/H5N5/2010-01-27
A/duck/Guangdong/wy11/2008/EPI_ISL_152085/PA/H5N5/2008-06-06
A/duck/Guangdong/wy19/2008/EPI_ISL_152086/PA/H5N5/2008-06-06
A/duck/Guangdong/wy24/2008/EPI_ISL_152087/PA/H5N5/2008-06-06
A/duck/HuBei/03/2010/EPI_ISL_130353/PA/H5N5/2010-03-28
1
2
3
2002,5
2005
2007,5
2010
2012,5
2015
2017,5

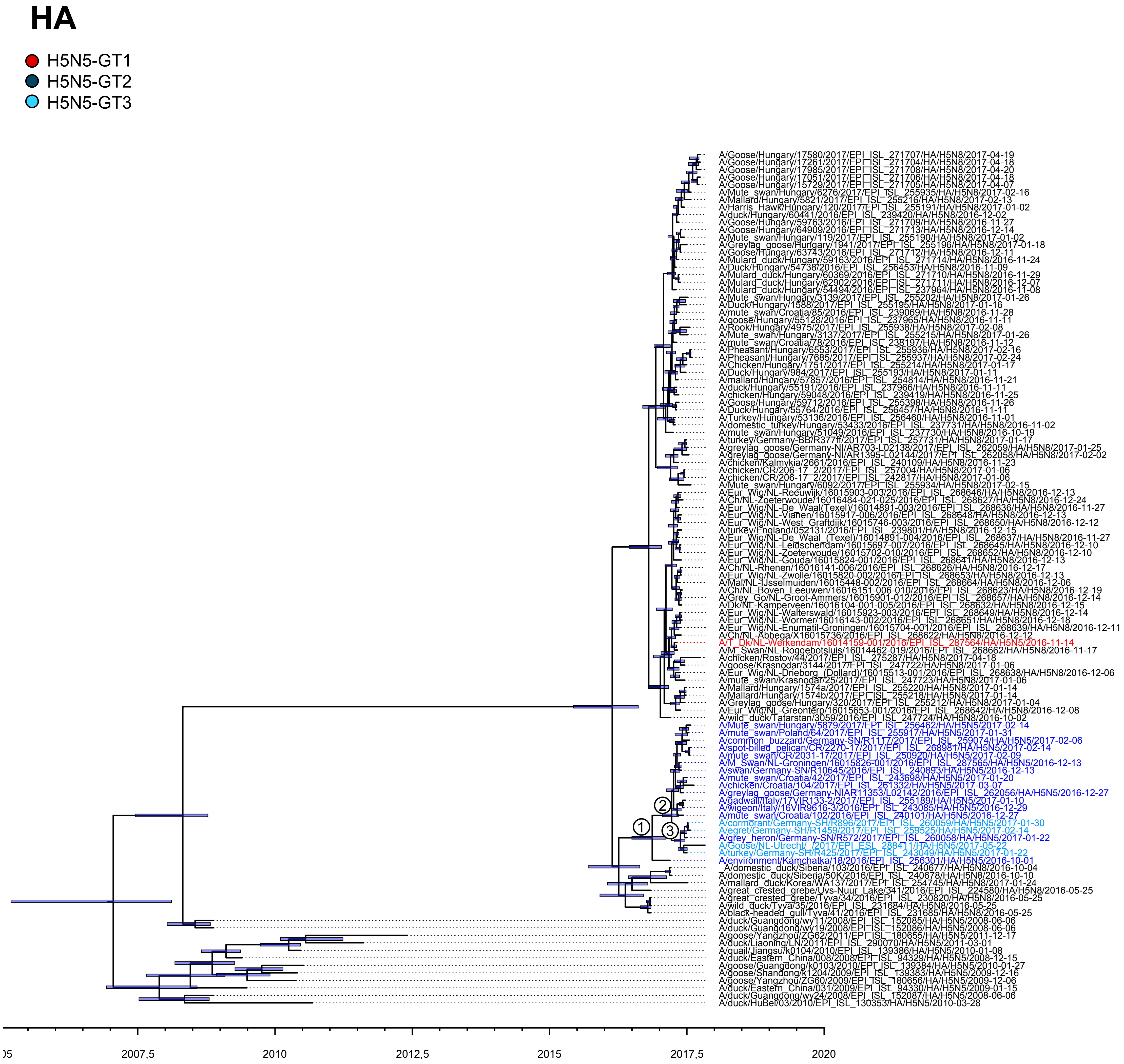

HA
H5N5-GT1
H5N5-GT2
H5N5-GT3
A/Goose/Hungary/17580/2017/EPI_ISL_271707/HA/H5N8/2017-04-19
A/Goose/Hungary/17261/2017/EPI_ISL_271704/HA/H5N8/2017-04-18
A/Goose/Hungary/17985/2017/EPI_ISL_271708/HA/H5N8/2017-04-20
A/Goose/Hungary/17051/2017/EPI_ISL_271706/HA/H5N8/2017-04-18
A/Goose/Hungary/15729/2017/EPI_ISL_271705/HA/H5N8/2017-04-07
A/Mute_swan/Hungary/6276/2017/EPI_ISL_255935/HA/H5N8/2017-02-16
A/Mallard/Hungary/5821/2017/EPI_ISL_255216/HA/H5N8/2017-02-13
A/Harris_Hawk/Hungary/120/2017/EPI_ISL_255191/HA/H5N8/2017-01-02
A/duck/Hungary/60441/2016/EPI_ISL_239420/HA/H5N8/2016-12-02
A/Goose/Hungary/59763/2016/EPI_ISL_271709/HA/H5N8/2016-11-27
A/Goose/Hungary/64909/2016/EPI_ISL_271713/HA/H5N8/2016-12-14
A/Mute_swan/Hungary/119/2017/EPI_ISL_255190/HA/H5N8/2017-01-02
A/Greylag_goose/Hungary/1941/2017/EPI_ISL_255196/HA/H5N8/2017-01-18
A/Goose/Hungary/63743/2016/EPI_ISL_271712/HA/H5N8/2016-12-11
A/Mulard_duck/Hungary/59163/2016/EPI_ISL_271714/HA/H5N8/2016-11-24
A/Duck/Hungary/54738/2016/EPI_ISL_256453/HA/H5N8/2016-11-09
A/Mulard_duck/Hungary/60369/2016/EPI_ISL_271710/HA/H5N8/2016-11-29
A/Mulard_duck/Hungary/62902/2016/EPI_ISL_271711/HA/H5N8/2016-12-07
A/Mulard_duck/Hungary/54494/2016/EPI_ISL_237964/HA/H5N8/2016-11-08
A/Mute_swan/Hungary/3139/2017/EPI_ISL_255202/HA/H5N8/2017-01-26
A/Duck/Hungary/1588/2017/EPI_ISL_255195/HA/H5N8/2017-01-16
A/mute_swan/Croatia/85/2016/EPI_ISL_239069/HA/H5N8/2016-11-28
A/goose/Hungary/55128/2016/EPI_ISL_237965/HA/H5N8/2016-11-11
A/Rook/Hungary/4975/2017/EPI_ISL_255938/HA/H5N8/2017-02-08
A/Mute_swan/Hungary/3137/2017/EPI_ISL_255215/HA/H5N8/2017-01-26
A/mute_swan/Croatia/78/2016/EPI_ISL_238197/HA/H5N8/2016-11-12
A/Pheasant/Hungary/6553/2017/EPI_ISL_255936/HA/H5N8/2017-02-16
A/Pheasant/Hungary/7685/2017/EPI_ISL_255937/HA/H5N8/2017-02-24
A/Chicken/Hungary/1751/2017/EPI_ISL_255214/HA/H5N8/2017-01-17
A/Duck/Hungary/984/2017/EPI_ISL_255193/HA/H5N8/2017-01-11
A/mallard/Hungary/57857/2016/EPI_ISL_254814/HA/H5N8/2016-11-21
A/duck/Hungary/55191/2016/EPI_ISL_237966/HA/H5N8/2016-11-11
A/chicken/Hungary/59048/2016/EPI_ISL_239419/HA/H5N8/2016-11-25
A/Goose/Hungary/59712/2016/EPI_ISL_255398/HA/H5N8/2016-11-26
A/Duck/Hungary/55764/2016/EPI_ISL_256457/HA/H5N8/2016-11-11
A/Turkey/Hungary/53136/2016/EPI_ISL_256460/HA/H5N8/2016-11-01
A/domestic_turkey/Hungary/53433/2016/EPI_ISL_237731/HA/H5N8/2016-11-02
A/mute_swan/Hungary/51049/2016/EPI_ISL_237730/HA/H5N8/2016-10-19
A/turkey/Germany-BB/R377ff/2017/EPI_ISL_257731/HA/H5N8/2017-01-17
A/greylag_goose/Germany-NI/AR703-L02138/2017/EPI_ISL_262059/HA/H5N8/2017-01-25
A/greylag_goose/Germany-NI/AR1395-L02144/2017/EPI_ISL_262058/HA/H5N8/2017-02-02
A/chicken/Kalmykia/2661/2016/EPI_ISL_240109/HA/H5N8/2016-11-23
A/chicken/CR/206-17_2/2017/EPI_ISL_257004/HA/H5N8/2017-01-06
A/chicken/CR/206-17_2/2017/EPI_ISL_242817/HA/H5N8/2017-01-06
A/Mute_swan/Hungary/6092/2017/EPI_ISL_255934/HA/H5N8/2017-02-15
A/Eur_Wig/NL-Reeuwijk/16015903-003/2016/EPI_ISL_268646/HA/H5N8/2016-12-13
A/Ch/NL-Zoeterwoude/16016484-021-025/2016/EPI_ISL_268627/HA/H5N8/2016-12-24
A/Eur_Wig/NL-De_Waal(Texel)/16014891-003/2016/EPI_ISL_268636/HA/H5N8/2016-11-27
A/Eur_Wig/NL-Vianen/16015917-006/2016/EPI_ISL_268648/HA/H5N8/2016-12-13
A/Eur_Wig/NL-West_Graftdijk/16015746-003/2016/EPI_ISL_268650/HA/H5N8/2016-12-12
A/turkey/England/052131/2016/EPI_ISL_239801/HA/H5N8/2016-12-15
A/Eur_Wig/NL-De_Waal_(Texel)/16014891-004/2016/EPI_ISL_268637/HA/H5N8/2016-11-27
A/Eur_Wig/NL-Leidschendam/16015697-007/2016/EPI_ISL_268645/HA/H5N8/2016-12-10
A/Eur_Wig/NL-Zoeterwoude/16015702-010/2016/EPI_ISL_268652/HA/H5N8/2016-12-10
A/Eur_Wig/NL-Gouda/16015824-001/2016/EPI_ISL_268641/HA/H5N8/2016-12-13
A/Ch/NL-Rhenen/16016141-006/2016/EPI_ISL_268626/HA/H5N8/2016-12-17
A/Eur_Wig/NL-Zwolle/16015820-002/2016/EPI_ISL_268653/HA/H5N8/2016-12-13
A/Mal/NL-IJsselmuiden/16015448-002/2016/EPI_ISL_268664/HA/H5N8/2016-12-06
A/Ch/NL-Boven_Leeuwen/16016151-006-010/2016/EPI_ISL_268623/HA/H5N8/2016-12-19
A/Grey_Go/NL-Groot-Ammers/16015901-012/2016/EPI_ISL_268657/HA/H5N8/2016-12-14
A/Dk/NL-Kamperveen/16016104-001-005/2016/EPI_ISL_268632/HA/H5N8/2016-12-15
A/Eur_Wig/NL-Walterswald/16015923-003/2016/EPI_ISL_268649/HA/H5N8/2016-12-14
A/Eur_Wig/NL-Wormer/16016143-002/2016/EPI_ISL_268651/HA/H5N8/2016-12-18
A/Eur_Wig/NL-Enumatil-Groningen/16015704-001/2016/EPI_ISL_268639/HA/H5N8/2016-12-11
A/Ch/NL-Abbega/X16015736/2016/EPI_ISL_268622/HA/H5N8/2016-12-12
A/T_Dk/NL-Werkendam/16014159-001/2016/EPI_ISL_287564/HA/H5N5/2016-11-14
A/M_Swan/NL-Roggebotsluis/16014462-019/2016/EPI_ISL_268662/HA/H5N8/2016-11-17
A/chicken/Rostov/44/2017/EPI_ISL_275287/HA/H5N8/2017-04-18
A/goose/Krasnodar/3144/2017/EPI_ISL_247722/HA/H5N8/2017-01-06
A/Eur_Wig/NL-Drieborg_(Dollard)/16015513-001/2016/EPI_ISL_268638/HA/H5N8/2016-12-06
A/mute_swan/Krasnodar/25/2017/EPI_ISL_247723/HA/H5N8/2017-01-06
A/Mallard/Hungary/1574a/2017/EPI_ISL_255220/HA/H5N8/2017-01-14
A/Mallard/Hungary/1574b/2017/EPI_ISL_255218/HA/H5N8/2017-01-14
A/Greylag_goose/Hungary/320/2017/EPI_ISL_255212/HA/H5N8/2017-01-04
A/Eur_Wig/NL-Greonterp/16015653-001/2016/EPI_ISL_268642/HA/H5N8/2016-12-08
A/wild_duck/Tatarstan/3059/2016/EPI_ISL_247724/HA/H5N8/2016-10-02
A/Mute_swan/Hungary/5879/2017/EPI_ISL_256462/HA/H5N5/2017-02-14
A/mute_swan/Poland/64/2017/EPI_ISL_255917/HA/H5N5/2017-01-31
A/common_buzzard/Germany-SN/R1117/2017/EPI_ISL_259074/HA/H5N5/2017-02-06
A/spot-billed_pelican/CR/2270-17/2017/EPI_ISL_268981/HA/H5N5/2017-02-14
A/mute_swan/CR/2031-17/2017/EPI_ISL_250920/HA/H5N5/2017-02-09
A/M_Swan/NL-Groningen/16015826-011/2016/EPI_ISL_287565/HA/H5N5/2016-12-13
A/swan/Germany-SN/R10645/2016/EPI_ISL_240893/HA/H5N5/2016-12-13
A/mute_swan/Croatia/42/2017/EPI_ISL_243698/HA/H5N5/2017-01-20
A/chicken/Croatia/104/2017/EPI_ISL_261332/HA/H5N5/2017-03-07
A/greylag_goose/Germany-NI/AR11353/L02142/2016/EPI_ISL_262056/HA/H5N5/2016-12-27
A/gadwall/Italy/17VIR133-2/2017/EPI_ISL_255189/HA/H5N5/2017-01-10
A/wigeon/Italy/16VIR9616-3/2016/EPI_ISL_243085/HA/H5N5/2016-12-29
A/mute_swan/Croatia/102/2016/EPI_ISL_240101/HA/H5N5/2016-12-27
A/cormorant/Germany-SH/R896/2017/EPI_ISL_260059/HA/H5N5/2017-01-30
A/egret/Germany-SH/R1459/2017/EPI_ISL_259525/HA/H5N5/2017-02-14
A/grey_heron/Germany-SN/R572/2017/EPI_ISL_260058/HA/H5N5/2017-01-22
A/Goose/NL-Utrecht/_/2017/EPI_ISL_288411/HA/H5N5/2017-05-22
A/turkey/Germany-SH/R425/2017/EPI_ISL_243049/HA/H5N5/2017-01-22
A/environment/Kamchatka/18/2016/EPI_ISL_256301/HA/H5N5/2016-10-01
A/domestic_duck/Siberia/103/2016/EPI_ISL_240677/HA/H5N8/2016-10-04
A/domestic_duck/Siberia/50K/2016/EPI_ISL_240678/HA/H5N8/2016-10-10
A/mallard_duck/Korea/WA137/2017/EPI_ISL_254745/HA/H5N8/2017-01-24
A/great_crested_grebe/Uvs-Nuur_Lake/341/2016/EPI_ISL_224580/HA/H5N8/2016-05-25
A/great_crested_grebe/Tyva/34/2016/EPI_ISL_230820/HA/H5N8/2016-05-25
A/wild_duck/Tyva/35/2016/EPI_ISL_231684/HA/H5N8/2016-05-25
A/black-headed_gull/Tyva/41/2016/EPI_ISL_231685/HA/H5N8/2016-05-25
A/duck/Guangdong/wy11/2008/EPI_ISL_152085/HA/H5N5/2008-06-06
A/duck/Guangdong/wy19/2008/EPI_ISL_152086/HA/H5N5/2008-06-06
A/goose/Yangzhou/ZG62/2011/EPI_ISL_180655/HA/H5N5/2011-12-17
A/duck/Liaoning/LN/2011/EPI_ISL_290070/HA/H5N5/2011-03-01
A/quail/Jiangsu/k0104/2010/EPI_ISL_139386/HA/H5N5/2010-01-08
A/duck/Eastern_China/008/2008/EPI_ISL_94329/HA/H5N5/2008-12-15
A/goose/Guangdong/k0103/2010/EPI_ISL_139384/HA/H5N5/2010-01-27
A/goose/Shandong/k1204/2009/EPI_ISL_139383/HA/H5N5/2009-12-16
A/goose/Yangzhou/ZG60/2009/EPI_ISL_180656/HA/H5N5/2009-12-06
A/duck/Eastern_China/031/2009/EPI_ISL_94330/HA/H5N5/2009-01-15
A/duck/Guangdong/wy24/2008/EPI_ISL_152087/HA/H5N5/2008-06-06
A/duck/HuBei/03/2010/EPI_ISL_130353/HA/H5N5/2010-03-28
1
2
3
05
2007,5
2010
2012,5
2015
2017,5
2020

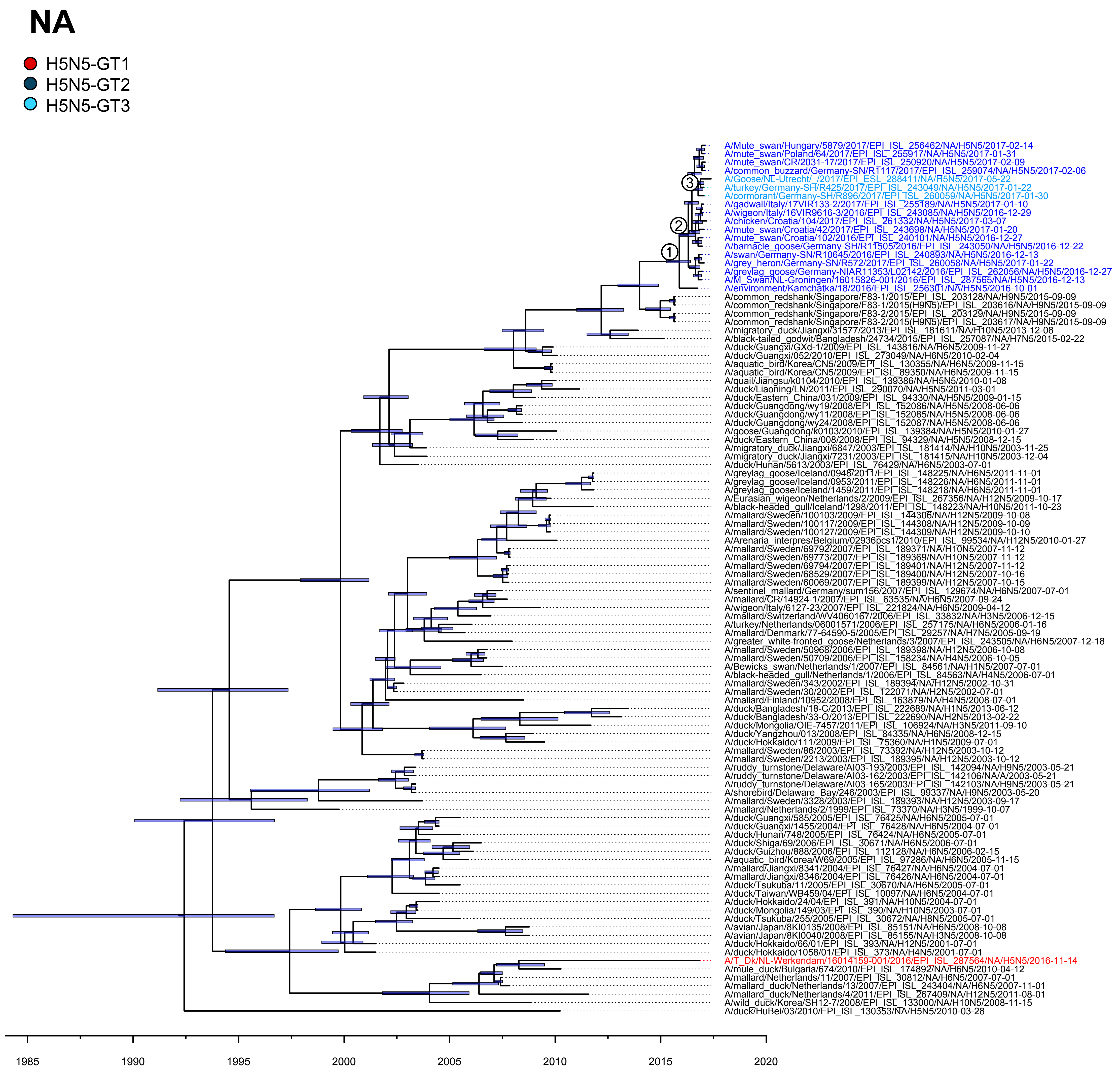
NA
H5N5-GT1
H5N5-GT2
H5N5-GT3
A/Mute_swan/Hungary/5879/2017/EPI_ISL_256462/NA/H5N5/2017-02-14
A/mute_swan/Poland/64/2017/EPI_ISL_255917/NA/H5N5/2017-01-31
A/mute_swan/CR/2031-17/2017/EPI_ISL_250920/NA/H5N5/2017-02-09
A/common_buzzard/Germany-SN/R1117/2017/EPI_ISL_259074/NA/H5N5/2017-02-06
A/Goose/NL-Utrecht/_/2017/EPI_ESL_288411/NA/H5N5/2017-05-22
A/turkey/Germany-SH/R425/2017/EPI_ISL_243049/NA/H5N5/2017-01-22
A/cormorant/Germany-SH/R896/2017/EPI_ISL_260059/NA/H5N5/2017-01-30
A/gadwall/Italy/17VIR133-2/2017/EPI_ISL_255189/NA/H5N5/2017-01-10
A/wigeon/Italy/16VIR9616-3/2016/EPI_ISL_243085/NA/H5N5/2016-12-29
A/chicken/Croatia/104/2017/EPI_ISL_261332/NA/H5N5/2017-03-07
A/mute_swan/Croatia/42/2017/EPI_ISL_243698/NA/H5N5/2017-01-20
A/mute_swan/Croatia/102/2016/EPI_ISL_240101/NA/H5N5/2016-12-27
A/barnacle_goose/Germany-SH/R11505/2016/EPI_ISL_243050/NA/H5N5/2016-12-22
A/swan/Germany-SN/R10645/2016/EPI_ISL_240893/NA/H5N5/2016-12-13
A/grey_heron/Germany-SN/R572/2017/EPI_ISL_260058/NA/H5N5/2017-01-22
A/greylag_goose/Germany-NIAR11353/L02142/2016/EPI_ISL_262056/NA/H5N5/2016-12-27
A/M_Swan/NL-Groningen/16015826-001/2016/EPI_ISL_287565/NA/H5N5/2016-12-13
A/environment/Kamchatka/18/2016/EPI_ISL_256301/NA/H5N5/2016-10-01
A/common_redshank/Singapore/F83-1/2015/EPI_ISL_203128/NA/H9N5/2015-09-09
A/common_redshank/Singapore/F83-1/2015(H9N5)/EPI_ISL_203616/NA/H9N5/2015-09-09
A/common_redshank/Singapore/F83-2/2015/EPI_ISL_203129/NA/H9N5/2015-09-09
A/common_redshank/Singapore/F83-2/2015(H9N5)/EPI_ISL_203617/NA/H9N5/2015-09-09
A/migratory_duck/Jiangxi/31577/2013/EPI_ISL_181611/NA/H10N5/2013-12-08
A/black-tailed_godwit/Bangladesh/24734/2015/EPI_ISL_257087/NA/H7N5/2015-02-22
A/duck/Guangxi/GXd-1/2009/EPI_ISL_143816/NA/H6N5/2009-11-27
A/duck/Guangxi/052/2010/EPI_ISL_273049/NA/H6N5/2010-02-04
A/aquatic_bird/Korea/CN5/2009/EPI_ISL_130355/NA/H6N5/2009-11-15
A/aquatic_bird/Korea/CN5/2009/EPI_ISL_89350/NA/H6N5/2009-11-15
A/quail/Jiangsu/k0104/2010/EPI_ISL_139386/NA/H5N5/2010-01-08
A/duck/Liaoning/LN/2011/EPI_ISL_290070/NA/H5N5/2011-03-01
A/duck/Eastern_China/031/2009/EPI_ISL_94330/NA/H5N5/2009-01-15
A/duck/Guangdong/wy19/2008/EPI_ISL_152086/NA/H5N5/2008-06-06
A/duck/Guangdong/wy11/2008/EPI_ISL_152085/NA/H5N5/2008-06-06
A/duck/Guangdong/wy24/2008/EPI_ISL_152087/NA/H5N5/2008-06-06
A/goose/Guangdong/k0103/2010/EPI_ISL_139384/NA/H5N5/2010-01-27
A/duck/Eastern_China/008/2008/EPI_ISL_94329/NA/H5N5/2008-12-15
A/migratory_duck/Jiangxi/6847/2003/EPI_ISL_181414/NA/H10N5/2003-11-25
A/migratory_duck/Jiangxi/7231/2003/EPI_ISL_181415/NA/H10N5/2003-12-04
A/duck/Hunan/5613/2003/EPI_ISL_76429/NA/H6N5/2003-07-01
A/greylag_goose/Iceland/0948/2011/EPI_ISL_148225/NA/H6N5/2011-11-01
A/greylag_goose/Iceland/0953/2011/EPI_ISL_148226/NA/H6N5/2011-11-01
A/greylag_goose/Iceland/1459/2011/EPI_ISL_148218/NA/H6N5/2011-11-01
A/Eurasian_wigeon/Netherlands/2/2009/EPI_ISL_267356/NA/H12N5/2009-10-17
A/black-headed_gull/Iceland/1298/2011/EPI_ISL_148223/NA/H10N5/2011-10-23
A/mallard/Sweden/100103/2009/EPI_ISL_144306/NA/H12N5/2009-10-08
A/mallard/Sweden/100117/2009/EPI_ISL_144308/NA/H12N5/2009-10-09
A/mallard/Sweden/100127/2009/EPI_ISL_144309/NA/H12N5/2009-10-10
A/Arenaria_interpres/Belgium/02936pcs1/2010/EPI_ISL_99534/NA/H12N5/2010-01-27
A/mallard/Sweden/69792/2007/EPI_ISL_189371/NA/H10N5/2007-11-12
A/mallard/Sweden/69773/2007/EPI_ISL_189369/NA/H10N5/2007-11-12
A/mallard/Sweden/69794/2007/EPI_ISL_189401/NA/H12N5/2007-11-12
A/mallard/Sweden/68529/2007/EPI_ISL_189400/NA/H12N5/2007-10-16
A/mallard/Sweden/60069/2007/EPI_ISL_189399/NA/H12N5/2007-10-15
A/sentinel_mallard/Germany/sum156/2007/EPI_ISL_129674/NA/H6N5/2007-07-01
A/mallard/CR/14924-1/2007/EPI_ISL_63535/NA/H6N5/2007-09-24
A/wigeon/Italy/6127-23/2007/EPI_ISL_221824/NA/H6N5/2009-04-12
A/mallard/Switzerland/WV4060167/2006/EPI_ISL_33832/NA/H3N5/2006-12-15
A/turkey/Netherlands/06001571/2006/EPI_ISL_257175/NA/H6N5/2006-01-16
A/mallard/Denmark/77-64590-5/2005/EPI_ISL_29257/NA/H7N5/2005-09-19
A/greater_white-fronted_goose/Netherlands/3/2007/EPI_ISL_243505/NA/H6N5/2007-12-18
A/mallard/Sweden/50968/2006/EPI_ISL_189398/NA/H12N5/2006-10-08
A/mallard/Sweden/50709/2006/EPI_ISL_158234/NA/H4N5/2006-10-05
A/Bewicks_swan/Netherlands/1/2007/EPI_ISL_84561/NA/H1N5/2007-07-01
A/black-headed_gull/Netherlands/1/2006/EPI_ISL_84563/NA/H4N5/2006-07-01
A/mallard/Sweden/343/2002/EPI_ISL_189394/NA/H12N5/2002-10-31
A/mallard/Sweden/30/2002/EPI_ISL_122071/NA/H2N5/2002-07-01
A/mallard/Finland/10952/2008/EPI_ISL_163879/NA/H4N5/2008-07-01
A/duck/Bangladesh/18-C/2013/EPI_ISL_222689/NA/H1N5/2013-06-12
A/duck/Bangladesh/33-O/2013/EPI_ISL_222690/NA/H2N5/2013-02-22
A/duck/Mongolia/OIE-7457/2011/EPI_ISL_106924/NA/H3N5/2011-09-10
A/duck/Yangzhou/013/2008/EPI_ISL_84335/NA/H6N5/2008-12-15
A/duck/Hokkaido/111/2009/EPI_ISL_75360/NA/H1N5/2009-07-01
A/mallard/Sweden/86/2003/EPI_ISL_73392/NA/H12N5/2003-10-12
A/mallard/Sweden/2213/2003/EPI_ISL_189395/NA/H12N5/2003-10-12
A/ruddy_turnstone/Delaware/AI03-193/2003/EPI_ISL_142094/NA/H9N5/2003-05-21
A/ruddy_turnstone/Delaware/AI03-162/2003/EPI_ISL_142106/NA/A/2003-05-21
A/ruddy_turnstone/Delaware/AI03-165/2003/EPI_ISL_142103/NA/H9N5/2003-05-21
A/shorebird/Delaware_Bay/246/2003/EPI_ISL_99337/NA/H9N5/2003-05-20
A/mallard/Sweden/3328/2003/EPI_ISL_189393/NA/H12N5/2003-09-17
A/mallard/Netherlands/2/1999/EPI_ISL_73370/NA/H3N5/1999-10-07
A/duck/Guangxi/585/2005/EPI_ISL_76425/NA/H6N5/2005-07-01
A/duck/Guangxi/1455/2004/EPI_ISL_76428/NA/H6N5/2004-07-01
A/duck/Hunan/748/2005/EPI_ISL_76424/NA/H6N5/2005-07-01
A/duck/Shiga/69/2006/EPI_ISL_30671/NA/H6N5/2006-07-01
A/duck/Guizhou/888/2006/EPI_ISL_112128/NA/H6N5/2006-02-15
A/aquatic_bird/Korea/W69/2005/EPI_ISL_97286/NA/H6N5/2005-11-15
A/mallard/Jiangxi/8341/2004/EPI_ISL_76427/NA/H6N5/2004-07-01
A/mallard/Jiangxi/8346/2004/EPI_ISL_76426/NA/H6N5/2004-07-01
A/duck/Tsukuba/11/2005/EPI_ISL_30670/NA/H6N5/2005-07-01
A/duck/Taiwan/WB459/04/EPI_ISL_10097/NA/H6N5/2004-07-01
A/duck/Hokkaido/24/04/EPI_ISL_391/NA/H10N5/2004-07-01
A/duck/Mongolia/149/03/EPI_ISL_390/NA/H10N5/2003-07-01
A/duck/Tsukuba/255/2005/EPI_ISL_30672/NA/H8N5/2005-07-01
A/avian/Japan/8KI0135/2008/EPI_ISL_85151/NA/H6N5/2008-10-08
A/avian/Japan/8KI0040/2008/EPI_ISL_85155/NA/H3N5/2008-10-08
A/duck/Hokkaido/66/01/EPI_ISL_393/NA/H12N5/2001-07-01
A/duck/Hokkaido/1058/01/EPI_ISL_373/NA/H4N5/2001-07-01
A/T_Dk/NL-Werkendam/16014159-001/2016/EPI_ISL_287564/NA/H5N5/2016-11-14
A/mule_duck/Bulgaria/674/2010/EPI_ISL_174892/NA/H6N5/2010-04-12
A/mallard/Netherlands/11/2007/EPI_ISL_30812/NA/H6N5/2007-07-01
A/mallard_duck/Netherlands/13/2007/EPI_ISL_243404/NA/H6N5/2007-11-01
A/mallard_duck/Netherlands/4/2011/EPI_ISL_267409/NA/H12N5/2011-08-01
A/wild_duck/Korea/SH12-7/2008/EPI_ISL_133000/NA/H10N5/2008-11-15
A/duck/HuBei/03/2010/EPI_ISL_130353/NA/H5N5/2010-03-28
1985
1990
1995
2000
2005
2010
2015
2020

NP H5N5-GT2

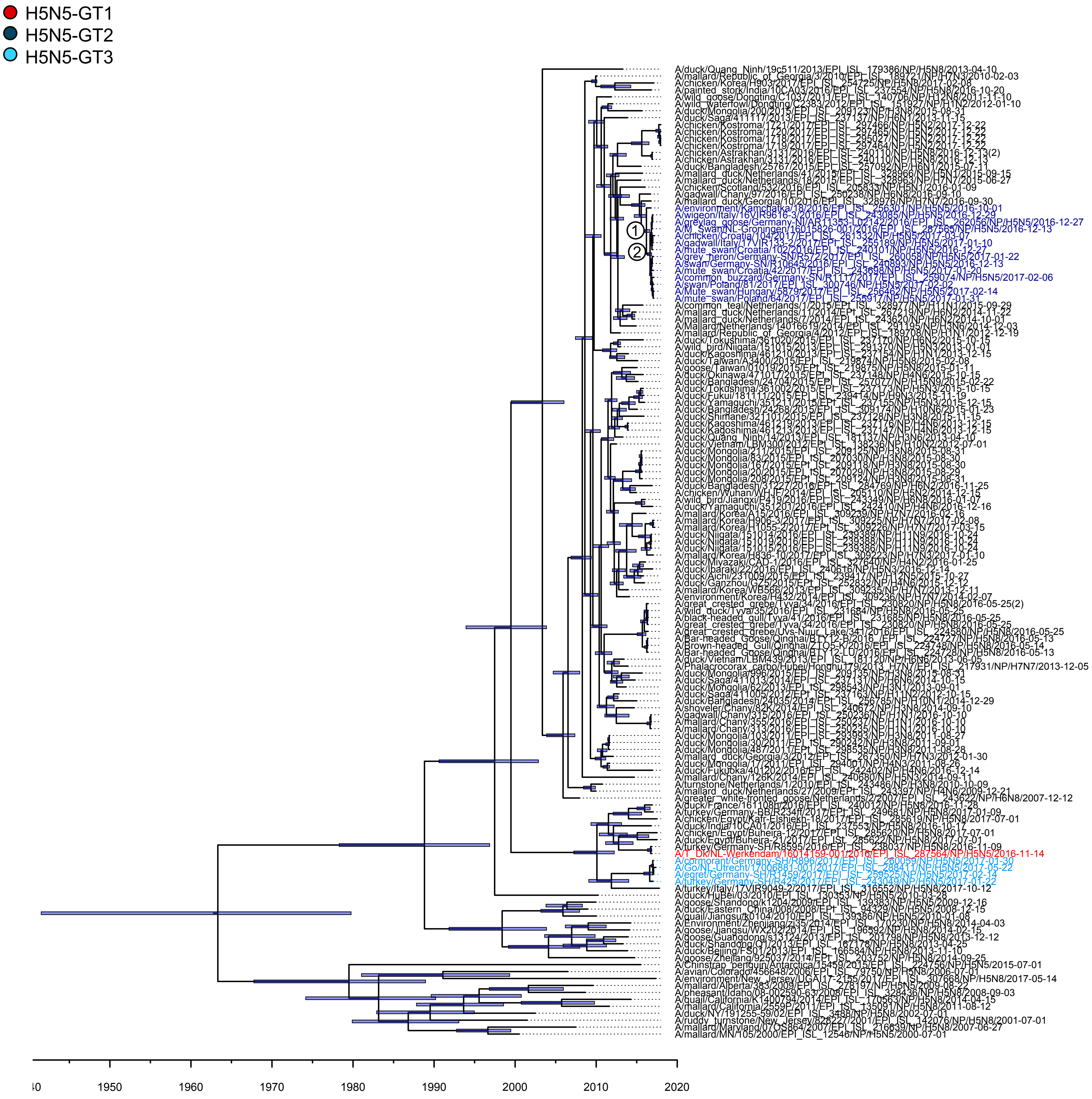
H5N5-GT1
H5N5-GT2
H5N5-GT3
1950
1960
1970
1980
1990
2000
2010
2020

NP H5N5-GT3

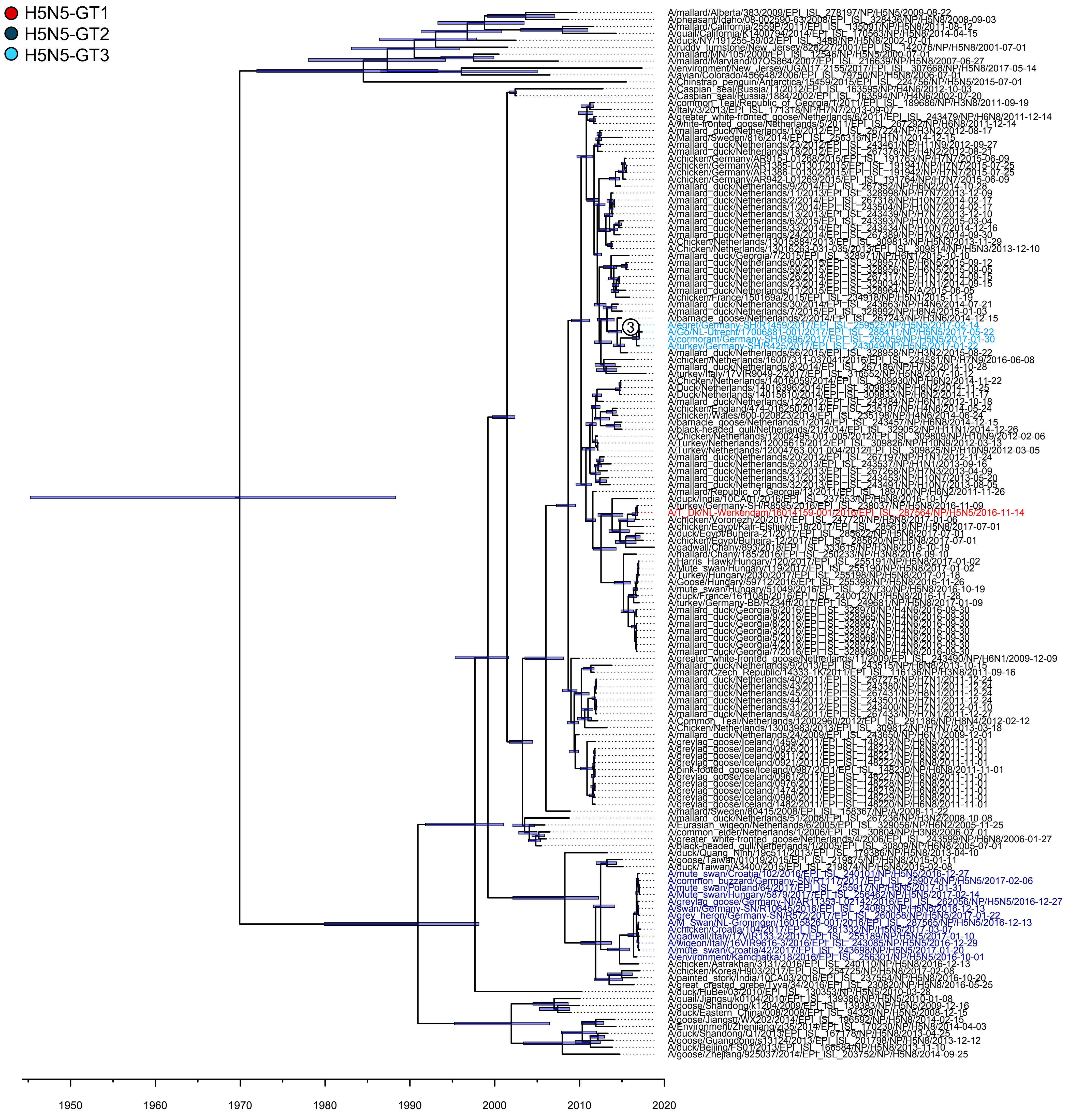
H5N5-GT1
H5N5-GT2
H5N5-GT3
1950
1960
1970
1980
1990
2000
2010
2020

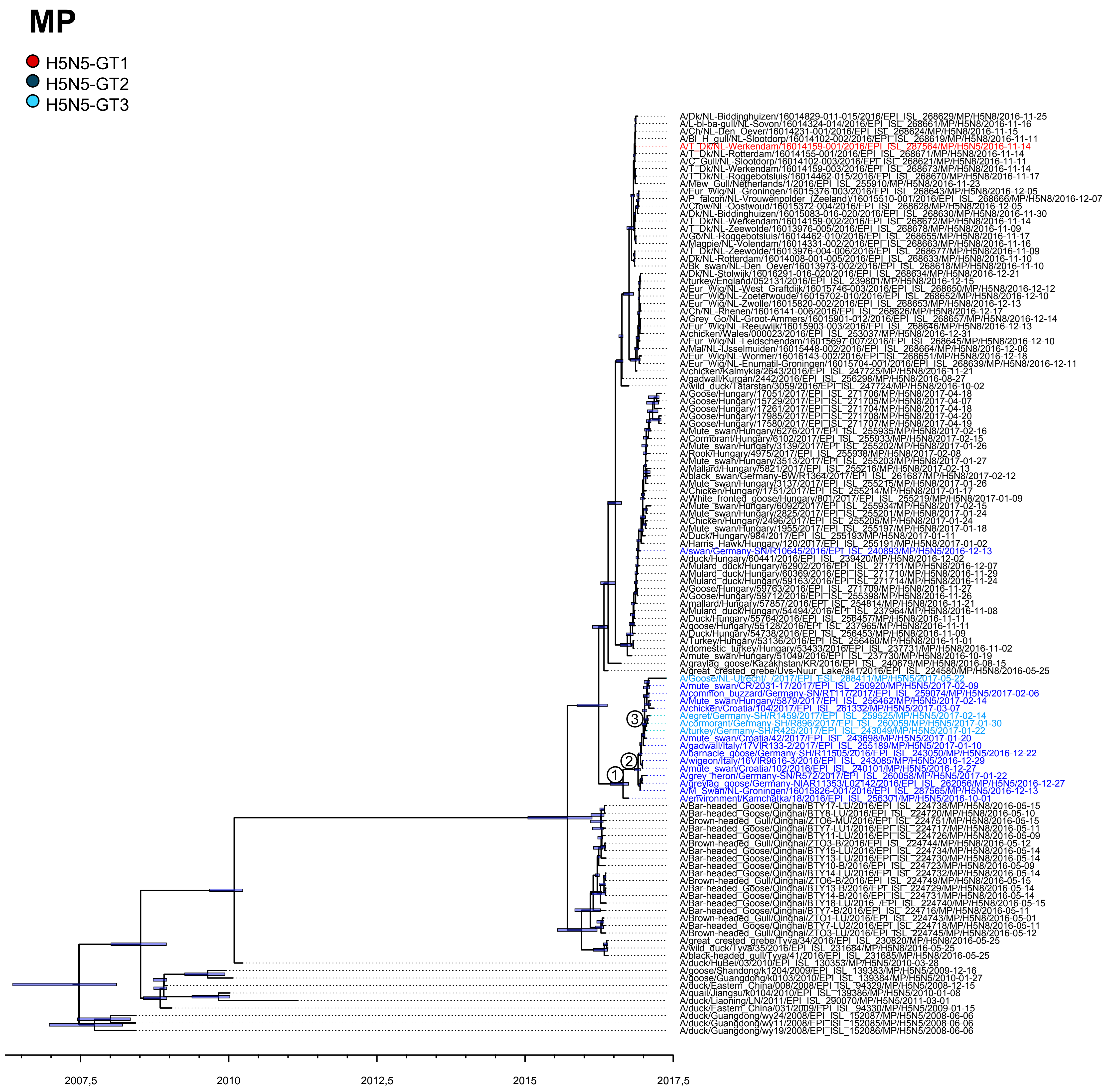

MP
H5N5-GT1
H5N5-GT2
H5N5-GT3
A/Dk/NL-Biddinghuizen/16014829-011-015/2016/EPI_ISL_268629/MP/H5N8/2016-11-25
A/L-bl-ba-gull/NL-Sovon/16014324-014/2016/EPI_ISL_268661/MP/H5N8/2016-11-16
A/Ch/NL-Den_Oever/16014231-001/2016/EPI_ISL_268624/MP/H5N8/2016-11-15
A/Bl_H_gull/NL-Slootdorp/16014102-002/2016/EPI_ISL_268619/MP/H5N8/2016-11-11
A/T_Dk/NL-Werkendam/16014159-001/2016/EPI_ISL_287564/MP/H5N5/2016-11-14
A/T_Dk/NL-Rotterdam/16014155-001/2016/EPI_ISL_268671/MP/H5N8/2016-11-14
A/C_Gull/NL-Slootdorp/16014102-003/2016/EPI_ISL_268621/MP/H5N8/2016-11-11
A/T_Dk/NL-Werkendam/16014159-003/2016/EPI_ISL_268673/MP/H5N8/2016-11-14
A/T_Dk/NL-Roggebotsluis/16014462-015/2016/EPI_ISL_268670/MP/H5N8/2016-11-17
A/Mew_Gull/Netherlands/1/2016/EPI_ISL_255910/MP/H5N8/2016-11-23
A/Eur_Wig/NL-Groningen/16015376-003/2016/EPI_ISL_268643/MP/H5N8/2016-12-05
A/P_falcon/NL-Vrouwenpolder_(Zeeland)/16015510-001/2016/EPI_ISL_268666/MP/H5N8/2016-12-07
A/Crow/NL-Oostwoud/16015372-004/2016/EPI_ISL_268628/MP/H5N8/2016-12-05
A/Dk/NL-Biddinghuizen/16015083-016-020/2016/EPI_ISL_268630/MP/H5N8/2016-11-30
A/T_Dk/NL-Werkendam/16014159-002/2016/EPI_ISL_268672/MP/H5N8/2016-11-14
A/T_Dk/NL-Zeewolde/16013976-005/2016/EPI_ISL_268678/MP/H5N8/2016-11-09
A/Go/NL-Roggebotsluis/16014462-010/2016/EPI_ISL_268655/MP/H5N8/2016-11-17
A/Magpie/NL-Volendam/16014331-002/2016/EPI_ISL_268663/MP/H5N8/2016-11-16
A/T_Dk/NL-Zeewolde/16013976-004-006/2016/EPI_ISL_268677/MP/H5N8/2016-11-09
A/Dk/NL-Rotterdam/16014008-001-005/2016/EPI_ISL_268633/MP/H5N8/2016-11-10
A/Bk_swan/NL-Den_Oever/16013973-002/2016/EPI_ISL_268618/MP/H5N8/2016-11-10
A/Dk/NL-Stolwijk/16016291-016-020/2016/EPI_ISL_268634/MP/H5N8/2016-12-21
A/turkey/England/052131/2016/EPI_ISL_239801/MP/H5N8/2016-12-15
A/Eur_Wig/NL-West_Graftdijk/16015746-003/2016/EPI_ISL_268650/MP/H5N8/2016-12-12
A/Eur_Wig/NL-Zoeterwoude/16015702-010/2016/EPI_ISL_268652/MP/H5N8/2016-12-10
A/Eur_Wig/NL-Zwolle/16015820-002/2016/EPI_ISL_268653/MP/H5N8/2016-12-13
A/Ch/NL-Rhenen/16016141-006/2016/EPI_ISL_268626/MP/H5N8/2016-12-17
A/Grey_Go/NL-Groot-Ammers/16015901-012/2016/EPI_ISL_268657/MP/H5N8/2016-12-14
A/Eur_Wig/NL-Reeuwijk/16015903-003/2016/EPI_ISL_268646/MP/H5N8/2016-12-13
A/chicken/Wales/000023/2016/EPI_ISL_253037/MP/H5N8/2016-12-31
A/Eur_Wig/NL-Leidschendam/16015697-007/2016/EPI_ISL_268645/MP/H5N8/2016-12-10
A/Mal/NL-IJsselmuiden/16015448-002/2016/EPI_ISL_268664/MP/H5N8/2016-12-06
A/Eur_Wig/NL-Wormer/16016143-002/2016/EPI_ISL_268651/MP/H5N8/2016-12-18
A/Eur_Wig/NL-Enumatil-Groningen/16015704-001/2016/EPI_ISL_268639/MP/H5N8/2016-12-11
A/chicken/Kalmykia/2643/2016/EPI_ISL_247725/MP/H5N8/2016-11-21
A/gadwall/Kurgan/2442/2016/EPI_ISL_256298/MP/H5N8/2016-08-27
A/wild_duck/Tatarstan/3059/2016/EPI_ISL_247724/MP/H5N8/2016-10-02
A/Goose/Hungary/17051/2017/EPI_ISL_271706/MP/H5N8/2017-04-18
A/Goose/Hungary/15729/2017/EPI_ISL_271705/MP/H5N8/2017-04-07
A/Goose/Hungary/17261/2017/EPI_ISL_271704/MP/H5N8/2017-04-18
A/Goose/Hungary/17985/2017/EPI_ISL_271708/MP/H5N8/2017-04-20
A/Goose/Hungary/17580/2017/EPI_ISL_271707/MP/H5N8/2017-04-19
A/Mute_swan/Hungary/6276/2017/EPI_ISL_255935/MP/H5N8/2017-02-16
A/Cormorant/Hungary/6102/2017/EPI_ISL_255933/MP/H5N8/2017-02-15
A/Mute_swan/Hungary/3139/2017/EPI_ISL_255202/MP/H5N8/2017-01-26
A/Rook/Hungary/4975/2017/EPI_ISL_255938/MP/H5N8/2017-02-08
A/Mute_swan/Hungary/3513/2017/EPI_ISL_255203/MP/H5N8/2017-01-27
A/Mallard/Hungary/5821/2017/EPI_ISL_255216/MP/H5N8/2017-02-13
A/black_swan/Germany-BW/R1364/2017/EPI_ISL_261687/MP/H5N8/2017-02-12
A/Mute_swan/Hungary/3137/2017/EPI_ISL_255215/MP/H5N8/2017-01-26
A/Chicken/Hungary/1751/2017/EPI_ISL_255214/MP/H5N8/2017-01-17
A/White_fronted_goose/Hungary/801/2017/EPI_ISL_255219/MP/H5N8/2017-01-09
A/Mute_swan/Hungary/6092/2017/EPI_ISL_255934/MP/H5N8/2017-02-15
A/Mute_swan/Hungary/2825/2017/EPI_ISL_255201/MP/H5N8/2017-01-24
A/Chicken/Hungary/2496/2017/EPI_ISL_255205/MP/H5N8/2017-01-24
A/Mute_swan/Hungary/1955/2017/EPI_ISL_255197/MP/H5N8/2017-01-18
A/Duck/Hungary/984/2017/EPI_ISL_255193/MP/H5N8/2017-01-11
A/Harris_Hawk/Hungary/120/2017/EPI_ISL_255191/MP/H5N8/2017-01-02
A/swan/Germany-SN/R10645/2016/EPI_ISL_240893/MP/H5N5/2016-12-13
A/duck/Hungary/60441/2016/EPI_ISL_239420/MP/H5N8/2016-12-02
A/Mulard_duck/Hungary/62902/2016/EPI_ISL_271711/MP/H5N8/2016-12-07
A/Mulard_duck/Hungary/60369/2016/EPI_ISL_271710/MP/H5N8/2016-11-29
A/Mulard_duck/Hungary/59163/2016/EPI_ISL_271714/MP/H5N8/2016-11-24
A/Goose/Hungary/59763/2016/EPI_ISL_271709/MP/H5N8/2016-11-27
A/Goose/Hungary/59712/2016/EPI_ISL_255398/MP/H5N8/2016-11-26
A/mallard/Hungary/57857/2016/EPI_ISL_254814/MP/H5N8/2016-11-21
A/Mulard_duck/Hungary/54494/2016/EPI_ISL_237964/MP/H5N8/2016-11-08
A/Duck/Hungary/55764/2016/EPI_ISL_256457/MP/H5N8/2016-11-11
A/goose/Hungary/55128/2016/EPI_ISL_237965/MP/H5N8/2016-11-11
A/Duck/Hungary/54738/2016/EPI_ISL_256453/MP/H5N8/2016-11-09
A/Turkey/Hungary/53136/2016/EPI_ISL_256460/MP/H5N8/2016-11-01
A/domestic_turkey/Hungary/53433/2016/EPI_ISL_237731/MP/H5N8/2016-11-02
A/mute_swan/Hungary/51049/2016/EPI_ISL_237730/MP/H5N8/2016-10-19
A/graylag_goose/Kazakhstan/KR/2016/EPI_ISL_240679/MP/H5N8/2016-08-15
A/great_crested_grebe/Uvs-Nuur_Lake/341/2016/EPI_ISL_224580/MP/H5N8/2016-05-25
A/Goose/NL-Utrecht/_/2017/EPI_ESL_288411/MP/H5N5/2017-05-22
A/mute_swan/CR/2031-17/2017/EPI_ISL_250920/MP/H5N5/2017-02-09
A/common_buzzard/Germany-SN/R1117/2017/EPI_ISL_259074/MP/H5N5/2017-02-06
A/Mute_swan/Hungary/5879/2017/EPI_ISL_256462/MP/H5N5/2017-02-14
A/chicken/Croatia/104/2017/EPI_ISL_261332/MP/H5N5/2017-03-07
A/egret/Germany-SH/R1459/2017/EPI_ISL_259525/MP/H5N5/2017-02-14
A/cormorant/Germany-SH/R896/2017/EPI_ISL_260059/MP/H5N5/2017-01-30
A/turkey/Germany-SH/R425/2017/EPI_ISL_243049/MP/H5N5/2017-01-22
A/mute_swan/Croatia/42/2017/EPI_ISL_243698/MP/H5N5/2017-01-20
A/gadwall/Italy/17VIR133-2/2017/EPI_ISL_255189/MP/H5N5/2017-01-10
A/barnacle_goose/Germany-SH/R11505/2016/EPI_ISL_243050/MP/H5N5/2016-12-22
A/wigeon/Italy/16VIR9616-3/2016/EPI_ISL_243085/MP/H5N5/2016-12-29
A/mute_swan/Croatia/102/2016/EPI_ISL_240101/MP/H5N5/2016-12-27
A/grey_heron/Germany-SN/R572/2017/EPI_ISL_260058/MP/H5N5/2017-01-22
A/greylag_goose/Germany-NIAR11353/L02142/2016/EPI_ISL_262056/MP/H5N5/2016-12-27
A/M_Swan/NL-Groningen/16015826-001/2016/EPI_ISL_287565/MP/H5N5/2016-12-13
A/environment/Kamchatka/18/2016/EPI_ISL_256301/MP/H5N5/2016-10-01
A/Bar-headed_Goose/Qinghai/BTY17-LU/2016/EPI_ISL_224738/MP/H5N8/2016-05-15
A/Bar-headed_Goose/Qinghai/BTY8-LU/2016/EPI_ISL_224720/MP/H5N8/2016-05-10
A/Brown-headed_Gull/Qinghai/ZTO6-MU/2016/EPI_ISL_224751/MP/H5N8/2016-05-15
A/Bar-headed_Goose/Qinghai/BTY7-LU1/2016/EPI_ISL_224717/MP/H5N8/2016-05-11
A/Bar-headed_Goose/Qinghai/BTY11-LU/2016/EPI_ISL_224726/MP/H5N8/2016-05-09
A/Brown-headed_Gull/Qinghai/ZTO3-B/2016/EPI_ISL_224744/MP/H5N8/2016-05-12
A/Bar-headed_Goose/Qinghai/BTY15-LU/2016/EPI_ISL_224734/MP/H5N8/2016-05-14
A/Bar-headed_Goose/Qinghai/BTY13-LU/2016/EPI_ISL_224730/MP/H5N8/2016-05-14
A/Bar-headed_Goose/Qinghai/BTY10-B/2016/EPI_ISL_224723/MP/H5N8/2016-05-09
A/Bar-headed_Goose/Qinghai/BTY14-LU/2016/EPI_ISL_224732/MP/H5N8/2016-05-14
A/Brown-headed_Gull/Qinghai/ZTO6-B/2016/EPI_ISL_224749/MP/H5N8/2016-05-15
A/Bar-headed_Goose/Qinghai/BTY13-B/2016/EPI_ISL_224729/MP/H5N8/2016-05-14
A/Bar-headed_Goose/Qinghai/BTY14-B/2016/EPI_ISL_224731/MP/H5N8/2016-05-14
A/Bar-headed_Goose/Qinghai/BTY18-LU/2016_/EPI_ISL_224740/MP/H5N8/2016-05-15
A/Bar-headed_Goose/Qinghai/BTY7-B/2016/EPI_ISL_224716/MP/H5N8/2016-05-11
A/Brown-headed_Gull/Qinghai/ZTO1-LU/2016/EPI_ISL_224743/MP/H5N8/2016-05-01
A/Bar-headed_Goose/Qinghai/BTY7-LU2/2016/EPI_ISL_224718/MP/H5N8/2016-05-11
A/Brown-headed_Gull/Qinghai/ZTO3-LU/2016/EPI_ISL_224745/MP/H5N8/2016-05-12
A/great_crested_grebe/Tyva/34/2016/EPI_ISL_230820/MP/H5N8/2016-05-25
A/wild_duck/Tyva/35/2016/EPI_ISL_231684/MP/H5N8/2016-05-25
A/black-headed_gull/Tyva/41/2016/EPI_ISL_231685/MP/H5N8/2016-05-25
A/duck/HuBei/03/2010/EPI_ISL_130353/MP/H5N5/2010-03-28
A/goose/Shandong/k1204/2009/EPI_ISL_139383/MP/H5N5/2009-12-16
A/goose/Guangdong/k0103/2010/EPI_ISL_139384/MP/H5N5/2010-01-27
A/duck/Eastern_China/008/2008/EPI_ISL_94329/MP/H5N5/2008-12-15
A/quail/Jiangsu/k0104/2010/EPI_ISL_139386/MP/H5N5/2010-01-08
A/duck/Liaoning/LN/2011/EPI_ISL_290070/MP/H5N5/2011-03-01
A/duck/Eastern_China/031/2009/EPI_ISL_94330/MP/H5N5/2009-01-15
A/duck/Guangdong/wy24/2008/EPI_ISL_152087/MP/H5N5/2008-06-06
A/duck/Guangdong/wy11/2008/EPI_ISL_152085/MP/H5N5/2008-06-06
A/duck/Guangdong/wy19/2008/EPI_ISL_152086/MP/H5N5/2008-06-06
1
2
3
2007,5
2010
2012,5
2015
2017,5

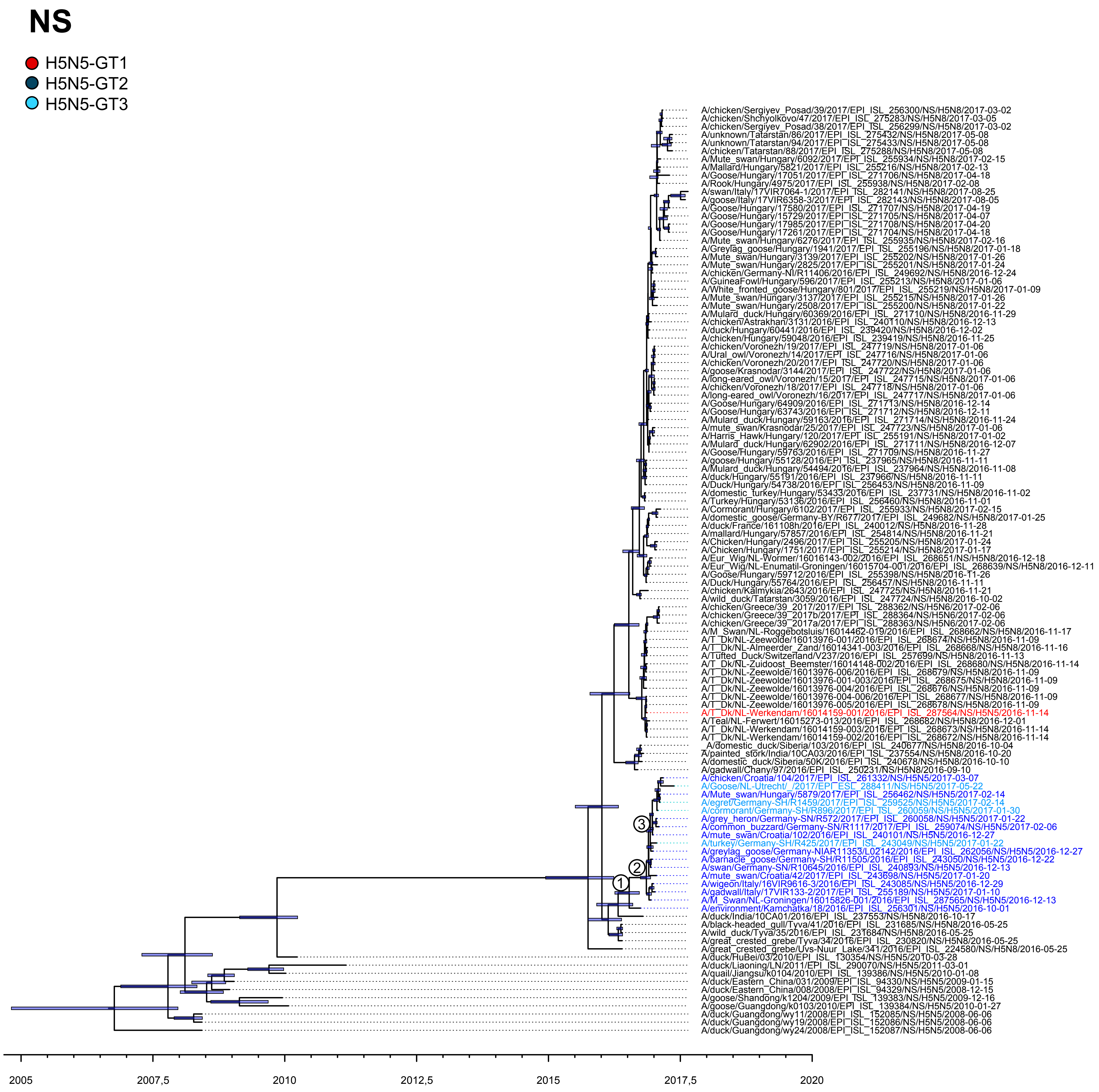

NS
H5N5-GT1
H5N5-GT2
H5N5-GT3
A/chicken/Sergiyev_Posad/39/2017/EPI_ISL_256300/NS/H5N8/2017-03-02
A/chicken/Shchyolkovo/47/2017/EPI_ISL_275283/NS/H5N8/2017-03-05
A/chicken/Sergiyev_Posad/38/2017/EPI_ISL_256299/NS/H5N8/2017-03-02
A/unknown/Tatarstan/86/2017/EPI_ISL_275432/NS/H5N8/2017-05-08
A/unknown/Tatarstan/94/2017/EPI_ISL_275433/NS/H5N8/2017-05-08
A/chicken/Tatarstan/88/2017/EPI_ISL_275288/NS/H5N8/2017-05-08
A/Mute_swan/Hungary/6092/2017/EPI_ISL_255934/NS/H5N8/2017-02-15
A/Mallard/Hungary/5821/2017/EPI_ISL_255216/NS/H5N8/2017-02-13
A/Goose/Hungary/17051/2017/EPI_ISL_271706/NS/H5N8/2017-04-18
A/Rook/Hungary/4975/2017/EPI_ISL_255938/NS/H5N8/2017-02-08
A/swan/Italy/17VIR7064-1/2017/EPI_ISL_282141/NS/H5N8/2017-08-25
A/goose/Italy/17VIR6358-3/2017/EPI_ISL_282143/NS/H5N8/2017-08-05
A/Goose/Hungary/17580/2017/EPI_ISL_271707/NS/H5N8/2017-04-19
A/Goose/Hungary/15729/2017/EPI_ISL_271705/NS/H5N8/2017-04-07
A/Goose/Hungary/17985/2017/EPI_ISL_271708/NS/H5N8/2017-04-20
A/Goose/Hungary/17261/2017/EPI_ISL_271704/NS/H5N8/2017-04-18
A/Mute_swan/Hungary/6276/2017/EPI_ISL_255935/NS/H5N8/2017-02-16
A/Greylag_goose/Hungary/1941/2017/EPI_ISL_255196/NS/H5N8/2017-01-18
A/Mute_swan/Hungary/3139/2017/EPI_ISL_255202/NS/H5N8/2017-01-26
A/Mute_swan/Hungary/2825/2017/EPI_ISL_255201/NS/H5N8/2017-01-24
A/chicken/Germany-NI/R11406/2016/EPI_ISL_249692/NS/H5N8/2016-12-24
A/GuineaFowl/Hungary/596/2017/EPI_ISL_255213/NS/H5N8/2017-01-06
A/White_fronted_goose/Hungary/801/2017/EPI_ISL_255219/NS/H5N8/2017-01-09
A/Mute_swan/Hungary/3137/2017/EPI_ISL_255215/NS/H5N8/2017-01-26
A/Mute_swan/Hungary/2508/2017/EPI_ISL_255200/NS/H5N8/2017-01-22
A/Mulard_duck/Hungary/60369/2016/EPI_ISL_271710/NS/H5N8/2016-11-29
A/chicken/Astrakhan/3131/2016/EPI_ISL_240110/NS/H5N8/2016-12-13
A/duck/Hungary/60441/2016/EPI_ISL_239420/NS/H5N8/2016-12-02
A/chicken/Hungary/59048/2016/EPI_ISL_239419/NS/H5N8/2016-11-25
A/chicken/Voronezh/19/2017/EPI_ISL_247719/NS/H5N8/2017-01-06
A/Ural_owl/Voronezh/14/2017/EPI_ISL_247716/NS/H5N8/2017-01-06
A/chicken/Voronezh/20/2017/EPI_ISL_247720/NS/H5N8/2017-01-06
A/goose/Krasnodar/3144/2017/EPI_ISL_247722/NS/H5N8/2017-01-06
A/long-eared_owl/Voronezh/15/2017/EPI_ISL_247715/NS/H5N8/2017-01-06
A/chicken/Voronezh/18/2017/EPI_ISL_247718/NS/H5N8/2017-01-06
A/long-eared_owl/Voronezh/16/2017/EPI_ISL_247717/NS/H5N8/2017-01-06
A/Goose/Hungary/64909/2016/EPI_ISL_271713/NS/H5N8/2016-12-14
A/Goose/Hungary/63743/2016/EPI_ISL_271712/NS/H5N8/2016-12-11
A/Mulard_duck/Hungary/59163/2016/EPI_ISL_271714/NS/H5N8/2016-11-24
A/mute_swan/Krasnodar/25/2017/EPI_ISL_247723/NS/H5N8/2017-01-06
A/Harris_Hawk/Hungary/120/2017/EPI_ISL_255191/NS/H5N8/2017-01-02
A/Mulard_duck/Hungary/62902/2016/EPI_ISL_271711/NS/H5N8/2016-12-07
A/Goose/Hungary/59763/2016/EPI_ISL_271709/NS/H5N8/2016-11-27
A/goose/Hungary/55128/2016/EPI_ISL_237965/NS/H5N8/2016-11-11
A/Mulard_duck/Hungary/54494/2016/EPI_ISL_237964/NS/H5N8/2016-11-08
A/duck/Hungary/55191/2016/EPI_ISL_237966/NS/H5N8/2016-11-11
A/Duck/Hungary/54738/2016/EPI_ISL_256453/NS/H5N8/2016-11-09
A/domestic_turkey/Hungary/53433/2016/EPI_ISL_237731/NS/H5N8/2016-11-02
A/Turkey/Hungary/53136/2016/EPI_ISL_256460/NS/H5N8/2016-11-01
A/Cormorant/Hungary/6102/2017/EPI_ISL_255933/NS/H5N8/2017-02-15
A/domestic_goose/Germany-BY/R677/2017/EPI_ISL_249682/NS/H5N8/2017-01-25
A/duck/France/161108h/2016/EPI_ISL_240012/NS/H5N8/2016-11-28
A/mallard/Hungary/57857/2016/EPI_ISL_254814/NS/H5N8/2016-11-21
A/Chicken/Hungary/2496/2017/EPI_ISL_255205/NS/H5N8/2017-01-24
A/Chicken/Hungary/1751/2017/EPI_ISL_255214/NS/H5N8/2017-01-17
A/Eur_Wig/NL-Wormer/16016143-002/2016/EPI_ISL_268651/NS/H5N8/2016-12-18
A/Eur_Wig/NL-Enumatil-Groningen/16015704-001/2016/EPI_ISL_268639/NS/H5N8/2016-12-11
A/Goose/Hungary/59712/2016/EPI_ISL_255398/NS/H5N8/2016-11-26
A/Duck/Hungary/55764/2016/EPI_ISL_256457/NS/H5N8/2016-11-11
A/chicken/Kalmykia/2643/2016/EPI_ISL_247725/NS/H5N8/2016-11-21
A/wild_duck/Tatarstan/3059/2016/EPI_ISL_247724/NS/H5N8/2016-10-02
A/chicken/Greece/39_2017/2017/EPI_ISL_288362/NS/H5N6/2017-02-06
A/chicken/Greece/39_2017b/2017/EPI_ISL_288364/NS/H5N6/2017-02-06
A/chicken/Greece/39_2017a/2017/EPI_ISL_288363/NS/H5N6/2017-02-06
A/M_Swan/NL-Roggebotsluis/16014462-019/2016/EPI_ISL_268662/NS/H5N8/2016-11-17
A/T_Dk/NL-Zeewolde/16013976-001/2016/EPI_ISL_268674/NS/H5N8/2016-11-09
A/T_Dk/NL-Almeerder_Zand/16014341-003/2016/EPI_ISL_268668/NS/H5N8/2016-11-16
A/Tufted_Duck/Switzerland/V237/2016/EPI_ISL_257699/NS/H5N8/2016-11-13
A/T_Dk/NL-Zuidoost_Beemster/16014148-002/2016/EPI_ISL_268680/NS/H5N8/2016-11-14
A/T_Dk/NL-Zeewolde/16013976-006/2016/EPI_ISL_268679/NS/H5N8/2016-11-09
A/T_Dk/NL-Zeewolde/16013976-001-003/2016/EPI_ISL_268675/NS/H5N8/2016-11-09
A/T_Dk/NL-Zeewolde/16013976-004/2016/EPI_ISL_268676/NS/H5N8/2016-11-09
A/T_Dk/NL-Zeewolde/16013976-004-006/2016/EPI_ISL_268677/NS/H5N8/2016-11-09
A/T_Dk/NL-Zeewolde/16013976-005/2016/EPI_ISL_268678/NS/H5N8/2016-11-09
A/T_Dk/NL-Werkendam/16014159-001/2016/EPI_ISL_287564/NS/H5N5/2016-11-14
A/Teal/NL-Ferwert/16015273-013/2016/EPI_ISL_268682/NS/H5N8/2016-12-01
A/T_Dk/NL-Werkendam/16014159-003/2016/EPI_ISL_268673/NS/H5N8/2016-11-14
A/T_Dk/NL-Werkendam/16014159-002/2016/EPI_ISL_268672/NS/H5N8/2016-11-14
A/domestic_duck/Siberia/103/2016/EPI_ISL_240677/NS/H5N8/2016-10-04
A/painted_stork/India/10CA03/2016/EPI_ISL_237554/NS/H5N8/2016-10-20
A/domestic_duck/Siberia/50K/2016/EPI_ISL_240678/NS/H5N8/2016-10-10
A/gadwall/Chany/97/2016/EPI_ISL_250231/NS/H5N8/2016-09-10
A/chicken/Croatia/104/2017/EPI_ISL_261332/NS/H5N5/2017-03-07
A/Goose/NL-Utrecht/_/2017/EPI_ESL_288411/NS/H5N5/2017-05-22
A/Mute_swan/Hungary/5879/2017/EPI_ISL_256462/NS/H5N5/2017-02-14
A/egret/Germany-SH/R1459/2017/EPI_ISL_259525/NS/H5N5/2017-02-14
A/cormorant/Germany-SH/R896/2017/EPI_ISL_260059/NS/H5N5/2017-01-30
A/grey_heron/Germany-SN/R572/2017/EPI_ISL_260058/NS/H5N5/2017-01-22
A/common_buzzard/Germany-SN/R1117/2017/EPI_ISL_259074/NS/H5N5/2017-02-06
A/mute_swan/Croatia/102/2016/EPI_ISL_240101/NS/H5N5/2016-12-27
A/turkey/Germany-SH/R425/2017/EPI_ISL_243049/NS/H5N5/2017-01-22
A/greylag_goose/Germany-NIAR11353/L02142/2016/EPI_ISL_262056/NS/H5N5/2016-12-27
A/barnacle_goose/Germany-SH/R11505/2016/EPI_ISL_243050/NS/H5N5/2016-12-22
A/swan/Germany-SN/R10645/2016/EPI_ISL_240893/NS/H5N5/2016-12-13
A/mute_swan/Croatia/42/2017/EPI_ISL_243698/NS/H5N5/2017-01-20
A/wigeon/Italy/16VIR9616-3/2016/EPI_ISL_243085/NS/H5N5/2016-12-29
A/gadwall/Italy/17VIR133-2/2017/EPI_ISL_255189/NS/H5N5/2017-01-10
A/M_Swan/NL-Groningen/16015826-001/2016/EPI_ISL_287565/NS/H5N5/2016-12-13
A/environment/Kamchatka/18/2016/EPI_ISL_256301/NS/H5N5/2016-10-01
A/duck/India/10CA01/2016/EPI_ISL_237553/NS/H5N8/2016-10-17
A/black-headed_gull/Tyva/41/2016/EPI_ISL_231685/NS/H5N8/2016-05-25
A/wild_duck/Tyva/35/2016/EPI_ISL_231684/NS/H5N8/2016-05-25
A/great_crested_grebe/Tyva/34/2016/EPI_ISL_230820/NS/H5N8/2016-05-25
A/great_crested_grebe/Uvs-Nuur_Lake/341/2016/EPI_ISL_224580/NS/H5N8/2016-05-25
A/duck/HuBei/03/2010/EPI_ISL_130354/NS/H5N5/2010-03-28
A/duck/Liaoning/LN/2011/EPI_ISL_290070/NS/H5N5/2011-03-01
A/quail/Jiangsu/k0104/2010/EPI_ISL_139386/NS/H5N5/2010-01-08
A/duck/Eastern_China/031/2009/EPI_ISL_94330/NS/H5N5/2009-01-15
A/duck/Eastern_China/008/2008/EPI_ISL_94329/NS/H5N5/2008-12-15
A/goose/Shandong/k1204/2009/EPI_ISL_139383/NS/H5N5/2009-12-16
A/goose/Guangdong/k0103/2010/EPI_ISL_139384/NS/H5N5/2010-01-27
A/duck/Guangdong/wy11/2008/EPI_ISL_152085/NS/H5N5/2008-06-06
A/duck/Guangdong/wy19/2008/EPI_ISL_152086/NS/H5N5/2008-06-06
A/duck/Guangdong/wy24/2008/EPI_ISL_152087/NS/H5N5/2008-06-06
3
2
1
2005
2007,5
2010
2012,5
2015
2017,5
2020
