# Supplementary material for: Spread of Highly Pathogenic Avian Influenza (HPAI) H5N5 Viruses in Europe in 2016–2017 Appears Related to the Timing of Reassortment Events
Source: Viruses. 2019 May 31;11(6):501. doi: 10.3390/v11060501 (PMC6631432; doi:10.3390/v11060501)
Supplement: Supplementary file 1 [file viruses-11-00501-s001.zip › Table S1_revised.pdf]

Table S1. Viruses most closely related to the newly isolated H5N5 strain. Genetically most closely related viruses to the newly isolated highly pathogenic avian influenza (HPAI) H5N5-GT3 virus strain (A/Go/NL-Utrecht/17006881-001/2017; H5N5-19) as identified for each gene segment by BLAST in the GISAID's EpiFlu™ Database (http://www.gisaid.org) on 10 May 2019.

Gene	Most closely related virus	Subtype	GISAID isolate ID	Nucleotide sequence identity
PB2	A/M_Swan/NL-Groningen/16015826-001/2016	H5N5	EPI_ISL_287565	2311/2316 (99.8%)
PB1	A/egret/Germany-SH/R1459/2017	H5N5	EPI_ISL_259525	2281/2286 (99.8%)
PA	A/M_Swan/NL-Groningen/16015826-001/2016	H5N5	EPI_ISL_287565	2203/2208 (99.8%)
	A/Mute_swan/Hungary/5879/2017	H5N5	EPI_ISL_256462	
HA	A/M_Swan/NL-Groningen/16015826-001/2016	H5N5	EPI_ISL_287565	1746/1751 (99.7%)
NP	A/cormorant/Germany-SH/R896/2017	H5N5	EPI_ISL_260059	1507/1507 (100.0%)
	A/egret/Germany-SH/R1459/2017	H5N5	EPI_ISL_259525	
NA	A/M_Swan/NL-Groningen/16015826-001/2016	H5N5	EPI_ISL_287565	1434/1441 (99.5%)
	A/Mute_swan/Hungary/5879/2017	H5N5	EPI_ISL_256462	
MP	A/Mute_swan/Hungary/5879/2017	H5N5	EPI_ISL_256462	1002/1002 (100.0%)
NS	A/cormorant/Germany-SH/R896/2017	H5N5	EPI_ISL_260059	864/865 (99.9%)
	A/egret/Germany-SH/R1459/2017	H5N5	EPI_ISL_259525	
	A/Mute_swan/Hungary/5879/2017	H5N5	EPI_ISL_256462	

PB2, polymerase basic protein 2; PB1, polymerase basic protein 1; PA, polymerase acidic protein; HA, hemagglutinin; NP, nucleoprotein; NA, neuraminidase; MP, matrix protein; NS, nonstructural protein
