# Supplementary material for: Spread of Highly Pathogenic Avian Influenza (HPAI) H5N5 Viruses in Europe in 2016–2017 Appears Related to the Timing of Reassortment Events
Source: Viruses. 2019 May 31;11(6):501. doi: 10.3390/v11060501 (PMC6631432; doi:10.3390/v11060501)
Supplement: Supplementary file 1 [file viruses-11-00501-s001.zip › Table S2_revised.pdf]

Table S2. Detailed information on H5N5 virus sequences. Sequences of highly pathogenic avian influenza (HPAI) H5N5 viruses used in this study as listed in the GISAID's EpiFlu™ Database. We gratefully acknowledge the authors, originating and submitting laboratories of the sequences from GISAID's EpiFlu™ Database on which this research is based. All submitters of data may be contacted directly via the GISAID website (http://www.gisaid.org).

Short isolate name	Full isolate name	Bird species	Collection date	Collection location	Submitting laboratory	GISAID isolate ID
H5N5-1	A/T_Dk/NL-Werkendam/16014159-001/2016	Tufted duck	2016-11-14	Netherlands	Wageningen Bioveterinary Research	EPI_ISL_287564
H5N5-2	A/Environment/Kamchatka/18/2016	Environment	2016-10-01	Russia	State Research Center of Virology and Biotechnology VECTOR	EPI_ISL_256301
H5N5-3	A/Swan/Germany-SN/R10645/2016	Swan	2016-12-13	Germany	Friedrich-Loeffler-Institut	EPI_ISL_240893
H5N5-4	A/M_Swan/NL-Groningen/16015826-001/2016	Mute swan	2016-12-13	Netherlands	Wageningen Bioveterinary Research	EPI_ISL_287565
H5N5-5	A/Barnacle goose/Germany-SH/R11505/2016	Barnacle goose	2016-12-22	Germany	Friedrich-Loeffler-Institut	EPI_ISL_243050
H5N5-6	A/Greylag goose/Germany-NIAR11353-L02142/2016	Greylag goose	2016-12-27	Germany	Friedrich-Loeffler-Institut	EPI_ISL_262056
H5N5-7	A/Mute swan/Croatia/102/2016	Mute swan	2016-12-27	Croatia	Croatian Veterinary Institute	EPI_ISL_240101
H5N5-8	A/Wigeon/Italy/16VIR9616-3/2016	Wigeon	2016-12-29	Italy	Istituto Zooprofilattico Sperimentale Delle Venezie	EPI_ISL_243085
H5N5-9	A/Gadwall/Italy/17VIR133-2/2017	Gadwall	2017-01-10	Italy	Istituto Zooprofilattico Sperimentale Delle Venezie	EPI_ISL_255189
H5N5-10	A/Mute swan/Croatia/42/2017	Mute swan	2017-01-20	Croatia	Croatian Veterinary Institute	EPI_ISL_243698
H5N5-11	A/Grey heron/Germany-SN/R572/2017	Grey heron	2017-01-22	Germany	Friedrich-Loeffler-Institut	EPI_ISL_260058
H5N5-12	A/Mute swan/Poland/64/2017	Mute swan	2017-01-31	Poland	National Veterinary Research Institut Poland	EPI_ISL_255917
H5N5-13	A/Common buzzard/Germany-SN/R1117/2017	Common buzzard	2017-02-06	Germany	Friedrich-Loeffler-Institut	EPI_ISL_259074
H5N5-14	A/Mute swan/Hungary/5879/2017	Mute swan	2017-02-14	Hungary	Central Agricultural Office Veterinary Diagnostic Directorate	EPI_ISL_256462
H5N5-15	A/Chicken/Croatia/104/2017	Chicken	2017-03-07	Croatia	Croatian Veterinary Institute	EPI_ISL_261332
H5N5-16	A/Turkey/Germany-SH/R425/2017	Turkey	2017-01-22	Germany	Friedrich-Loeffler-Institut	EPI_ISL_243049
H5N5-17	A/Cormorant/Germany-SH/R896/2017	Cormorant	2017-01-30	Germany	Friedrich-Loeffler-Institut	EPI_ISL_260059
H5N5-18	A/Egret/Germany-SH/R1459/2017	Egret	2017-02-14	Germany	Friedrich-Loeffler-Institut	EPI_ISL_259525
H5N5-19	A/Go/NL-Utrecht/17006881-001/2017	Goose	2017-05-22	Netherlands	Wageningen Bioveterinary Research	EPI_ISL_288411
H5N5-20	A/Mute swan/Czech Republic/2031-17/2017	Mute swan	2017-02-09	Czech Republic	State Veterinary Institute Prague	EPI_ISL_250920
H5N5-21	A/Spot-billed pelican/Czech Republic/2270-17/2017	Spot-billed pelican	2017-02-14	Czech Republic	State Veterinary Institute Prague	EPI_ISL_268981
