# Supplementary material for: Spread of Highly Pathogenic Avian Influenza (HPAI) H5N5 Viruses in Europe in 2016–2017 Appears Related to the Timing of Reassortment Events
Source: Viruses. 2019 May 31;11(6):501. doi: 10.3390/v11060501 (PMC6631432; doi:10.3390/v11060501)
Supplement: Supplementary file 1 [file viruses-11-00501-s001.zip › Table S3_revised.pdf]

Table S3. List of H5N8 cluster representatives. Sequences of highly pathogenic avian influenza (HPAI) H5N8 viruses used as cluster representatives in this study as listed in the GISAID's EpiFlu™ Database. The cluster representatives were selected by clustering the nucleotide sequences of around 675 HPAI H5N8 2016-2017 viruses available in the GISAID's EpiFlu Database on 10 May 2019. A nucleotide sequence identity threshold value of 1.5% was used to define clusters. We gratefully acknowledge the authors, originating and submitting laboratories of the sequences from GISAID's EpiFlu™ Database on which this research is based. All submitters of data may be contacted directly via the GISAID website (http://www.gisaid.org).

Gene segment	Cluster representative	GISAID isolate ID	No. sequences in cluster
PB1	A/Brown-headed_Gull/Qinghai/ZTO1-B/2016	EPI_ISL_224742	436
	A/Goose/Hungary/65817/2016	EPI_ISL_255174	18
PB2	A/Brown-headed_Gull/Qinghai/ZTO1-B/2016	EPI_ISL_224742	97
	A/T_Dk/NL-Zeewolde/16013976-001/2016	EPI_ISL_268674	261
	A/mute_swan/Hungary/51049/2016	EPI_ISL_237730	103
PA	A/Brown-headed_Gull/Qinghai/ZTO1-B/2016	EPI_ISL_224742	174
	A/T_Dk/NL-Monnickendam/16013865-006-008/2016	EPI_ISL_268669	15
	A/T_Dk/NL-Zeewolde/16013976-001/2016	EPI_ISL_268674	241
	A/domestic_duck/Siberia/50K/2016	EPI_ISL_240678	62
	A/chicken/Taiwan/x37/2016	EPI_ISL_256398	2
NP	A/Brown-headed_Gull/Qinghai/ZTO1-B/2016	EPI_ISL_224742	62
	A/T_Dk/NL-Zeewolde/16013976-001/2016	EPI_ISL_268674	260
	A/duck/India/10CA01/2016	EPI_ISL_237553	1
	A/domestic_duck/Siberia/50K/2016	EPI_ISL_240678	69
	A/mute_swan/Hungary/51049/2016	EPI_ISL_237730	101
	A/chicken/Astrakhan/3131/2016	EPI_ISL_240110	1
HA	A/Brown-headed_Gull/Qinghai/ZTO1-B/2016	EPI_ISL_224742	675
MP	A/Brown-headed_Gull/Qinghai/ZTO1-B/2016	EPI_ISL_224742	490
NS	A/Brown-headed_Gull/Qinghai/ZTO1-B/2016	EPI_ISL_224742	480

PB2, polymerase basic protein 2; PB1, polymerase basic protein 1; PA, polymerase acidic protein; HA, hemagglutinin; NP, nucleoprotein; MP, matrix protein; NS, nonstructural protein
