# Supplementary material for: Spread of Highly Pathogenic Avian Influenza (HPAI) H5N5 Viruses in Europe in 2016–2017 Appears Related to the Timing of Reassortment Events
Source: Viruses. 2019 May 31;11(6):501. doi: 10.3390/v11060501 (PMC6631432; doi:10.3390/v11060501)
Supplement: Supplementary file 1 [file viruses-11-00501-s001.zip › Table S4_revised.pdf]

Table S4. Viruses used for *in vitro* experiments. List of highly pathogenic avian influenza (HPAI) H5N8 and H5N5 viruses used for *in vitro* experiments, representing genotypes H5N5-GT1, H5N5-GT2, H5N5-GT3 and European H5N8 NL-Zeewolde-like virus. The GISAID isolate ID numbers refer to the whole genome sequences that were submitted to the GISAID's EpiFlu™ Database (http://www.gisaid.org).

Genotype	Isolate name	Bird species	Collection date	Collection location	GISAID isolate ID
H5N8	A/Dk/NL-Biddinghuizen/16014829-011-015/2016	Domestic duck	2016-11-25	Biddinghuizen, The Netherlands	EPI_ISL_268629
H5N5-GT1	A/T_Dk/NL-Werkendam/16014159-001/2016	Tufted duck	2016-11-14	Werkendam, The Netherlands	EPI_ISL_287564
H5N5-GT2	A/M_Swan/NL-Groningen/16015826-001/2016	Mute swan	2016-12-13	Groningen, The Netherlands	EPI_ISL_287565
H5N5-GT3	A/Go/NL-Utrecht/17006881-001/2017	Goose	2017-05-22	Utrecht, The Netherlands	EPI_ISL_288411
