# Supplementary material for: Spread of Highly Pathogenic Avian Influenza (HPAI) H5N5 Viruses in Europe in 2016–2017 Appears Related to the Timing of Reassortment Events
Source: Viruses. 2019 May 31;11(6):501. doi: 10.3390/v11060501 (PMC6631432; doi:10.3390/v11060501)
Supplement: Supplementary file 1 [file viruses-11-00501-s001.zip › Table S5_revised.pdf]

Table S5. LPAI ancestor viruses of reassortant genes. Novel genes were obtained by reassortment of the (HPAI) H5N5 genotypes detected during the HPAI H5 2016-2017 epizootic (H5N5-GT1, H5N5-GT2 and H5N5-GT3) with co-circulating low pathogenic avian influenza (LPAI) ancestor viruses. The most closely related LPAI ancestor viruses were identified by BLAST in the GISAID's EpiFlu™ Database (http://www.gisaid.org) on 10 May 2019.

Genotype	Gene	Most closely related virus	Subtype	GISAID isolate ID	Nucleotide sequence identity
H5N5-GT1	PA	A/duck/Mongolia/17/2011	H4N3	EPI_ISL_294001	2174/2209 (98%)
	NA	A/Mallard duck/Sweden/139899/2012	H15N5	EPI_ISL_267231	1410/1442 (97%)
H5N5-GT2	PB2	A/Barnacle goose/Netherlands/2/2014	H3N6	EPI_ISL_267243	2294/2316 (99%)
	PB1	A/Duck/Mongolia/709/2015	H10N7	EPI_ISL_206696	2288/2316 (98%)
	NP	A/Mallard duck/Netherlands/7/2014	H6N2	EPI_ISL_243620	1519/1540 (98%)
	NA	A/duck/Kyoto/261007/2014	H6N5	EPI_ISL_237150	1418/1439 (98%)
H5N5-GT3	PB1	A/mallard duck/Netherlands/9/2014	H6N2	EPI_ISL_267352	2279/2316 (98%)
	NP	/Barnacle goose/Netherlands/2/2014	H3N6	EPI_ISL_267243	1525/1540 (99%)

PB2, polymerase basic protein 2; PB1, polymerase basic protein 1; NP, nucleoprotein; NA, neuraminidase.
